# Burden of esophageal cancer in global, regional and national regions from 1990 to 2021 and its projection until 2050: results from the GBD study 2021

**DOI:** 10.3389/fonc.2024.1518567

**Published:** 2025-01-20

**Authors:** Chengcheng Zhang, Linzhi Chen, Yuqi Xiu, Hongling Zhang, Yuejuan Zhang, Wenjuan Ying

**Affiliations:** ^1^ Institute of Nursing Research, The First Affiliated Hospital of Shantou University Medical College, Shantou, Guangdong, China; ^2^ Department of Nursing, Shantou University Medical College, Shantou, Guangdong, China; ^3^ Nursing Research Office, The First Affiliated Hospital of Hunan University of Chinese Medicine, Changsha, Hunan, China

**Keywords:** esophageal cancer, epidemiology, incidence, mortality, trend in global burden, risk factor, projection

## Abstract

**Background:**

Esophageal cancer (EC) is a major global health issue characterized by high morbidity and mortality rates, with a notably low five-year survival rate. Comprehensive analyses of the global burden of EC remain limited and outdated, despite its global significance. This study aimed to systematically assess the global burden and trends of esophageal cancer across diverse populations.

**Methods:**

Data on the burden of EC were collected from the Global Burden of Disease (GBD) 2021 study, including estimates of incidence, mortality, and disability-adjusted life years (DALYs), as well as risk factors, spanning 204 countries and territories. Age-standardized rates (ASRs) were calculated to allow comparisons across populations. The study further explored the relationship between EC burden and socioeconomic development by utilizing the Socio-demographic Index (SDI), aggregating data by regions. The Bayesian age-period-cohort model was applied to project future trends until 2050.

**Results:**

In 2021, there were 576,529 new esophageal cancer cases, with an age-standardized incidence rate (ASIR) of 6.65 per 100,000, reflecting a 24.87% decrease since 1990. The global number of deaths reached 538,602, with an age-standardized death rate (ASDR) of 6.25 per 100,000, representing a 30.67% decline. DALYs totaled 12,999,264, corresponding to an estimated annual percentage change (EAPC) of a 1.73% decrease in the age-standardized DALYs rate. East Asia accounted for nearly two-thirds of global EC cases and deaths, while Central Sub-Saharan Africa recorded the highest ASIR and ASDR. Central Asia experienced the largest reductions, whereas Western Sub-Saharan Africa showed increasing trends. Middle-SDI countries, such as Malawi and Lesotho, had disproportionately high burdens, while high-SDI countries, including Tunisia and Kuwait, had lower burdens. Males had higher incidence and mortality rates across all age groups. By 2050, the ASIR is projected to decrease to 6.17 per 100,000, and the ASDR to 5.23 per 100,000, though the absolute number of cases and deaths is expected to rise.

**Conclusions:**

The global burden of EC remains significant, with ongoing challenges in regions such as Africa and East Asia. These findings highlight the need for sustained and targeted prevention efforts, particularly in high-risk populations, to address the increasing absolute number of cases and deaths.

## 1 Introduction

Esophageal cancer (EC) remains a significant global health challenge due to its high morbidity and mortality rates (1). In 2020, EC was the seventh most commonly diagnosed cancer and the sixth leading cause of cancer-related deaths worldwide, accounting for approximately 604,000 new cases and 544,000 deaths (2). The morbidity and histological subtypes of EC vary markedly across geographic regions, influenced by socioeconomic factors (3, 4). Notably, age-standardized rates (ASRs) of EC are inversely correlated with the Socio-demographic Index (SDI), highlighting the need for region-specific prevention and management strategies (5). Despite advancements in diagnostic and therapeutic approaches, the overall prognosis for EC remains poor, with a global five-year survival rate below 20% (6). A comprehensive understanding of the global burden, risk factors, and epidemiological trends of EC, stratified by age and sex, is crucial for developing targeted interventions.

In terms of histological subtypes, the two primary forms of EC are esophageal squamous cell carcinoma (ESCC) and esophageal adenocarcinoma (EAC) (7). ESCC accounts for approximately 90% of all cases, with the highest morbidity rates observed in Eastern Asia and Southern Africa (8, 9). China, representing nearly 20% of the global population, contributes to over half of the worldwide EC cases and deaths (10). The disease disproportionately affects males, with male-to-female morbidity and mortality ratios of 2:1 and 3:1, respectively (11). Most EC cases are diagnosed at advanced stages, presenting significant challenges for effective treatment (12). While the global incidence of EC has generally declined over the past few decades, certain regions have experienced stable or even increasing rates (13–16). Currently, comprehensive global assessments of the EC burden by risk factors and recent epidemiological trends are limited (17). A detailed analysis of EC incidence, mortality, and disability-adjusted life years (DALYs) across different world regions is essential for informing public health strategies and allocating medical resources efficiently.

This study utilized data from the Global Burden of Disease Study 2021 (GBD 2021) to investigate global variations in EC incidence, mortality, and DALYs by region, country, SDI, age, and sex from 1990 to 2021. The GBD study provides essential metrics, including incidence, mortality, years of life lost (YLLs), years lived with disability (YLDs), and DALYs, serving as a comprehensive platform for understanding global disease patterns (18). GBD 2021 encompasses 369 diseases and injuries, alongside 87 risk factors, across 204 countries and territories, aiming to inform global health strategies and elucidate the causes of disease burden (19, 20). Although previous studies utilizing GBD data assessed the burden of EC up to 2019 (21, 22), they did not leverage the advanced methodologies and predictive analyses introduced in GBD 2021. Our study capitalizes on these advancements-including improved data quality, increased geographical coverage, and advanced predictive modeling techniques-to deliver a more thorough and updated analysis of the global EC burden from 1990 to 2021 (23, 24). By addressing these gaps, we aim to provide insights that will inform future research and contribute to the development of effective strategies to combat EC worldwide.

## 2 Methods

### 2.1 Data sources

The GBD 2021, conducted by the Institute for Health Metrics and Evaluation (IHME), is the most extensive and comprehensive epidemiological assessment of global disease burdens and trends to date. Data were obtained from the GBD 2021 resources using a standardized query tool. This dataset includes estimated incidence, prevalence, mortality, DALYs, and risk factors for 371 diseases and injuries across 204 countries and territories. In GBD 2021, cancers were classified according to the International Classification of Diseases, Tenth Revision (ICD-10), with EC categorized under codes C15.0 to C15.9. To facilitate meaningful comparisons across populations, age standardization was applied using the world standard population developed by Segi and modified by Doll et al. (25, 26). All estimates are presented as ASRs. The SDI, ranging from 0 to 1, was incorporated to explore associations between EC trends and development levels. Within the framework of GBD, the world was divided into seven super-regions and 21 GBD regions based on geography. Additionally, countries are categorized into five SDI quintiles (The regional division of SDI can be obtained from Institute for Health Metrics and Evaluation: https://ghdx.healthdata.org/search/site/SDI): high SDI, high-middle SDI, middle SDI, low-middle SDI, and low SDI. The SDI summarizes a country’s level of development based on total fertility rate, mean income per capita, and average years of schooling (27).

### 2.2 Statistical analysis

This study utilized data on EC incidence, mortality, and DALYs from the Global Health Data Exchange (GHDx) platform to quantify the global burden of disease. SDI data were integrated to assess the influence of socioeconomic factors on disease burden. Geographical data were aggregated by regions defined by the GBD study, and comparative analyses were conducted across these regions. Visualization of the global distribution and regional variations in disease burden was performed using R (version 4.2.3) with the ‘ggplot2’ and ‘sf’ packages, focusing on population-level differences stratified by age and sex.

To examine the association between SDI and esophageal cancer burden, disease rates were calculated across five SDI categories (ranging from low to high), utilizing the ‘dplyr’ and ‘ggplot2’ packages for data processing and visualization. Risk factors contributing to the esophageal cancer burden were analyzed based on GBD 2021 data, with attributable DALYs visualized through forest plots created using the ‘forestplot’ package.

For trend analysis, the percentage change (PC) in annual incidence, mortality, and DALYs was calculated as the primary indicator of burden variation. This metric captures the relative change in disease rates over a specified time period in a particular region or population, reflecting trends in the burden of disease. To more precisely and consistently assess temporal trends, the estimated annual percentage change (EAPC) was used, providing a standardized method to quantify the direction and magnitude of changes in ASRs over time. The ASRs were calculated using the GBD standard, with results expressed per 100,000 population. EAPC was calculated by fitting a linear regression model to the natural logarithm of the ASRs, expressed as [ln (ASR) = α + β*(calendar year) + ϵ], and derived using the formula 100 × (exp (β) −1) along with its 95% confidence interval (CI) (28, 29). A statistically significant increasing trend was observed when both the EAPC estimate and the lower boundary of its 95% CI were greater than 0, whereas a significant decreasing trend was observed when both the EAPC estimate and the upper boundary of its 95% CI were less than 0. If the 95% CI included 0, the trend was considered stable (30). Future trends were projected using a Bayesian Age-Period-Cohort (BAPC) model, implemented with the ‘INLA’ and ‘BAPC’ packages in R, forecasting the incidence of EC through 2050.

All statistical analyses and visualizations were conducted in R (version 4.2.3) and JD_GBDR (V2.22, Jingding Medical Technology Co., Ltd.), with results presented as 95% uncertainty intervals (UIs). For trend analysis, p-values < 0.05 were considered statistically significant.

## 3 Results

### 3.1 Global level

In 2021, There were an estimated 576,529 cases (95% uncertainty interval [UI]: 509,492 to 645,648) of EC worldwide, with an age-standardized rate of 6.65 (95% UI: 5.88 to 7.45) per 100,000, which decreased by 24.87% (95% UI: 12.77 to 35.88) between 1990 and 2021 (**Supplementary Table 1**). Globally, EC accounted for 538,601 deaths (95% UI: 475,943 to 603,405) in 2021, with an age-standardized death rate of 6.25 (95% UI: 5.53 to 7.00) per 100,000, which decreased significantly by 30.67% (95% UI: 19.56 to 40.63) during the period 1990-2021. Additionally, EC was responsible for 12,999,264 DALYs (95% UI: 11,522,861 to 14,605,268) in 2021, with an age-standardized rate of 148.56 (95% UI: 131.71 to 166.82) per 100 000 which significantly decreased by 1.73% (95% UI: 1.59 to 1.88).

### 3.2 Regional level

From 1990 to 2021, the number of incident cases and deaths due to EC increased. Incident cases rose from 354,730 (95% UI: 317,512 to 388,914) in 1990 to 576,529 (95% UI: 509,492 to 645,648) in 2021, and deaths increased from 356,263 (95% UI: 319,363 to 390,154) to 538,602 (95% UI: 475,944 to 603,406). However, during this period, the contributions of the individual GBD regions differed. In 2021, East Asia accounted for nearly two-thirds of all incident and death cases of EC.

**Figure 1:**
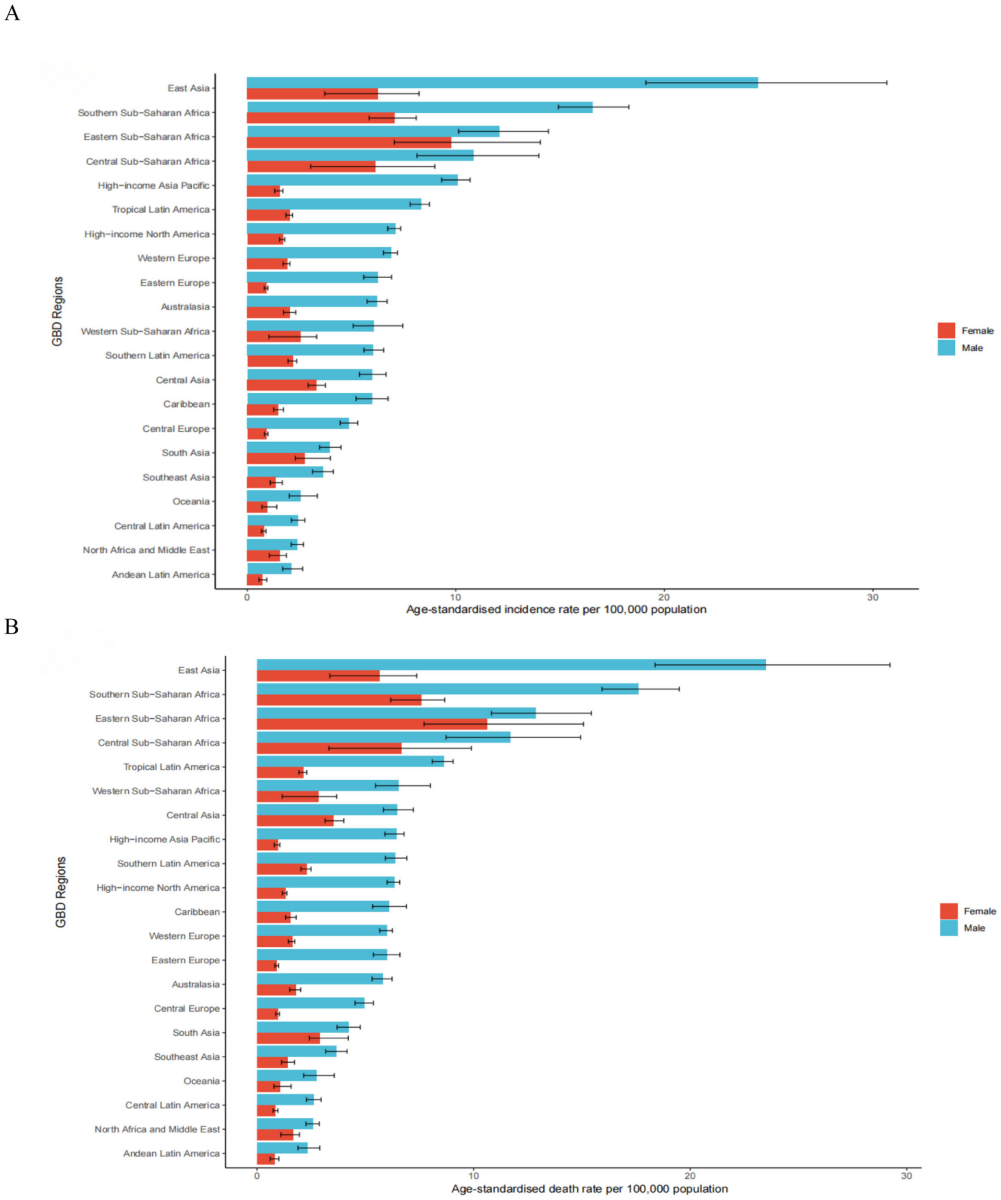
The age-standardised incidence **(A)** and death **(B)** rates of esophageal cancer per 100000 population in 2021 for the 21 Global Burden of Disease regions, by sex.

In 2021, the highest age-standardized incidence rates of esophageal cancer per 100,000 population were observed in Central Sub-Saharan Africa (8.26; 95% UI: 6.03 to 10.61), High-income Asia Pacific (5.49; 95% UI: 5.00 to 5.80) and Tropical Latin America (4.91; 95% UI: 4.64 to 5.11). In contrast, the lowest rates were in Andean Latin America (1.38; 95% UI: 1.14 to 1.70), Central Latin America (1.54; 95% UI: 1.37 to 1.73) and Oceania (1.81; 95% UI: 1.43 to 2.28) (**Supplementary Table 1**). Similarly, the highest age-standardized death rates per 100,000 were in Central Sub-Saharan Africa (8.89; 95% UI: 6.44 to 11.50), Tropical Latin America (5.07; 95% UI: 4.78 to 5.29) and Central Asia (4.74; 95% UI: 4.28 to 5.25), whereas the lowest death rates were in Andean Latin America (1.51; 95% UI: 1.24 to 1.85), Central Latin America (1.65; 95% UI: 1.47 to 1.85) and Oceania (1.95; 95% UI: 1.54 to 2.45) (**Supplementary Table 2**). Regional age-standardized incidence and death estimates per 100,000 for all GBD regions, stratified by sex, are presented in **Figures 1A, B**.

Between 1990 and 2021, the incident cases showed a downward trend in Central Asia, decreasing by 65.36% (95% UI: -68.89% to -61.33%) (**Supplementary Table 1**). A decreasing trend of ASIR was detected in 19 regions, with the largest decrease in Central Asia [(estimated annual percentage change) EAPC: -3.49; 95% CI: -3.67 to -3.31] (**Supplementary Table 1**). The ASIR remained stable in Caribbean (EAPC: 0.00, 95% CI: -0.14 to 0.15) (**Supplementary Table 1**). In 2021, East Asia had the highest ASDR at 13.91 per 100,000 (95% UI: 11.23 to 16.84) (**Supplementary Table 2**). Between 1990 and 2021, only in Western Sub-Saharan Africa did the number of death increase, rising by 60.06% (95% UI: 28.97% to 94.95%) (**Supplementary Table 2**). The ASDR decreased in most areas, with the most pronounced decline in Central Asia (EAPC: -3.49; 95% CI: -3.67 to -3.31) (**Supplementary Table 2**). An increasing trend occurred in Western Sub-Saharan Africa (EAPC: 2.09; 95% CI: 1.87 to 2.31) (**Supplementary Table 2**). East Asia also had the highest age-standardized DALYs rate due to esophageal cancer in 2021, at 313.94 per 100,000 (95% UI: 252.18 to 387.12) (**Supplementary Table 3**). Increasing trends of DALYs were observed in Western Sub-Saharan Africa, with an increase of 54.75% (95% UI: 23.32% to 93.17%) (**Supplementary Table 3**). A notable decline was observed in the age-standardized DALYs rate of Central Asia, with an EAPC of -3.63 (95% CI: -3.80 to -3.45) during the study period (**Supplementary Table 3**).

### 3.3 National level

**Figure 2:**
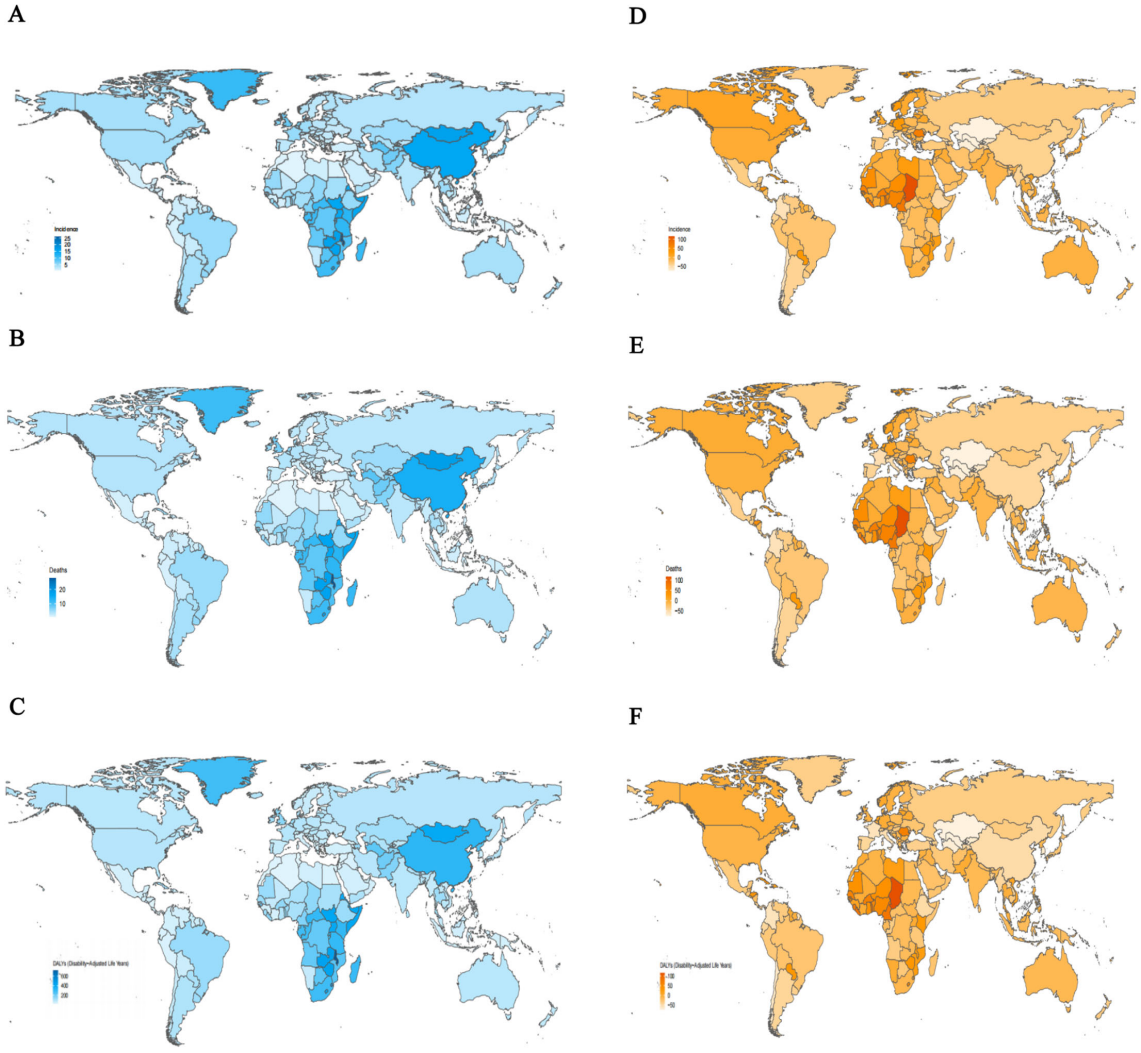
The age-standardized rates and percentage change in age-standardized rates of esophageal cancer for 204 countries and territories. **(A)** The age-standardized incidence rates of esophageal cancer, 2021. **(B)** The age-standardized mortality rates of esophageal cancer, 2021. **(C)** The age-standardized DALYs rates of esophageal cancer, 2021. **(D)** The percentage change in age-standardized incidence rates of esophageal cancer, 1990-2021. **(E)** The percentage change in age-standardized mortality rates of esophageal cancer, 1990-2021. **(F)** The percentage change in age-standardized DALYs rates of esophageal cancer, 1990-2021. DALYs, disability-adjusted life-year.

Among 204 countries and territories, the three highest ASIR of esophageal cancer in 2021 were observed in the Republic of Malawi (26.06; 95% UI: 21.02 to 32.46), the Kingdom of Eswatini (16.68; 95% UI: 11.80 to 22.42), and Mongolia (16.25; 95% UI: 13.15 to 19.45). In contrast, the lowest rates were reported in the Republic of Tunisia (0.67; 95% UI: 0.46 to 0.93), the People’s Democratic Republic of Algeria (0.70; 95% UI: 0.55 to 0.87), and the Republic of Nicaragua (0.82; 95% UI: 0.66 to 1.02) (**Supplementary Table 4**; **Figure 2A**). Similar patterns were observed for ASDR, with the Republic of Malawi (27.77; 95% UI: 22.45 to 34.72), Mongolia (17.98; 95% UI: 14.50 to 21.49), and the Kingdom of Eswatini (17.47; 95% UI: 12.43 to 23.38) had the highest death rates, whereas the Republic of Tunisia (0.69; 95% UI: 0.48 to 0.96), the People’s Democratic Republic of Algeria (0.75; 95% UI: 0.59 to 0.94), and the Republic of San Marino (0.85; 95% UI: 0.53 to 1.25) had the lowest (**Supplementary Table 5**; **Figure 2B**). In terms of age-standardized DALYs rates in 2021, the Republic of Malawi (715.28; 95% UI: 572.77 to 904.41), the Kingdom of Eswatini (478.85; 95% UI: 332.56 to 665.21), and the Kingdom of Lesotho (450.01; 95% UI: 327.62 to 588.49) ranked highest. Conversely, the lowest DALYs rates were found in the Republic of Tunisia (15.83; 95% UI: 10.75 to 22.28), the People’s Democratic Republic of Algeria (16.36; 95% UI: 12.54 to 20.52), and the State of Kuwait (19.70; 95% UI: 15.89 to 24.31) (**Supplementary Table 6**; **Figure 2C**). Between 1990 and 2021, the percentage change in ASIR of esophageal cancer varied substantially across countries and territories, Significant increases were observed in the Republic of Chad (115.15%; 95% UI: 59.66% to 202.26%), the Democratic Republic of Sao Tome and Principe (99.46%; 95% UI: 56.83% to 164.12%), and the Togolese Republic (93.51%; 95% UI: 42.77% to 171.00%) experiencing the most significant growth. In contrast, marked declines occurred in the Republic of Kazakhstan (-75.17%; 95% UI: -78.81% to -70.95%), the Republic of Uzbekistan (-73.83%; 95% UI: -78.40% to -68.94%), and Turkmenistan (-71.49%; 95% UI: -78.36% to -63.42%) had the sharpest decline (**Supplementary Table 4**; **Figure 2D**). For ASDR, the largest increase was noted in Republic of the Chad (114.76%; 95% UI: 59.29% to 196.01%), followed by the Democratic Republic of Sao Tome and Principe (97.93%; 95% UI: 56.18% to 158.94%) and the Togolese Republic (92.53%; 95% UI: 41.16% to 168.54%). The most significant decreases were observed in the Republic of Kazakhstan (-75.29%; 95% UI: -78.89% to -71.06%), the Republic of Uzbekistan (-73.66%; 95% UI: -78.20% to -68.69%), and Turkmenistan (-71.57%; 95% UI: -78.12% to -63.77%) (**Supplementary Table 5**; **Figure 2E**). Regarding age-standardized DALYs rates, the Republic of Chad showed the most substantial increase (115.03%; 95% UI: 57.04% to 200.88%), followed by the Democratic Republic of Sao Tome and Principe (96.13%; 95% UI: 50.92% to 164.72%), and the Togolese Republic (93.71%; 95% UI: 38.11% to 171.40%). In contrast, the largest decreases were seen in the Republic of Kazakhstan (-76.16%; 95% UI: -79.60% to -72.17%), the Republic of Uzbekistan (-74.62%; 95% UI: -79.31% to -69.54%), and Turkmenistan (-71.83%; 95% UI: -78.63% to -63.64%) showed the largest downward trends (**Supplementary Table 6**; **Figure 2F**).

### 3.4 Age and sex patterns

**Figure 3:**
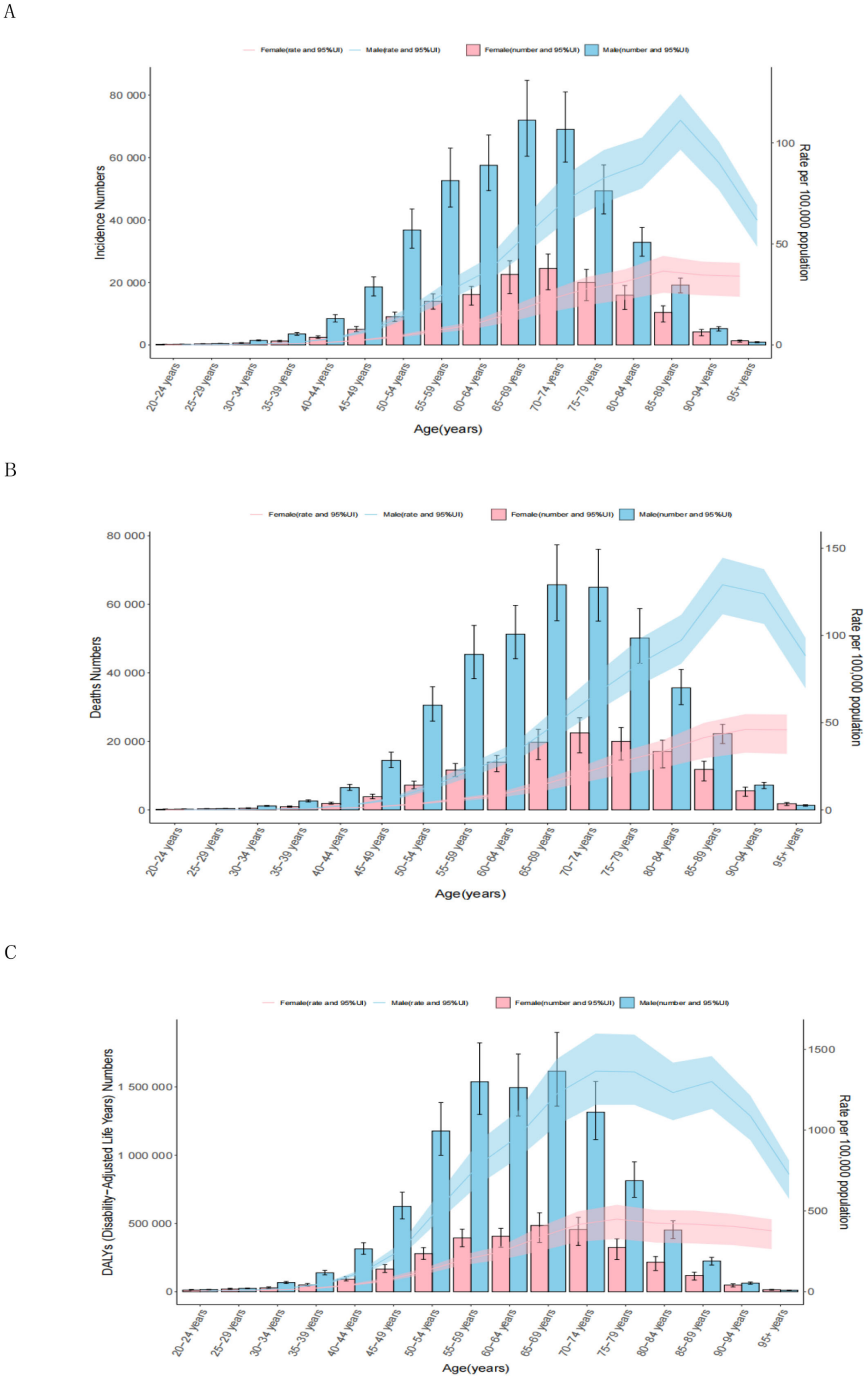
Age patterns by sex of the total number and age-standardized rates of incidence **(A)**, deaths **(B)**, and DALYs **(C)** of esophageal cancer in 2021. The box plots indicate numbers and line plots indicate rates per 100,000 population. Dashed lines indicate 95% upper and lower uncertainty intervals. DALYs, disability-adjusted life-year.

Globally, the ASRs were higher in males than females across all age groups (**Figure 3**). As age increased, the ASIRs gradually rose, with males peaking at 111.24 (95% CI: 96.63 to 124.19) in the 85–89 age group and females peaking at 36.60 (95% CI: 25.86 to 44.06) in the same group. Subsequently, the ASIRs declined in older age groups. The highest number of incident cases was observed in the 65–69 age group for males and 70–74 age group for females (**Figure 3A**; **Supplementary Table 7**). The ASDRs followed a similar trend, with peak values in the 85–89 age group for males (129.07; 95% CI: 112.14 to 144.60) and in the 90–94 age group for females (46.13; 95% CI: 32.78 to 54.90). The highest number of deaths cases was peaked at 65–69 for males and 70–74 for females (**Figure 3B**; **Supplementary Table 8**). The DALYs reached their highest levels at 1364.52 (95% CI: 1156.10–1597.54) for males in the 70–74 age group and 449.23 (95% CI: 326.50–537.64) for females in the 75–79 age group. The number of DALYs peaked at ages 65–69 for both in males and females (**Figure 3C**; **Supplementary Table 9**). The DALYs were predominantly composed of YLLs, with the YLL rate peaking in the 70–74 age group for males and in the 75–79 age group for females. The number of YLLs and YLDs were highest in the 65–69 age group (**Supplementary Figures 1A, B**; **Supplementary Tables 10**, **11**).

### 3.5 Burden of esophageal cancer by sociodemographic index

**Figure 4:**
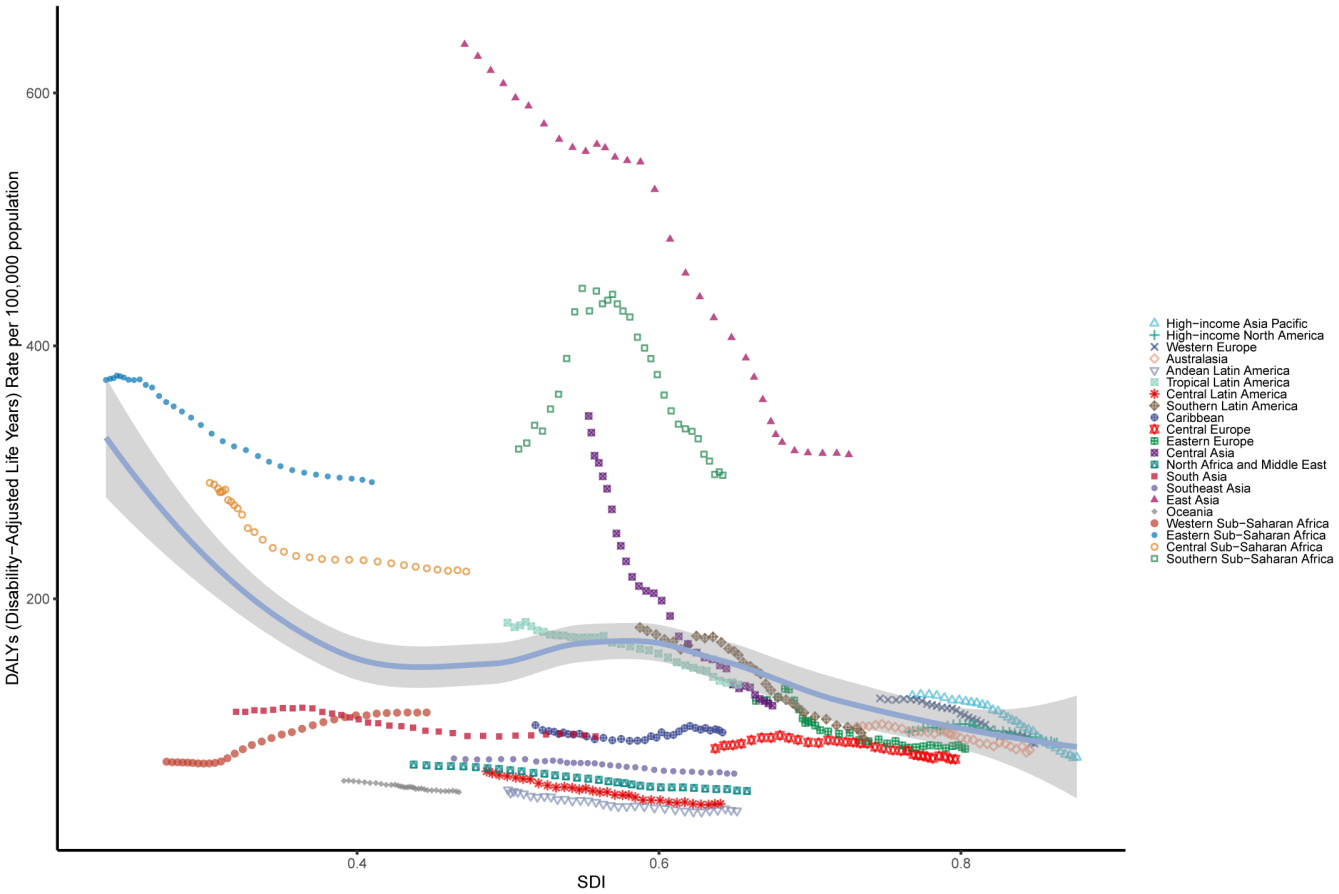
The age-standardised rates of EC DALYs globally and for 21 GBD regions by SDI from 1990 to 2021. The expected age-standardised rates in 2021 based solely on SDI were represented by the black line. For each region, points from left to right depict estimates from each year from 1990 to 2021. DALYs, disability-adjusted life-years; GBD, Global Burden of Disease; EC, esophageal cancer; SDI, Socio demographic Index.

**Figure 4** illustrates trends in age-standardized DALYs rates across SDI regions from 1990 to 2021, highlighting the relationship between socioeconomic development and the burden of EC. The patterns follow a nonlinear trajectory, with DALYs rates peaking at an SDI value of approximately 0.55 before declining as SDI values increased. High-SDI regions closely adhered to expected patterns, showing steady declines over time. In contrast, Middle-SDI regions exhibited considerable variability (**Figure 4**; **Supplementary Table 12**). Most regions experienced a decrease in age-standardized DALYs rates during the study period, with East Asia demonstrating the largest reduction. In Southern Sub-Saharan Africa, DALYs rates initially rose sharply, then declined significantly, and continued to decrease as SDI values increased. These trends aligned with those observed for age-standardized incidence and death rates across SDI regions (**Supplementary Figures 2A, B**; **Supplementary Tables 13**, **14**).

### 3.6 Attributable risks

**Figure 5:**
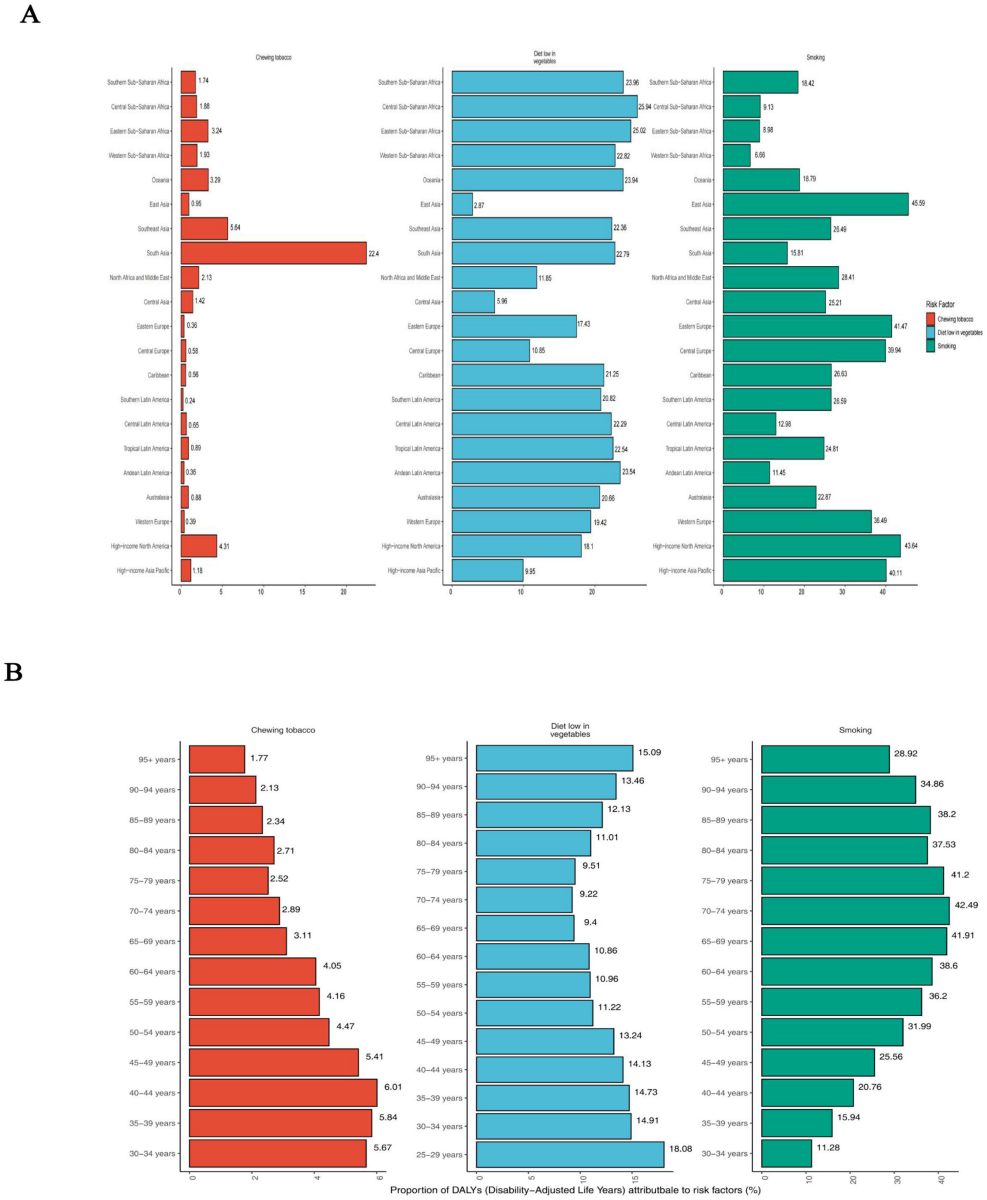
Percentage of age-standardised DALYs due to esophageal cancer attributable to risk factors, 2021. **(A)** Distribution for 21 Global Burden of Disease regions. **(B)** Distribution by age for both sexes. DALYs, disability-adjusted life year.

Globally, for both sexes, a substantial proportion of DALYs were attributable to the three risk factors for which GBD estimates were available: 36.45% (95% UI: 29.49% to 42.70%) due to smoking, 10.78% (95% UI: -2.38% to 21.95%) due to a diet low in vegetables, and 3.68% (95% UI: 2.52% to 4.91%) due to chewing tobacco. The impact of these risk factors varied among regions. Chewing tobacco had the highest impact in Southeast Asia (5.64%; 95% UI: 3.92% to 7.50%) and the lowest in the Southern Latin America (0.24%; 95% UI: 0.15% to 0.35%) (**Figure 5A**). A diet low in vegetables had the highest impact in Central Sub-Saharan Africa (25.94%; 95% UI: -6.26% to 49.85%) and the lowest in the East Asia (2.87%; 95% UI: -0.52% to 7.52%) (**Figure 5A**). Smoking had the highest impact in East Asia (45.59%; 95% UI: 37.83% to 52.77%) and the lowest in the Western Sub-Saharan Africa (6.66%; 95% UI: 4.91% to 8.75%) (**Figure 5A**). The global patterns of attributable risk differed by age group. The percentages of attributable DALYs were highest for chewing tobacco in the 40–44 year age group, for a diet low in vegetables in the 25–29 year age group, and for smoking in the 70–74 year age group. Conversely, they were lowest for chewing tobacco in the 95 years and older group, for a diet low in vegetables in the 70–74 year age group, and for smoking in the 30–34 year age group (**Figure 5B**).

### 3.7 The correlation between SDI and esophageal cancer’s incidence and mortality

**Figure 6:**
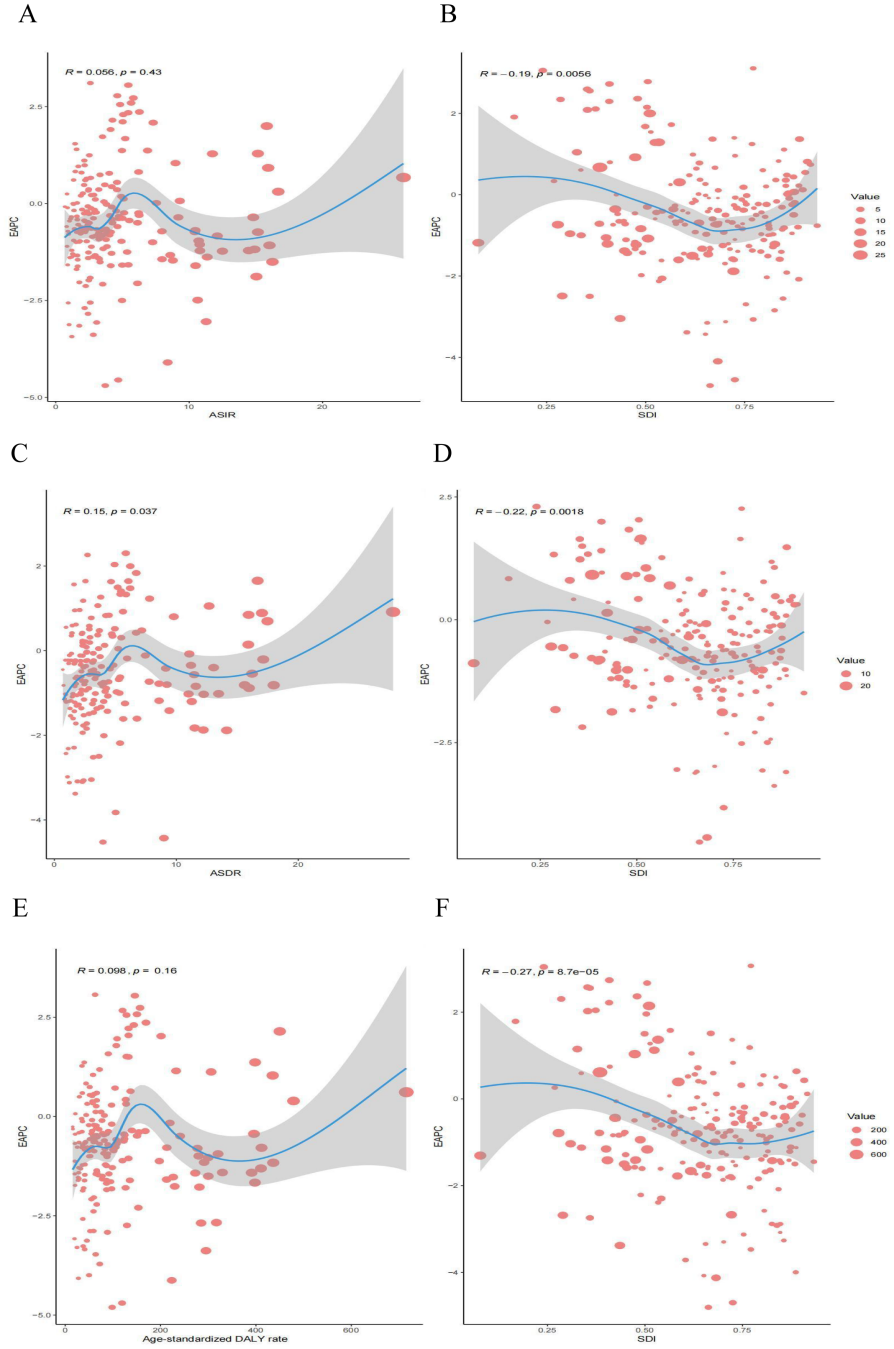
Correlation between EAPC and esophageal cancer ASR [incidence **(A)**, deaths **(C)**, and DALYs **(E)**] and SDI [incidence **(B)**, deaths **(D)**, and DALYs **(F)**] in 2021. EAPC, estimated annual percentage change; ASR, age-standardized rate; DALYs, disability-adjusted life years; SDI, socio demographic index.

By calculating Pearson’s correlation coefficients, we assessed the relationships between EAPC values and SDI in 2021 across 204 countries for ASIR, ASDR, and age-standardized DALYs rates. We found that EAPC was negatively correlated with SDI for ASIR (R = -0.19; P = 0.0056), ASDR (R = -0.22; P = 0.0018), and age-standardized DALYs rates (R = -0.27; P < 0.001) (**Figures 6B, D, F**). Additionally, EAPC was positively correlated with ASDR (R = 0.15; P = 0.037) (**Figure 6C**). No significant correlation was observed between EAPC and ASIR (R = 0.056; P = 0.43) or between EAPC and DALYs (R = 0.098; P = 0.16), these results are presented in the **Figures 6A, E**.

### 3.8 Future forecasts of global burden of disease in esophageal cancer

**Figure 7:**
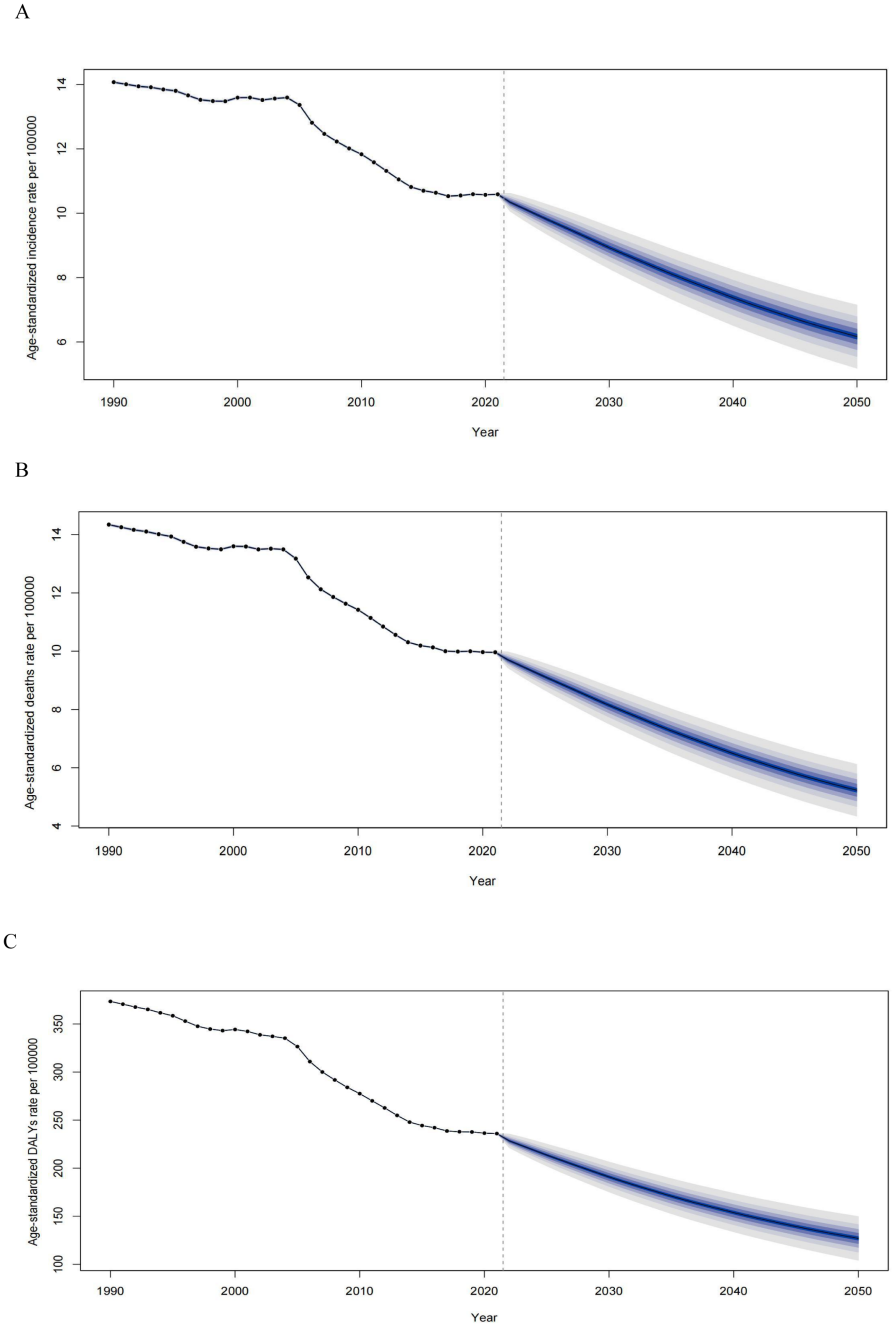
Observed and predicted age-standardized rates of incidence **(A)**, deaths **(B)**, and DALYs **(C)** of esophageal cancer globally from 1990 to 2050, using the BAPC model. The blue region shows the upper and lower limits of the 95% UIs. ASIR, age-standardized incidence rate; ASDR, age-standardized death rate; DALYs, disability-adjusted life years; BAPC, Bayesian age-period-cohort model; UIs, uncertainty intervals.

The projected trends in age-standardized rates for EC incidence, mortality, and DALYs from 1990 to 2050 are illustrated (**Figures 7A–C**). The ASIR for EC has steadily declined from 1990 to 2021 and is expected to continue decreasing, albeit at a slower pace, through 2050 (**Figure 7A**). By 2050, the ASIR is estimated to reach 6.17 per 100,000, representing a 4.42% reduction compared to 2021. Similarly, the ASDR has significantly decreased over recent decades and is projected to decline further by 2050, with an estimated ASDR of 5.23 per 100,000, representing a 4.73% decrease compared to 2021 (**Figure 7B**). Trends in DALYs also show a consistent reduction from 1990 to 2021, with a continued decline anticipated through 2050 (**Figure 7C**).

## 4 Discussion

This study provides a comprehensive analysis of the global burden of esophageal cancer from 1990 to 2021, highlighting substantial disparities in incidence, mortality, and DALYs across different regions, age groups, and socioeconomic strata. Globally, there was a significant decline in ASIR and ASDR during the study period, indicate that medical interventions and preventive measures have made substantial progress in controlling the burden of EC. However, despite these positive trends, the absolute number of new cases and deaths has continued to rise. This paradox can be largely attributed to demographic changes, particularly population growth and aging, which have contributed to a higher burden of disease in absolute terms (31). Despite advancements in screening and treatment techniques that have reduced the relative risk of cancer in many high-income countries, the growing elderly population and the increased availability of diagnostic technologies have resulted in the detection of more cases, thereby diminishing some of the gains achieved through these improvements (32, 33).

As a malignant tumor of poor prognosis, esophageal cancer shows significantly regional differences in the world (34, 35). The prevalence of EC is high in East Asia, with China bearing a disproportionately large share of the global burden (36, 37). More than half of the world’ s new cases and esophageal cancer-related deaths occur in China, driven by the widespread impact of risk factors such as smoking and alcohol consumption (38, 39). Studies have demonstrated that lifestyle factors, especially high tobacco use, contribute significantly to ESCC, the dominant subtype in the region (40). Despite China’s progress in national screening programs, large disparities in healthcare access between rural and urban areas contribute to high mortality and DALYs rates, particularly in rural populations, where early detection is challenging (41, 42). Public health interventions, such as tobacco control campaigns, are urgently needed to address this ongoing crisis.

In Sub-Saharan Africa, the burden is similarly disproportionate, with Central Sub-Saharan Africa reporting the highest incidence and mortality rates on the continent. In Africa, the area with a high incidence of esophageal cancer is referred to as the “African esophageal cancer corridor”, which runs from Ethiopia and Kenya to South Africa (43, 44). This region also recorded the highest DALYs rates globally in 2021. The region’s burden is compounded by healthcare system limitations, leading to late diagnoses and poor treatment outcomes (45). These findings are consistent with previous research showing that access to healthcare is a critical determinant of cancer outcomes in low-income countries (46). In contrast, regions with more robust healthcare systems, such as Oceania and parts of Latin America, show significantly lower EC burdens. For instance, Central Latin America benefits from earlier diagnoses and better treatment outcomes, largely due to more accessible healthcare systems. These regional disparities emphasize the role of healthcare infrastructure in mitigating esophageal cancer burden.

Nationally, the variations in esophageal cancer burden are striking. China continues to exhibit high esophageal cancer incidence and mortality rates, esophageal cancer is the fourth most common cancer type in China and nearly half of all newly diagnosed cases of esophageal cancer and associated deaths worldwide occur in China (47, 48). ESCC is highly prevalent in China, which accounts for more than 90% of the overall esophageal carcinoma population (49). This highlights the urgent need for widespread public health interventions targeting high-risk populations, particularly in rural areas. Countries such as Malawi, Eswatini, and Mongolia, which exhibit the highest rates, are likely influenced by environmental exposures, tobacco and alcohol consumption, and limited access to early detection and treatment (50). This contrasts sharply with countries like Tunisia and Algeria, where lower rates may be attributed to improved healthcare infrastructure and public health initiatives. Our results align with previous studies, which have similarly identified sub-Saharan Africa and parts of Asia as high-burden regions for esophageal cancer, while showing that certain regions, particularly in Central Asia, have successfully reduced their incidence through enhanced screening and prevention efforts (51, 52). Notably, the increasing rates in countries like Chad and Sao Tome underscore the need for urgent public health interventions to curb this trend. These findings emphasize the importance of tailored strategies addressing local risk factors and healthcare limitations, consistent with global cancer control frameworks aimed at reducing disparities in cancer outcomes.

The marked differences in esophageal cancer incidence, mortality, and DALYs rates by age and sex in 2021 underscore the profound impact of demographic factors on disease burden. Our findings reveal that males consistently exhibit higher rates across all age groups, peaking in the 85–89 range, which reflects not only greater exposure to risk factors such as smoking and alcohol but also potential genetic and hormonal influences (17, 22). This age-specific trend suggests that current prevention strategies may be insufficient for older populations, particularly in males, and underscores the need for tailored interventions that address the unique risk profiles of aging demographics. Moreover, the substantial burden of DALYs driven predominantly by YLLs points to the aggressive nature of the disease in later stages, emphasizing the critical need for earlier detection and more effective management in elderly populations. The convergence of peak incidences and DALYs at earlier ages (65–69 years) for both sexes suggests an opportunity for intervention before the disease reaches its most lethal stages, aligning with global efforts to mitigate cancer mortality through enhanced screening and early intervention programs (53, 54). These insights advocate for a shift toward age-specific and sex-specific strategies in combating esophageal cancer, which could significantly reduce the disease burden globally.

The observed nonlinear relationship between the SDI and the age-standardized DALYs rates for esophageal cancer highlights a critical intersection between socioeconomic development and disease burden. Middle–SDI regions show disproportionately high DALYs rates, with certain countries such as Malawi, Eswatini, and Lesotho significantly exceeding expected levels. This suggests that while socioeconomic progress may improve overall health outcomes, it does not automatically mitigate cancer burdens without targeted interventions. The wide variation in outcomes across similar SDI regions underscores the importance of addressing region-specific risk factors, such as tobacco use and dietary habits, which remain prevalent despite rising SDI levels. In contrast, countries like Tunisia and Kuwait, with lower-than-expected DALYs rates, reflect the success of robust cancer screening and healthcare access in reducing disease impact (55). These findings suggest that global cancer control efforts must go beyond socioeconomic improvements and prioritize public health initiatives that target modifiable risk factors and improve access to early detection and treatment in middle-SDI regions, where the burden of disease remains high.

Attributable risk factors such as smoking, poor diet, and chewing tobacco remain the predominant contributors to the global burden of esophageal cancer. Smoking, particularly in East Asia, accounts for nearly half of the attributable DALYs, underscoring its outsized impact in this region compared to others like Sub-Saharan Africa. This mirrors prior studies linking smoking prevalence with esophageal cancer incidence and highlights the importance of strengthening tobacco control measures (56). Similarly, the burden associated with a diet low in vegetables in Central Sub-Saharan Africa further emphasizes the need for nutritional interventions, contrasting with the success of dietary improvements in high-income countries that have significantly reduced esophageal cancer risks (57). The differing impact of risk factors across age groups, with younger populations more affected by diet and older groups by smoking, suggests a need for age-specific prevention strategies. These findings suggest that to effectively reduce the global esophageal cancer burden, public health policies should prioritize targeted risk reduction strategies-such as tobacco cessation programs and dietary interventions-tailored to the specific needs of high-risk regions and age groups, with an emphasis on early intervention and sustained education on lifestyle modifications.

The correlation between the SDI and the global burden of EC reveals complex patterns of inequality. The negative association between SDI and the EAPC of both ASIR and ASDR, combined with the absence of a correlation with DALYs rates, underscores the crucial role socioeconomic development plays in reducing EC incidence and mortality. Higher SDI regions benefit from greater access to healthcare, more effective prevention programs, and earlier detection, explaining the steeper declines in ASIR and ASDR observed in these areas (58). In contrast, the persistent burden in low-SDI regions highlights the significant disparities in healthcare infrastructure, screening availability, and public health interventions (59).

Future projections emphasize that, despite declining age-standardized rates, the absolute number of EC cases and deaths is expected to rise by 2050 due to population growth and aging. While reductions in ASIR and ASDR are promising, the anticipated surge in cases highlights the critical need for sustained and targeted prevention efforts. These findings echo similar trends seen in other non-communicable diseases, where the aging population is expected to counteract improvements in disease-specific rates. It is crucial to focus prevention strategies on regions with lower SDI values, where modifiable risk factors, such as smoking and poor diet, remain prevalent.

This study has several limitations. First, the reliance on GBD estimates may introduce uncertainties, particularly in low- and middle-income regions where cancer registries are underdeveloped or data quality is variable. These discrepancies could result in over-or underestimations of the burden in specific regions. Moreover, while we accounted for major risk factors, other emerging factors, such as environmental pollutants and genetic predispositions, were not fully explored due to data limitations. Another potential limitation is the forecasting model, which, while robust, may not adequately capture future shifts in healthcare infrastructure, policy interventions, or unforeseen socio-political events that could alter esophageal cancer trends in the coming decades. Additionally, the aggregated nature of the data prevents detailed subgroup analyses that could yield more nuanced insights, particularly in understanding variations within specific populations or subnational regions.

Future efforts should prioritize the integration of molecular and genetic research with public health strategies to identify high-risk populations earlier and offer personalized interventions. Advances in early detection, such as liquid biopsy technologies and non-invasive screening tools, hold significant promise in mitigating the disease burden, especially in resource-limited settings (60, 61). Furthermore, addressing health disparities through targeted public health policies, such as increasing access to cancer screening and implementing tailored prevention programs in high-burden regions, remains crucial. As the global population ages, it is essential to explore novel interventions that cater to elderly populations, focusing on prevention, early detection, and optimized treatment strategies for this vulnerable group. In the context of an evolving global healthcare landscape, collaboration across international borders, with a focus on knowledge-sharing and resource optimization, will be vital to reducing the global burden of esophageal cancer.

## Data Availability

The original contributions presented in the study are included in the article/**Supplementary Material**. Further inquiries can be directed to the corresponding author.
