# Supplementary material for: Burden of esophageal cancer in global, regional and national regions from 1990 to 2021 and its projection until 2050: results from the GBD study 2021
Source: Front Oncol. 2025 Jan 20;14:1518567. doi: 10.3389/fonc.2024.1518567 (PMC11788179; doi:10.3389/fonc.2024.1518567)
Supplement: Supplementary file 1 [file Table1.docx]

Supplementary Material

# Supplementary Tables

**Supplementary Table 1 Age-standardised Incidence of Esophageal Cancer Between 1990 and 2021 at the Global and Regional Level**

|  | **1990** | | **2021** | | **Percentage change 1990-2021**  **ASIR(95%UI)** | **EAPC of**  **ASIR**  **(95% CI)** |
| --- | --- | --- | --- | --- | --- | --- |
|  | **Incidence cases**  **(95%UI)** | **ASIR per 10^5^**  **(95%UI)** | **Incidence cases**  **(95%UI)** | **ASIR per 10^5^**  **(95%UI)** |  |  |
| **Global** | 354730.82  (317512.39-388914.46) | 8.86(7.96-9.69) | 576529.28  (509492.07-645648.46) | 6.65(5.88-7.45) | -24.87(-35.88--12.77) | -1.12(-1.26--0.99) |
|  |  |  |  |  |  |  |
| **SDI** |  |  |  |  |  |  |
| High | 58595.63(56146.52-60081.76) | 5.36(5.14-5.49) | 102509.51(95223.90-107347.84) | 4.94(4.63-5.16) | -7.76(-11.38--4.30) | -0.34(-0.51--0.18) |
| High-middle | 112266.70(98612.72-125910.53) | 11.17(9.85-12.49) | 176767.70(145141.06-214115.58) | 8.84(7.26-10.70) | -20.85(-36.75--1.70) | -0.97(-1.15--0.80) |
| Middle SDI | 143087.61(120407.77-165384.77) | 13.68(11.49-15.77) | 216950.81(182211.66-258446.04) | 8.10(6.78-9.62) | -40.77(-52.61--26.36) | -1.95(-2.11--1.80) |
| Low-middle | 25326.36(22893.39-29038.10) | 4.10(3.68-4.70) | 52104.25(47165.62-59925.96) | 3.59(3.24-4.15) | -12.30(-20.96--1.21) | -0.50(-0.55--0.45) |
| Low SDI | 15304.78(12751.23-17274.85) | 6.69(5.58-7.51) | 27960.08(23834.45-32184.41) | 5.49(4.70-6.32) | -17.90(-31.12--3.11) | -0.80(-0.87--0.72) |
| **Regions** |  |  |  |  |  |  |
| Andean Latin America | 385.52(339.96-439.47) | 1.96(1.73-2.23) | 801.52(656.28-986.85) | 1.38(1.14-1.70) | -29.54(-43.69--10.29) | -1.20(-1.32--1.07) |
| Australasia | 1054.81(1000.41-1113.81) | 4.47(4.23-4.72) | 2202.08(1983.17-2360.60) | 4.05(3.68-4.33) | -9.39(-16.23--2.48) | -0.40(-0.50--0.30) |
| Caribbean | 1001.05(943.85-1062.55) | 3.91(3.69-4.14) | 1951.51(1713.39-2207.34) | 3.60(3.17-4.08) | -7.90(-18.55-3.70) | 0.00(-0.14-0.15) |
| Central Asia | 5950.87(5673.20-6219.06) | 12.77(12.15-13.35) | 3573.46(3191.08-3973.51) | 4.42(3.98-4.90) | -65.36(-68.89--61.33) | -3.49(-3.67--3.31) |
| Central Europe | 4318.84(4158.10-4496.01) | 2.89(2.77-3.00) | 5758.55(5283.60-6213.97) | 2.73(2.50-2.95) | -5.54(-13.58-2.33) | -0.43(-0.56--0.30) |
| Central Latin America | 2033.21(1961.74-2080.42) | 2.57(2.46-2.63) | 3807.17(3397.96-4285.04) | 1.54(1.37-1.73) | -40.07(-46.15--32.92) | -1.78(-1.86--1.70) |
| Central Sub-Saharan Africa | 2402.49(1778.66-3023.88) | 10.59(7.92-13.19) | 4536.33(3324.92-5881.13) | 8.26(6.03-10.61) | -22.00(-41.10-2.26) | -0.94(-1.04--0.84) |
| East Asia | 210687.00(175692.13-244703.44) | 24.23(20.24-28.01) | 327705.98(263648.30-401882.11) | 14.83(11.94-18.09) | -38.79(-52.83--21.53) | -1.85(-2.07--1.63) |
| Eastern Europe | 12395.13(12109.91-12644.71) | 4.36(4.25-4.44) | 10710.19(9692.24-11617.78) | 3.09(2.79-3.35) | -29.19(-35.82--22.78) | -1.33(-1.55--1.10) |
| Eastern Sub-Saharan Africa | 10170.23(8337.96-11728.04) | 13.56(11.19-15.62) | 18379.40(15328.43-22109.54) | 10.93(9.14-13.09) | -19.42(-34.39--1.54) | -0.88(-0.96--0.81) |
| High-income Asia Pacific | 13280.43(12676.15-13830.39) | 6.53(6.23-6.81) | 25547.08(22819.58-27075.79) | 5.49(5.00-5.80) | -15.96(-20.80--11.45) | -0.64(-0.85--0.44) |
| High-income North America | 14117.06(13443.24-14467.72) | 4.12(3.93-4.21) | 27331.14(25620.16-28408.91) | 4.20(3.96-4.36) | 2.11(-0.93-4.86) | -0.03(-0.20-0.14) |
| North Africa and Middle East | 4306.61(3436.39-4940.92) | 2.59(2.10-2.97) | 8684.27(7366.73-9766.19) | 1.99(1.71-2.22) | -23.21(-32.66--11.74) | -0.93(-0.98--0.87) |
| Oceania | 60.03(45.38-80.10) | 2.11(1.65-2.75) | 131.29(103.48-165.85) | 1.81(1.43-2.28) | -14.40(-38.42-9.37) | -0.51(-0.54--0.48) |
| South Asia | 23221.83(20619.84-28075.51) | 3.93(3.45-4.75) | 50081.33(44229.95-59870.00) | 3.36(2.95-4.03) | -14.50(-25.26--1.43) | -0.75(-0.86--0.64) |
| Southeast Asia | 6956.42(5837.69-8146.49) | 2.69(2.26-3.14) | 16164.15(13983.69-18580.25) | 2.42(2.11-2.76) | -9.93(-25.88-10.80) | -0.40(-0.44--0.36) |
| Southern Latin America | 3275.86(3131.43-3418.65) | 7.17(6.85-7.49) | 3432.15(3190.33-3666.98) | 3.89(3.62-4.15) | -45.85(-49.48--41.90) | -1.98(-2.20--1.76) |
| Southern Sub-Saharan Africa | 3099.79(2768.86-3571.63) | 11.23(9.98-13.05) | 6410.17(5853.48-7005.74) | 11.01(10.06-11.99) | -1.90(-16.04-11.83) | -0.43(-0.92-0.06) |
| Tropical Latin America | 6089.81(5844.51-6264.15) | 6.63(6.30-6.83) | 12766.94(12076.59-13279.75) | 4.91(4.64-5.11) | -25.94(-29.58--22.72) | -0.94(-1.03--0.86) |
| Western Europe | 27618.34(26586.79-28332.03) | 4.92(4.75-5.04) | 38417.26(35454.35-40217.13) | 4.26(4.00-4.44) | -13.48(-16.91--10.30) | -0.48(-0.57--0.39) |
| Western Sub-Saharan Africa | 2305.48(1865.30-2812.77) | 2.65(2.16-3.21) | 8137.29(6101.60-9760.01) | 4.22(3.15-5.02) | 59.32(28.28-95.38) | 2.07(1.85-2.29) |

**ASIR, age-standardized incidence rate; SDI, Socio-demographic index; CI, confidential interval; UI, uncertainty interval.**

**Supplementary Table 2 Age-standardised Deaths of Esophageal Cancer Between 1990 and 2021 at the Global and Regional Level**

|  | **1990** | | **2021** | | **Percentage change 1990-2021**  **ASDR(95%UI)** | **EAPC of**  **ASDR**  **(95% CI)** |
| --- | --- | --- | --- | --- | --- | --- |
|  | **Deaths cases**  **(95%UI)** | **ASDR per 10^5^**  **(95%UI)** | **Deaths cases**  **(95%UI)** | **ASDR per 10^5^**  **(95%UI)** |  |  |
| **Global** | 356263.42(319362.85-390154.25) | 9.02(8.11-9.87) | 538601.91(475943.98-603405.55) | 6.25(5.53-7.00) | -30.67(-40.63--19.56) | -1.41(-1.55--1.26) |
|  |  |  |  |  |  |  |
| **SDI** |  |  |  |  |  |  |
| High | 54240.21(51961.85-55653.13) | 4.93(4.73-5.06) | 85652.18(79160.34-89949.55) | 4.02(3.75-4.20) | -18.52(-21.82--15.28) | -0.78(-0.90--0.67) |
| High middle | 114331.62(100753.43-128040.03) | 11.52(10.19-12.87) | 162430.46(134261.52-195466.77) | 8.13(6.72-9.77) | -29.44(-43.33--13.07) | -1.38(-1.58--1.17) |
| Middle SDI | 145568.12(123678.42-168237.56) | 14.31(12.19-16.45) | 207633.66(174863.42-246497.96) | 7.91(6.65-9.34) | -44.71(-55.35--31.76) | -2.18(-2.35--2.01) |
| Low middle | 26141.02(23617.15-30034.98) | 4.36(3.92-5.02) | 53724.17(48512.78-61805.78) | 3.79(3.42-4.39) | -13.02(-21.64--1.93) | -0.53(-0.58--0.47) |
| Low SDI | 15827.87(13204.96-17869.09) | 7.15(5.97-8.01) | 28923.78(24610.71-33445.21) | 5.89(5.02-6.80) | -17.62(-30.86--2.96) | -0.78(-0.85--0.70) |
| **Regions** |  |  |  |  |  |  |
| Andean Latin America | 418.48(368.90-476.10) | 2.18(1.91-2.47) | 866.29(712.21-1063.12) | 1.51(1.24-1.85) | -30.69(-44.41--12.70) | -1.24(-1.36--1.13) |
| Australasia | 1020.95(967.32-1080.97) | 4.34(4.11-4.60) | 2049.87(1849.15-2203.54) | 3.68(3.33-3.94) | -15.17(-21.66--8.69) | -0.64(-0.72--0.56) |
| Caribbean | 1056.94(997.67-1121.31) | 4.18(3.95-4.43) | 1996.55(1755.47-2254.29) | 3.68(3.24-4.16) | -11.97(-22.40--0.85) | -0.15(-0.29--0.01) |
| Central Asia | 6279.50(5977.64-6567.92) | 13.68(13.00-14.33) | 3735.27(3341.50-4158.36) | 4.74(4.28-5.25) | -65.31(-68.89--61.20) | -3.49(-3.67--3.31) |
| Central Europe | 4481.41(4313.89-4659.19) | 3.02(2.90-3.13) | 5926.25(5440.86-6393.53) | 2.76(2.54-2.98) | -8.43(-15.94--1.03) | -0.53(-0.66--0.41) |
| Central Latin America | 2179.89(2100.87-2231.97) | 2.84(2.72-2.91) | 4028.78(3594.39-4523.94) | 1.65(1.47-1.85) | -42.00(-47.94--35.23) | -1.88(-1.96--1.80) |
| Central Sub-Saharan Africa | 2471.46(1822.56-3119.70) | 11.36(8.50-14.21) | 4653.32(3398.06-6068.93) | 8.89(6.44-11.50) | -21.75(-41.25-2.98) | -0.93(-1.03--0.83) |
| East Asia | 213973.30(179118.17-247918.74) | 25.43(21.28-29.31) | 302581.77(243362.52-368742.89) | 13.91(11.23-16.84) | -45.32(-57.67--30.15) | -2.23(-2.47--1.98) |
| Eastern Europe | 12469.71(12181.03-12701.32) | 4.41(4.30-4.49) | 10305.08(9378.25-11158.80) | 2.94(2.68-3.19) | -33.24(-39.28--27.50) | -1.53(-1.73--1.33) |
| Eastern Sub-Saharan Africa | 10522.61(8661.82-12124.37) | 14.52(11.97-16.70) | 19000.22(15876.09-22909.15) | 11.74(9.82-14.12) | -19.15(-33.86--1.25) | -0.86(-0.94--0.79) |
| High-income Asia Pacific | 10303.66(9798.19-10770.08) | 5.13(4.87-5.37) | 16914.20(15031.37-17974.17) | 3.42(3.10-3.62) | -33.29(-37.44--29.61) | -1.43(-1.53--1.34) |
| High-income North America | 12926.62(12268.10-13264.12) | 3.73(3.55-3.82) | 23959.83(22393.88-24951.66) | 3.62(3.40-3.76) | -3.02(-5.84--0.53) | -0.20(-0.34--0.05) |
| North Africa and Middle East | 4466.22(3584.95-5136.22) | 2.79(2.26-3.19) | 8845.88(7499.67-9958.91) | 2.11(1.81-2.36) | -24.31(-33.46--13.58) | -0.95(-1.00--0.91) |
| Oceania | 61.27(46.83-80.75) | 2.29(1.80-2.95) | 133.76(105.74-170.03) | 1.95(1.54-2.45) | -14.91(-38.18-8.53) | -0.53(-0.55--0.50) |
| South Asia | 23827.33(21092.33-28893.97) | 4.17(3.66-5.06) | 51542.45(45650.77-61687.54) | 3.54(3.12-4.26) | -15.09(-25.62--2.36) | -0.77(-0.88--0.66) |
| Southeast Asia | 7108.16(5964.00-8306.41) | 2.83(2.39-3.30) | 15829.52(13724.97-18154.18) | 2.44(2.13-2.78) | -13.87(-28.84-5.04) | -0.55(-0.60--0.51) |
| Southern Latin America | 3500.25(3341.94-3656.29) | 7.75(7.38-8.11) | 3627.09(3345.94-3878.83) | 4.07(3.77-4.35) | -47.48(-50.96--43.67) | -2.06(-2.28--1.85) |
| Southern Sub-Saharan Africa | 3188.92(2852.24-3705.36) | 11.89(10.59-13.90) | 6601.81(6025.75-7225.32) | 11.69(10.68-12.72) | -1.61(-15.99-12.12) | -0.41(-0.91-0.08) |
| Tropical Latin America | 6297.27(6040.65-6482.71) | 7.04(6.68-7.27) | 13112.97(12382.61-13660.55) | 5.07(4.78-5.29) | -27.94(-31.50--24.80) | -1.01(-1.10--0.93) |
| Western Europe | 27288.01(26179.52-28012.24) | 4.80(4.62-4.92) | 34397.48(31524.77-36115.04) | 3.65(3.41-3.81) | -24.00(-27.03--21.34) | -0.95(-1.02--0.87) |
| Western Sub-Saharan Africa | 2421.46(1964.16-2948.27) | 2.86(2.34-3.44) | 8493.54(6354.67-10192.08) | 4.58(3.43-5.43) | 60.06(28.97-94.95) | 2.09(1.87-2.31) |

**ASDR, age-standardized deaths rate; SDI, Socio-demographic index; CI, confidential interval; UI, uncertainty interval.**

**Supplementary Table 3 Age-standardised DALYs of Esophageal Cancer Between 1990 and 2021 at the Global and Regional Level**

|  | **1990** | | **2021** | | **Percentage change 1990-2021**  **DALYs (95%UI)** | **EAPC of**  **DALYs**  **(95% CI)** |
| --- | --- | --- | --- | --- | --- | --- |
|  | **DALYs cases**  **(95%UI)** | **DALYs per 10^5^**  **(95%UI)** | **DALYs cases**  **(95%UI)** | **DALYs per 10^5^**  **(95%UI)** |  |  |
| **Global** | 9753566.25(8719319.34-10739560.72) | 235.32(210.52-258.68) | 12999264.90(11522861.15-14605268.10) | 148.56(131.71-166.82) | -36.87(-46.59--26.01) | -1.73(-1.88--1.59) |
|  |  |  |  |  |  |  |
| **SDI** |  |  |  |  |  |  |
| High | 1325836.85(1286515.89-1357512.22) | 123.99(120.47-126.95) | 1825459.35(1719025.54-1902714.15) | 93.95(89.28-97.92) | -24.22(-27.22--20.93) | -1.01(-1.14--0.89) |
| High middle | 3116679.39(2731976.98-3509251.15) | 303.10(265.93-341.10) | 3834290.56(3157463.62-4667629.25) | 192.56(158.70-234.03) | -36.47(-49.74--20.28) | -1.75(-1.96--1.54) |
| Middle SDI | 4083703.38(3438091.26-4731710.37) | 365.58(309.42-422.25) | 5011783.03(4233897.74-5964293.90) | 180.65(153.17-214.63) | -50.59(-60.68--38.31) | -2.57(-2.74--2.41) |
| Low middle | 763111.41(692292.05-878968.80) | 113.84(102.95-130.89) | 1491633.59(1348141.62-1724083.93) | 97.10(87.74-111.84) | -14.71(-23.09--2.78) | -0.60(-0.65--0.54) |
| Low SDI | 460137.99(380390.53-517923.88) | 185.38(154.42-209.22) | 830121.40(701259.16-964023.87) | 148.67(126.11-172.20) | -19.80(-32.98--4.38) | -0.89(-0.97--0.81) |
| **Regions** |  |  |  |  |  |  |
| Andean Latin America | 10237.71(8990.11-11648.22) | 48.90(42.88-55.55) | 19167.84(15515.38-23633.94) | 32.27(26.17-39.76) | -34.00(-48.05--16.25) | -1.43(-1.55--1.31) |
| Australasia | 23048.29(21900.52-24258.96) | 98.88(93.89-104.06) | 41016.96(37625.99-43814.77) | 80.20(74.13-85.45) | -18.90(-24.95--12.58) | -0.75(-0.82--0.67) |
| Caribbean | 26249.37(24499.08-28020.13) | 100.04(93.47-106.74) | 51044.62(44532.60-58194.12) | 94.29(82.30-107.49) | -5.75(-17.10-7.13) | 0.09(-0.05-0.24) |
| Central Asia | 167512.81(160139.79-174957.43) | 344.55(328.85-359.79) | 99946.78(88486.04-112298.83) | 115.48(102.92-128.96) | -66.48(-70.05--62.50) | -3.63(-3.80--3.45) |
| Central Europe | 122470.59(117760.05-127796.12) | 81.56(78.47-85.10) | 146484.73(134849.03-158439.05) | 73.05(67.15-79.02) | -10.43(-18.18--2.79) | -0.62(-0.78--0.47) |
| Central Latin America | 54642.98(53217.30-55742.27) | 63.52(61.67-64.93) | 95847.40(85461.59-107642.60) | 37.71(33.67-42.31) | -40.64(-47.13--33.54) | -1.80(-1.89--1.72) |
| Central Sub-Saharan Africa | 73191.41(53295.46-93507.68) | 291.65(214.18-368.57) | 137614.50(100038.42-180215.13) | 221.54(161.83-289.26) | -24.04(-43.74-2.04) | -1.04(-1.14--0.93) |
| East Asia | 5942205.43(4927390.91-6910479.44) | 638.45(532.30-740.63) | 7069760.58(5660280.66-8736103.07) | 313.94(252.18-387.12) | -50.83(-63.05--36.11) | -2.62(-2.86--2.39) |
| Eastern Europe | 340939.01(334487.54-348026.48) | 119.37(116.94-121.72) | 274133.34(247183.12-298314.37) | 81.25(73.21-88.53) | -31.93(-38.35--25.54) | -1.49(-1.69--1.28) |
| Eastern Sub-Saharan Africa | 304870.73(246943.22-350912.84) | 373.02(305.58-429.13) | 545442.48(452300.01-659237.30) | 292.22(243.43-352.18) | -21.66(-36.49--2.22) | -1.00(-1.08--0.91) |
| High-income Asia Pacific | 255727.76(244997.87-268388.48) | 123.39(118.12-129.46) | 318613.29(290566.27-336610.21) | 74.52(69.25-78.49) | -39.61(-43.60--35.85) | -1.80(-1.94--1.65) |
| High-income North America | 312473.97(301435.11-318672.13) | 94.58(91.53-96.39) | 535320.64(510487.35-552767.21) | 86.13(82.45-88.83) | -8.93(-11.44--6.42) | -0.39(-0.52--0.26) |
| North Africa and Middle East | 124885.76(97851.44-144296.52) | 68.85(54.78-79.48) | 230170.72(191965.83-262990.89) | 48.01(40.39-54.41) | -30.27(-38.94--19.59) | -1.28(-1.34--1.22) |
| Oceania | 1824.93(1365.89-2436.40) | 56.05(42.94-73.41) | 3905.66(3055.62-5016.37) | 47.10(37.29-59.95) | -15.96(-40.79-9.42) | -0.56(-0.60--0.53) |
| South Asia | 714772.67(635598.26-868530.23) | 110.56(97.80-134.35) | 1434796.86(1268757.00-1704573.72) | 91.08(80.54-108.48) | -17.62(-28.11--4.37) | -0.86(-0.97--0.75) |
| Southeast Asia | 206567.93(172764.73-242795.34) | 73.65(61.86-86.17) | 437487.51(374888.45-504000.93) | 61.71(53.19-70.79) | -16.21(-31.22-2.96) | -0.63(-0.68--0.58) |
| Southern Latin America | 82332.42(78919.62-85737.84) | 177.31(169.83-184.56) | 76789.95(72067.82-82062.59) | 88.92(83.56-94.99) | -49.85(-53.13--46.00) | -2.24(-2.45--2.02) |
| Southern Sub-Saharan Africa | 94526.52(85130.90-108239.41) | 318.41(285.54-365.34) | 185848.78(169360.37-204413.81) | 297.67(271.83-326.47) | -6.51(-19.59-6.22) | -0.60(-1.10--0.11) |
| Tropical Latin America | 177723.62(172210.39-182607.16) | 181.09(174.39-186.12) | 348544.22(331533.17-362398.09) | 132.10(125.59-137.34) | -27.05(-30.70--23.63) | -1.03(-1.13--0.93) |
| Western Europe | 651146.88(632892.71-665508.76) | 121.21(118.00-123.91) | 712093.47(672400.78-741301.38) | 85.44(81.46-88.58) | -29.51(-32.04--27.13) | -1.20(-1.29--1.11) |
| Western Sub-Saharan Africa | 66215.47(53020.28-81476.65) | 71.08(57.33-86.89) | 235234.59(175348.41-283945.58) | 110.00(82.38-132.20) | 54.75(23.32-93.17) | 1.95(1.74-2.16) |

**DALY, disability-adjusted life-years; SDI, Socio-demographic index; CI, confidential interval; UI, uncertainty interval.**

**Supplementary Table 4 Age-standardised Incidence of Esophageal Cancer Between 1990 and 2021 at 204 Countries and Territories Level**

|  | **1990** | | **2021** | | **Percentage change 1990-2021**  **ASIR(95%UI)** | **EAPC of**  **ASIR**  **(95% CI)** |
| --- | --- | --- | --- | --- | --- | --- |
|  | **Incidence cases**  **(95%UI)** | **ASIR per 10^5^**  **(95%UI)** | **Incidence cases**  **(95%UI)** | **ASIR per 10^5^**  **(95%UI)** |  |  |
| American Samoa | 0.26(0.21-0.31) | 1.19(1.01-1.44) | 0.73(0.59-0.88) | 1.56(1.29-1.89) | 31.20(0.06-65.34) | 1.40(1.02-1.78) |
| Antigua and Barbuda | 1.75(1.62-1.87) | 3.28(3.05-3.51) | 3.01(2.81-3.23) | 2.83(2.65-3.02) | -13.81(-21.03--5.78) | -0.38(-0.61--0.15) |
| Arab Republic of Egypt | 309.36(270.46-360.76) | 1.18(1.02-1.43) | 611.13(477.50-774.84) | 1.03(0.82-1.30) | -12.60(-32.21-12.99) | -0.45(-0.59--0.31) |
| Argentine Republic | 2230.53(2112.29-2356.93) | 6.98(6.61-7.37) | 2327.49(2151.95-2526.06) | 4.13(3.82-4.47) | -40.87(-45.69--35.38) | -1.60(-1.84--1.36) |
| Australia | 853.75(803.83-909.02) | 4.35(4.10-4.62) | 1853.12(1654.72-1995.60) | 4.06(3.67-4.37) | -6.65(-14.73-1.21) | -0.31(-0.42--0.20) |
| Barbados | 16.37(15.34-17.43) | 5.70(5.36-6.08) | 25.87(20.33-31.86) | 5.01(3.94-6.18) | -12.21(-31.82-10.55) | -0.38(-0.50--0.26) |
| Belize | 1.79(1.67-1.91) | 1.94(1.81-2.07) | 6.46(5.60-7.40) | 2.13(1.85-2.43) | 9.76(-5.66-26.57) | 0.48(-0.01-0.97) |
| Bermuda | 4.33(3.98-4.69) | 6.98(6.41-7.54) | 5.66(4.79-6.77) | 4.19(3.53-5.03) | -40.00(-50.23--26.78) | -1.28(-1.57--0.99) |
| Bolivarian Republic of Venezuela | 231.60(218.99-242.85) | 2.50(2.36-2.62) | 489.57(354.41-653.42) | 1.65(1.20-2.19) | -34.12(-52.18--11.23) | -1.45(-1.59--1.31) |
| Bosnia and Herzegovina | 98.21(83.53-116.12) | 2.31(1.96-2.71) | 131.15(98.10-167.32) | 2.13(1.59-2.71) | -7.79(-32.71-19.98) | -0.26(-0.40--0.11) |
| Brunei Darussalam | 3.03(2.42-3.70) | 3.15(2.50-3.82) | 7.76(6.12-9.48) | 2.26(1.81-2.73) | -28.14(-46.90--3.78) | -0.69(-0.88--0.51) |
| Burkina Faso | 138.41(104.71-172.83) | 3.22(2.47-4.01) | 497.81(346.98-639.32) | 5.44(3.78-7.00) | 68.65(23.90-123.99) | 2.34(2.09-2.59) |
| Canada | 1227.62(1153.90-1302.40) | 3.79(3.56-4.01) | 2994.30(2742.77-3214.30) | 4.20(3.87-4.51) | 10.89(2.34-20.21) | 0.44(0.27-0.60) |
| Central African Republic | 164.59(114.58-203.99) | 13.81(9.73-16.94) | 248.68(166.66-342.79) | 10.72(7.36-14.50) | -22.38(-42.50-1.17) | -0.96(-1.05--0.87) |
| Commonwealth of Dominica | 2.85(2.46-3.34) | 4.83(4.17-5.68) | 4.16(3.33-5.36) | 4.91(3.95-6.27) | 1.67(-23.47-32.01) | 0.18(0.00-0.37) |
| Commonwealth of the Bahamas | 10.02(9.20-10.92) | 6.40(5.88-6.97) | 21.58(17.02-27.15) | 5.13(4.06-6.40) | -19.89(-38.27-2.88) | -0.24(-0.48-0.01) |
| Cook Islands | 0.35(0.28-0.42) | 2.94(2.42-3.53) | 0.65(0.51-0.79) | 2.54(1.99-3.09) | -13.67(-36.21-11.93) | -0.50(-0.59--0.41) |
| Czech Republic | 386.88(354.39-423.68) | 2.85(2.61-3.12) | 663.40(565.58-760.86) | 3.23(2.75-3.71) | 13.24(-4.65-33.88) | 0.36(0.24-0.48) |
| Democratic People's Republic of Korea | 1721.56(1232.19-2303.43) | 10.49(7.67-13.72) | 3101.60(2303.65-4093.05) | 9.19(6.86-12.05) | -12.42(-37.37-24.56) | -1.88(-2.10--1.66) |
| Democratic Republic of Sao Tome and Principe | 1.49(1.18-1.85) | 2.31(1.84-2.85) | 5.10(3.90-6.64) | 4.62(3.55-5.86) | 99.46(56.83-164.12) | 2.78(2.59-2.97) |
| Democratic Republic of the Congo | 1505.11(1044.91-1998.57) | 9.63(6.88-12.61) | 2909.45(1970.65-3907.42) | 7.96(5.48-10.74) | -17.32(-41.83-12.37) | -0.72(-0.82--0.61) |
| Democratic Republic of Timor-Leste | 7.34(5.10-9.79) | 2.58(1.85-3.36) | 17.85(12.88-24.32) | 2.09(1.54-2.84) | -18.74(-44.85-25.80) | -0.64(-0.88--0.40) |
| Democratic Socialist Republic of Sri Lanka | 602.37(507.93-726.61) | 5.67(4.80-6.78) | 1163.80(763.65-1581.90) | 4.26(2.82-5.80) | -24.88(-52.20-7.64) | -0.50(-0.78--0.22) |
| Dominican Republic | 68.14(55.44-82.80) | 1.89(1.56-2.29) | 218.27(164.53-281.71) | 2.17(1.64-2.80) | 14.85(-18.40-58.89) | 0.99(0.81-1.17) |
| Eastern Republic of Uruguay | 328.31(308.51-347.84) | 8.39(7.87-8.87) | 304.72(279.77-329.35) | 5.45(5.00-5.90) | -35.10(-40.47--29.48) | -1.58(-1.81--1.35) |
| Federal Democratic Republic of Ethiopia | 1903.48(1332.97-2449.57) | 9.42(6.65-12.21) | 2105.16(1688.97-2803.99) | 4.97(4.01-6.66) | -47.24(-62.71--25.38) | -2.50(-2.70--2.30) |
| Federal Democratic Republic of Nepal | 461.93(349.01-605.34) | 4.82(3.66-6.22) | 872.63(624.26-1234.34) | 3.77(2.71-5.32) | -21.64(-43.79-8.03) | -0.66(-1.05--0.26) |
| Federal Republic of Germany | 4308.80(4075.95-4553.93) | 3.58(3.39-3.79) | 8290.29(7570.40-8901.19) | 4.60(4.21-4.91) | 28.43(17.56-40.34) | 0.55(0.33-0.76) |
| Federal Republic of Nigeria | 1159.65(888.39-1563.25) | 2.64(2.04-3.53) | 3764.40(2542.65-5059.16) | 4.25(2.92-5.58) | 60.92(15.72-117.55) | 2.15(1.90-2.40) |
| Federal Republic of Somalia | 509.88(346.93-698.52) | 20.31(14.23-27.19) | 923.03(642.10-1242.68) | 14.91(10.25-19.71) | -26.60(-43.80--0.93) | -1.18(-1.28--1.08) |
| Federated States of Micronesia | 1.50(1.18-1.91) | 3.15(2.48-3.93) | 1.98(1.48-2.64) | 2.67(2.06-3.50) | -15.18(-40.29-21.13) | -0.65(-0.69--0.60) |
| Federative Republic of Brazil | 6021.33(5776.08-6196.17) | 6.71(6.39-6.93) | 12532.77(11854.86-13069.12) | 4.93(4.65-5.14) | -26.60(-30.23--23.45) | -0.97(-1.06--0.89) |
| French Republic | 6523.12(6118.55-6913.23) | 8.43(7.92-8.94) | 6594.04(5937.47-7184.39) | 4.99(4.54-5.41) | -40.79(-46.76--34.72) | -1.59(-1.71--1.46) |
| Gabonese Republic | 70.18(52.61-88.29) | 12.32(9.23-15.45) | 110.28(79.51-142.30) | 10.46(7.65-13.41) | -15.08(-36.09-14.94) | -0.69(-0.81--0.58) |
| Georgia | 132.86(115.90-152.42) | 2.10(1.83-2.40) | 62.81(52.60-74.00) | 1.06(0.89-1.25) | -49.43(-58.94--38.24) | -1.39(-1.93--0.86) |
| Grand Duchy of Luxembourg | 26.09(24.63-27.50) | 4.87(4.59-5.14) | 39.49(35.58-43.65) | 3.73(3.37-4.13) | -23.33(-31.23--14.50) | -0.80(-0.98--0.62) |
| Greenland | 5.66(4.82-6.54) | 16.31(14.01-18.85) | 7.88(6.22-9.68) | 10.81(8.59-13.41) | -33.72(-49.70--14.60) | -1.22(-1.27--1.16) |
| Grenada | 4.42(3.96-4.91) | 6.41(5.73-7.13) | 5.49(4.63-6.35) | 4.63(3.93-5.32) | -27.82(-40.14--14.21) | -0.59(-1.09--0.10) |
| Guam | 1.65(1.42-1.91) | 2.32(2.00-2.64) | 3.98(3.32-4.70) | 1.88(1.58-2.22) | -18.89(-35.49-0.26) | 0.20(-0.17-0.58) |
| Hashemite Kingdom of Jordan | 14.50(11.44-18.06) | 1.12(0.88-1.37) | 61.35(47.30-78.61) | 0.85(0.66-1.08) | -23.57(-45.12-5.65) | -1.10(-1.34--0.87) |
| Hellenic Republic | 345.14(323.62-364.25) | 2.25(2.11-2.37) | 409.37(373.14-435.36) | 1.82(1.69-1.93) | -19.19(-25.27--13.35) | -0.97(-1.18--0.76) |
| Hungary | 594.28(550.02-637.17) | 4.20(3.90-4.50) | 569.65(502.77-645.21) | 3.17(2.80-3.59) | -24.55(-33.49--12.69) | -1.40(-1.71--1.09) |
| Independent State of Papua New Guinea | 33.80(23.27-49.85) | 1.93(1.33-2.81) | 76.74(53.30-109.00) | 1.58(1.11-2.24) | -18.00(-49.11-22.22) | -0.74(-0.79--0.68) |
| Independent State of Samoa | 1.05(0.82-1.30) | 1.26(1.00-1.53) | 1.60(1.25-2.05) | 1.12(0.88-1.42) | -11.08(-32.19-21.44) | -0.39(-0.46--0.32) |
| Ireland | 316.32(297.41-338.12) | 7.77(7.29-8.28) | 485.92(429.75-536.36) | 6.13(5.45-6.74) | -21.02(-29.22--12.21) | -0.50(-0.60--0.39) |
| Islamic Republic of Afghanistan | 659.05(317.76-949.86) | 9.32(4.62-13.26) | 716.75(331.27-1081.89) | 7.26(3.56-10.67) | -22.08(-43.26-12.19) | -1.00(-1.16--0.84) |
| Islamic Republic of Iran | 1221.82(1049.22-1367.08) | 5.02(4.28-5.64) | 2791.16(2528.06-3035.00) | 3.78(3.40-4.11) | -24.84(-34.45--12.44) | -0.93(-1.03--0.83) |
| Islamic Republic of Mauritania | 35.07(26.96-44.86) | 3.54(2.74-4.54) | 110.19(77.78-151.57) | 5.21(3.70-7.09) | 47.27(3.92-103.20) | 1.68(1.34-2.02) |
| Islamic Republic of Pakistan | 3464.73(2919.17-3984.79) | 6.16(5.18-7.06) | 7672.59(6079.73-9653.42) | 6.26(4.97-7.79) | 1.61(-19.87-32.96) | -0.30(-0.53--0.07) |
| Jamaica | 51.90(48.34-55.87) | 2.91(2.71-3.13) | 91.77(69.57-119.15) | 2.97(2.25-3.84) | 2.03(-22.83-32.71) | 0.11(-0.35-0.57) |
| Japan | 11335.28(10815.97-11675.71) | 6.59(6.28-6.80) | 21932.89(19704.52-23180.45) | 6.22(5.73-6.48) | -5.70(-9.56--2.33) | -0.24(-0.48--0.01) |
| Kingdom of Bahrain | 5.75(4.84-6.69) | 3.84(3.29-4.45) | 15.71(12.31-20.10) | 2.17(1.74-2.67) | -43.42(-56.10--26.78) | -2.70(-3.07--2.32) |
| Kingdom of Belgium | 610.57(569.85-649.20) | 4.10(3.84-4.35) | 1030.72(940.32-1116.40) | 4.59(4.23-4.95) | 12.05(2.09-21.62) | 0.36(0.14-0.58) |
| Kingdom of Bhutan | 12.46(8.87-17.07) | 5.01(3.58-6.79) | 21.38(15.45-29.59) | 3.56(2.60-4.86) | -29.03(-51.07-7.25) | -1.17(-1.38--0.95) |
| Kingdom of Cambodia | 195.51(151.49-246.76) | 4.23(3.28-5.28) | 368.11(267.75-485.93) | 2.96(2.17-3.87) | -30.02(-50.48--3.35) | -1.33(-1.44--1.23) |
| Kingdom of Denmark | 374.45(354.89-395.65) | 4.82(4.57-5.08) | 595.20(537.51-640.12) | 5.10(4.64-5.46) | 5.77(-3.54-14.70) | 0.12(-0.01-0.25) |
| Kingdom of Eswatini | 48.07(35.75-61.40) | 16.40(12.26-20.70) | 99.15(69.45-136.95) | 16.68(11.80-22.42) | 1.72(-26.59-45.46) | 0.31(-0.36-0.97) |
| Kingdom of Lesotho | 86.22(66.75-109.51) | 10.20(7.89-13.05) | 175.82(129.87-225.07) | 15.82(11.67-20.12) | 55.06(12.36-118.39) | 2.00(1.53-2.47) |
| Kingdom of Morocco | 114.19(88.92-140.49) | 0.81(0.63-1.00) | 290.49(210.24-367.02) | 0.85(0.62-1.06) | 4.65(-23.86-36.81) | 0.25(0.12-0.37) |
| Kingdom of Norway | 174.28(165.77-181.00) | 2.63(2.51-2.72) | 334.57(307.62-353.50) | 3.32(3.09-3.50) | 26.51(19.89-33.01) | 0.74(0.59-0.89) |
| Kingdom of Saudi Arabia | 120.15(84.95-161.43) | 2.15(1.56-2.87) | 331.42(253.49-445.89) | 1.75(1.38-2.23) | -18.56(-43.71-14.15) | -0.99(-1.12--0.86) |
| Kingdom of Spain | 2246.88(2115.45-2374.21) | 4.28(4.03-4.52) | 2563.41(2312.09-2769.59) | 2.81(2.57-3.03) | -34.32(-39.60--28.28) | -1.37(-1.46--1.29) |
| Kingdom of Sweden | 416.46(389.28-438.67) | 2.76(2.61-2.90) | 624.52(541.43-697.46) | 2.86(2.50-3.19) | 3.55(-9.48-16.29) | 0.22(0.06-0.39) |
| Kingdom of Thailand | 1759.87(1358.92-2358.59) | 4.83(3.68-6.45) | 5144.47(3850.43-6747.48) | 4.72(3.56-6.18) | -2.28(-34.28-47.11) | -0.39(-0.52--0.26) |
| Kingdom of the Netherlands | 943.20(883.77-997.74) | 4.79(4.47-5.05) | 2440.35(2219.06-2635.22) | 6.88(6.30-7.40) | 43.86(32.88-54.52) | 1.37(1.07-1.67) |
| Kingdom of Tonga | 1.07(0.85-1.33) | 2.01(1.60-2.50) | 1.57(1.20-2.04) | 1.98(1.52-2.57) | -1.23(-28.28-32.71) | -0.15(-0.36-0.05) |
| Kyrgyz Republic | 247.50(213.43-283.80) | 8.27(7.17-9.44) | 132.35(103.75-164.38) | 2.82(2.25-3.44) | -65.91(-72.88--56.54) | -3.38(-3.50--3.27) |
| Lao People's Democratic Republic | 93.01(64.99-126.12) | 4.34(3.07-5.83) | 115.40(85.38-160.58) | 2.48(1.86-3.42) | -42.90(-62.78--13.66) | -1.98(-2.07--1.89) |
| Lebanese Republic | 27.46(21.44-35.09) | 1.28(1.01-1.64) | 55.83(44.42-68.95) | 0.92(0.73-1.13) | -28.57(-46.84--0.78) | -0.71(-0.91--0.52) |
| Malaysia | 237.27(197.79-275.11) | 2.61(2.18-3.04) | 749.03(636.39-873.28) | 2.65(2.25-3.08) | 1.24(-18.93-25.46) | -0.12(-0.27-0.03) |
| Mongolia | 236.16(193.72-288.61) | 23.27(18.96-28.49) | 338.61(269.79-410.85) | 16.25(13.15-19.45) | -30.17(-45.99--9.74) | -1.50(-1.65--1.36) |
| Montenegro | 12.64(10.39-15.49) | 1.96(1.61-2.41) | 21.57(16.55-27.74) | 2.22(1.71-2.85) | 13.36(-17.14-61.65) | 0.60(0.50-0.71) |
| New Zealand | 201.06(187.88-213.02) | 5.09(4.76-5.39) | 348.97(317.67-377.64) | 4.04(3.69-4.36) | -20.67(-26.46--13.35) | -0.81(-1.00--0.62) |
| North Macedonia | 26.85(23.33-31.52) | 1.41(1.24-1.64) | 44.99(34.48-57.56) | 1.37(1.06-1.74) | -2.85(-25.94-28.55) | -0.18(-0.46-0.11) |
| Northern Mariana Islands | 0.23(0.18-0.30) | 1.37(1.11-1.76) | 1.29(1.06-1.52) | 2.60(2.14-3.09) | 89.09(38.28-147.51) | 3.11(2.50-3.72) |
| Palestine | 11.18(8.20-14.54) | 1.37(1.01-1.76) | 20.81(16.74-25.54) | 0.90(0.73-1.10) | -34.34(-53.30--7.89) | -1.58(-1.78--1.38) |
| People's Democratic Republic of Algeria | 82.06(67.17-98.52) | 0.74(0.61-0.88) | 234.13(179.94-294.78) | 0.70(0.55-0.87) | -5.71(-30.83-22.74) | -0.08(-0.14--0.02) |
| People's Republic of Bangladesh | 2461.90(1757.18-3274.14) | 5.14(3.66-6.81) | 4507.90(3207.49-6246.77) | 3.27(2.34-4.51) | -36.27(-55.20--10.77) | -1.43(-1.56--1.30) |
| People's Republic of China | 207494.92(172673.51-241458.64) | 24.80(20.71-28.73) | 320805.43(256102.37-394756.17) | 15.04(12.04-18.43) | -39.35(-53.47--21.89) | -1.88(-2.10--1.66) |
| Plurinational State of Bolivia | 84.86(64.61-107.91) | 2.78(2.13-3.53) | 191.43(140.37-257.34) | 2.21(1.64-2.95) | -20.41(-42.61-13.37) | -0.74(-0.82--0.65) |
| Portuguese Republic | 632.24(592.27-669.36) | 4.64(4.35-4.92) | 756.15(689.00-820.39) | 3.44(3.14-3.73) | -25.84(-33.16--17.95) | -0.84(-0.98--0.69) |
| Principality of Andorra | 1.15(0.80-1.66) | 2.00(1.39-2.87) | 2.08(1.34-3.01) | 1.35(0.87-1.95) | -32.51(-61.55-10.31) | -0.95(-1.23--0.67) |
| Principality of Monaco | 3.47(2.62-4.38) | 5.12(3.88-6.40) | 5.84(4.64-7.15) | 6.21(4.91-7.70) | 21.44(-11.61-67.15) | 0.81(0.57-1.06) |
| Puerto Rico | 218.15(204.87-232.00) | 6.06(5.72-6.44) | 164.12(134.65-193.01) | 2.42(1.98-2.85) | -60.05(-67.51--52.39) | -2.84(-3.06--2.62) |
| Republic of Albania | 38.03(31.81-45.54) | 1.88(1.58-2.25) | 60.96(44.50-80.12) | 1.41(1.03-1.85) | -25.03(-46.47-6.18) | -0.65(-0.90--0.41) |
| Republic of Angola | 466.74(312.02-636.93) | 11.66(8.07-15.62) | 937.05(671.39-1234.79) | 7.97(5.67-10.37) | -31.68(-51.54--0.23) | -1.44(-1.55--1.32) |
| Republic of Armenia | 74.53(68.52-80.27) | 2.77(2.54-3.00) | 44.91(39.42-51.40) | 1.00(0.88-1.15) | -63.71(-69.35--57.10) | -3.12(-3.48--2.77) |
| Republic of Austria | 301.05(283.54-318.11) | 2.67(2.52-2.82) | 467.44(423.56-503.96) | 2.67(2.44-2.87) | 0.01(-7.87-8.91) | 0.22(0.06-0.37) |
| Republic of Azerbaijan | 416.89(361.33-473.04) | 8.33(7.25-9.56) | 534.50(394.68-686.86) | 5.35(3.96-6.87) | -35.75(-53.40--16.62) | -1.25(-1.45--1.05) |
| Republic of Belarus | 377.09(347.24-407.54) | 2.86(2.63-3.10) | 487.37(398.47-601.99) | 3.05(2.50-3.75) | 6.32(-14.98-32.59) | 2.11(1.87-2.35) |
| Republic of Benin | 61.58(50.44-74.65) | 3.14(2.58-3.80) | 256.52(192.58-335.59) | 5.02(3.75-6.50) | 60.05(16.89-115.53) | -0.23(-0.40--0.05) |
| Republic of Botswana | 67.33(48.19-89.77) | 12.02(8.68-15.78) | 125.21(93.72-163.02) | 8.59(6.53-11.12) | -28.60(-50.29-2.25) | -1.33(-1.61--1.05) |
| Republic of Bulgaria | 307.09(286.15-331.25) | 2.51(2.35-2.69) | 295.11(252.99-341.41) | 2.26(1.93-2.60) | -9.91(-23.71-6.01) | -0.61(-0.87--0.34) |
| Republic of Burundi | 445.42(321.34-558.48) | 19.07(13.76-23.80) | 515.89(369.77-670.22) | 10.64(7.73-13.65) | -44.20(-59.52--22.73) | -2.49(-2.75--2.23) |
| Republic of Cabo Verde | 19.91(16.38-23.73) | 8.69(7.21-10.29) | 67.26(52.57-83.15) | 15.15(11.90-18.91) | 74.44(32.32-128.97) | 1.29(0.88-1.70) |
| Republic of Cameroon | 156.18(118.54-197.36) | 3.53(2.73-4.41) | 797.73(537.56-1099.30) | 6.27(4.33-8.52) | 77.52(26.44-147.72) | 2.36(2.15-2.58) |
| Republic of Chad | 70.75(57.02-85.67) | 2.53(2.05-3.07) | 314.04(229.26-413.74) | 5.44(4.00-7.04) | 115.15(59.66-202.26) | 3.05(2.83-3.28) |
| Republic of Chile | 716.87(677.90-753.09) | 7.42(6.99-7.80) | 799.76(723.98-862.39) | 3.07(2.79-3.31) | -58.57(-61.81--54.94) | -3.07(-3.25--2.89) |
| Republic of Colombia | 683.81(647.30-716.16) | 4.06(3.83-4.28) | 973.24(809.83-1156.93) | 1.77(1.47-2.10) | -56.55(-63.73--47.75) | -3.15(-3.32--2.99) |
| Republic of Costa Rica | 42.15(38.98-45.10) | 2.47(2.28-2.65) | 85.95(74.93-96.28) | 1.56(1.37-1.75) | -36.73(-44.77--28.69) | -1.69(-1.84--1.55) |
| Republic of Croatia | 234.46(217.64-251.02) | 3.76(3.51-4.01) | 226.23(195.96-255.59) | 2.67(2.33-3.03) | -28.90(-39.74--19.00) | -1.11(-1.29--0.94) |
| Republic of Cuba | 376.83(357.29-398.94) | 3.71(3.52-3.92) | 973.43(829.70-1121.86) | 4.97(4.22-5.72) | 33.96(14.91-55.76) | 1.37(1.19-1.55) |
| Republic of Cyprus | 10.38(8.45-12.75) | 1.43(1.16-1.75) | 30.03(23.37-37.94) | 1.47(1.15-1.85) | 2.80(-23.83-38.84) | 0.83(0.55-1.10) |
| Republic of Cote d'Ivoire | 51.15(40.69-62.00) | 1.29(1.06-1.54) | 145.60(107.44-197.70) | 1.30(0.99-1.73) | 0.62(-24.42-38.59) | -0.00(-0.17-0.17) |
| Republic of Djibouti | 20.43(14.31-28.59) | 14.72(10.59-19.93) | 77.69(51.27-113.53) | 12.11(8.24-17.11) | -17.72(-42.62-19.41) | -0.84(-0.94--0.73) |
| Republic of Ecuador | 103.16(96.64-109.13) | 2.03(1.90-2.16) | 190.75(150.05-239.39) | 1.19(0.94-1.49) | -41.44(-53.78--26.32) | -1.38(-1.65--1.11) |
| Republic of El Salvador | 53.71(47.88-59.81) | 1.83(1.63-2.04) | 104.85(83.55-132.39) | 1.69(1.34-2.13) | -7.92(-28.88-16.57) | -0.54(-0.74--0.34) |
| Republic of Equatorial Guinea | 26.29(18.22-34.74) | 13.15(9.28-17.19) | 44.04(30.14-63.24) | 8.78(6.13-12.50) | -33.24(-55.72--1.74) | -1.47(-1.84--1.09) |
| Republic of Estonia | 68.32(63.52-73.26) | 3.32(3.09-3.56) | 69.58(59.47-80.19) | 2.75(2.35-3.18) | -17.12(-30.36--1.68) | -0.86(-1.11--0.60) |
| Republic of Fiji | 7.73(6.15-9.71) | 2.26(1.81-2.83) | 18.85(13.50-24.82) | 2.63(1.88-3.42) | 15.96(-18.04-60.38) | 0.66(0.50-0.83) |
| Republic of Finland | 260.87(244.86-274.41) | 3.69(3.47-3.87) | 440.79(401.18-474.41) | 3.62(3.32-3.86) | -1.94(-8.82-6.06) | 0.05(-0.03-0.13) |
| Republic of Ghana | 149.83(114.70-189.59) | 2.41(1.87-3.02) | 571.68(386.53-736.94) | 3.51(2.33-4.55) | 45.48(0.84-97.86) | 1.72(1.54-1.91) |
| Republic of Guatemala | 79.43(76.11-82.69) | 2.56(2.45-2.66) | 176.93(151.33-209.97) | 1.65(1.41-1.95) | -35.51(-44.99--24.21) | -1.62(-1.89--1.35) |
| Republic of Guinea | 39.60(30.87-48.62) | 1.20(0.93-1.47) | 75.20(52.95-101.40) | 1.34(0.96-1.80) | 12.18(-27.30-64.71) | 0.61(0.46-0.76) |
| Republic of Guinea-Bissau | 18.99(14.10-23.85) | 4.71(3.55-5.87) | 54.04(40.26-68.14) | 7.31(5.45-9.12) | 55.09(15.27-117.05) | 2.09(1.83-2.34) |
| Republic of Guyana | 7.93(7.09-8.88) | 2.10(1.87-2.33) | 13.59(10.35-17.58) | 2.04(1.56-2.62) | -2.87(-27.75-27.17) | 0.26(0.08-0.44) |
| Republic of Haiti | 168.67(120.30-221.15) | 5.23(3.80-6.74) | 283.74(196.04-399.59) | 3.95(2.78-5.50) | -24.61(-47.21-3.15) | -0.72(-0.82--0.62) |
| Republic of Honduras | 19.97(16.39-23.77) | 1.00(0.83-1.19) | 87.38(67.91-111.23) | 1.45(1.14-1.83) | 44.59(9.64-92.18) | 1.54(1.36-1.73) |
| Republic of Iceland | 13.29(12.29-14.20) | 4.71(4.37-5.03) | 25.82(22.94-28.81) | 4.53(4.04-5.05) | -3.81(-13.87-8.21) | -0.19(-0.34--0.04) |
| Republic of India | 16820.82(14654.08-20952.46) | 3.49(3.02-4.38) | 37006.84(32442.81-44293.13) | 3.07(2.69-3.69) | -11.94(-23.81-4.30) | -0.66(-0.79--0.54) |
| Republic of Indonesia | 1843.65(1368.87-2236.36) | 1.84(1.38-2.24) | 3846.73(2948.58-4854.81) | 1.62(1.25-2.03) | -12.03(-31.25-11.88) | -0.41(-0.47--0.36) |
| Republic of Iraq | 93.68(72.35-118.19) | 1.18(0.92-1.49) | 274.30(196.82-353.58) | 1.18(0.87-1.51) | -0.44(-32.41-42.08) | -0.34(-0.50--0.18) |
| Republic of Italy | 2910.45(2763.16-3011.79) | 3.34(3.17-3.45) | 2510.25(2268.34-2658.02) | 1.77(1.64-1.87) | -46.86(-49.20--44.53) | -2.03(-2.10--1.97) |
| Republic of Kazakhstan | 2355.13(2187.85-2537.31) | 18.88(17.51-20.34) | 831.61(712.08-960.93) | 4.69(4.04-5.40) | -75.17(-78.81--70.95) | -4.55(-4.80--4.30) |
| Republic of Kenya | 736.53(530.15-1059.44) | 8.97(6.42-12.97) | 2642.50(1923.38-3713.25) | 11.75(8.54-16.66) | 30.94(-0.13-73.68) | 1.28(1.05-1.52) |
| Republic of Kiribati | 2.23(1.74-2.77) | 6.09(4.80-7.54) | 3.95(2.82-5.17) | 5.52(3.95-7.07) | -9.46(-35.48-24.52) | -0.44(-0.54--0.33) |
| Republic of Korea | 1839.89(1490.69-2230.41) | 6.02(4.91-7.28) | 3382.70(2698.84-4139.33) | 3.52(2.81-4.31) | -41.48(-56.50--20.82) | -2.08(-2.22--1.94) |
| Republic of Latvia | 109.82(101.86-118.00) | 3.05(2.82-3.28) | 106.02(90.89-120.75) | 2.91(2.50-3.33) | -4.40(-18.36-10.47) | -0.16(-0.35-0.02) |
| Republic of Liberia | 35.11(27.48-43.73) | 3.08(2.44-3.81) | 119.78(82.37-170.49) | 5.65(3.97-7.88) | 83.41(29.41-157.70) | 2.59(2.25-2.94) |
| Republic of Lithuania | 145.02(135.63-155.49) | 3.19(2.98-3.41) | 194.57(167.70-222.77) | 3.68(3.17-4.21) | 15.55(-1.58-32.39) | 0.48(0.29-0.67) |
| Republic of Madagascar | 742.38(546.74-906.05) | 14.45(10.71-17.47) | 1233.01(850.48-1700.88) | 10.79(7.47-14.78) | -25.33(-46.42-0.26) | -1.06(-1.15--0.96) |
| Republic of Malawi | 811.94(660.48-982.00) | 21.08(17.24-25.27) | 1965.11(1578.83-2478.71) | 26.06(21.02-32.46) | 23.67(-5.04-60.25) | 0.67(0.27-1.07) |
| Republic of Maldives | 2.83(2.00-3.65) | 3.04(2.23-3.87) | 4.04(3.11-5.04) | 1.21(0.95-1.48) | -60.24(-70.27--42.27) | -3.43(-3.65--3.21) |
| Republic of Mali | 96.09(80.42-113.52) | 2.41(2.03-2.83) | 214.37(163.36-275.77) | 2.45(1.87-3.12) | 1.32(-29.23-34.71) | 0.33(0.20-0.47) |
| Republic of Malta | 12.27(11.34-13.24) | 2.87(2.66-3.10) | 22.29(19.61-24.82) | 2.41(2.15-2.68) | -16.08(-26.31--5.54) | -0.54(-0.68--0.40) |
| Republic of Mauritius | 26.21(24.87-27.61) | 3.61(3.42-3.80) | 66.19(61.38-70.13) | 3.55(3.30-3.75) | -1.73(-10.97-5.86) | -0.68(-1.10--0.27) |
| Republic of Moldova | 110.37(103.65-116.34) | 2.46(2.32-2.59) | 112.33(100.44-126.30) | 1.86(1.67-2.09) | -24.20(-32.84--13.30) | -0.78(-1.26--0.29) |
| Republic of Mozambique | 422.73(334.72-524.65) | 7.58(5.99-9.25) | 949.91(715.69-1194.75) | 8.98(6.90-11.19) | 18.43(-14.57-55.05) | 1.04(0.86-1.23) |
| Republic of Namibia | 15.51(12.72-18.98) | 2.35(1.95-2.85) | 33.76(25.60-45.15) | 2.35(1.83-3.08) | 0.16(-26.51-38.25) | -0.20(-0.54-0.13) |
| Republic of Nauru | 0.18(0.13-0.24) | 3.99(2.94-5.17) | 0.19(0.14-0.25) | 3.25(2.41-4.19) | -18.50(-41.00-11.08) | -0.92(-1.05--0.79) |
| Republic of Nicaragua | 15.03(12.91-16.90) | 1.02(0.87-1.15) | 39.44(31.72-48.69) | 0.82(0.66-1.02) | -19.12(-35.85-2.57) | -0.59(-0.83--0.35) |
| Republic of Niue | 0.05(0.04-0.06) | 2.30(1.85-2.81) | 0.05(0.04-0.06) | 2.41(1.87-2.95) | 4.75(-21.82-37.48) | -0.07(-0.17-0.03) |
| Republic of Palau | 0.28(0.22-0.36) | 3.14(2.51-3.97) | 0.59(0.47-0.74) | 2.89(2.35-3.58) | -8.07(-32.10-22.52) | -0.24(-0.28--0.19) |
| Republic of Panama | 26.52(24.67-28.35) | 1.84(1.71-1.97) | 53.33(41.55-64.49) | 1.20(0.94-1.46) | -34.51(-48.46--21.25) | -1.21(-1.36--1.05) |
| Republic of Paraguay | 68.47(56.90-82.34) | 3.12(2.59-3.75) | 234.17(173.73-314.87) | 4.01(2.99-5.40) | 28.57(-10.55-78.37) | 0.78(0.63-0.93) |
| Republic of Peru | 197.50(162.61-239.20) | 1.72(1.42-2.07) | 419.34(312.22-556.97) | 1.27(0.94-1.68) | -26.29(-47.96-4.37) | -1.30(-1.60--0.99) |
| Republic of Poland | 1534.61(1476.58-1584.87) | 3.54(3.40-3.66) | 2061.77(1863.63-2248.62) | 2.98(2.69-3.25) | -15.75(-23.79--8.04) | -0.83(-1.00--0.67) |
| Republic of Rwanda | 611.04(418.24-773.75) | 21.07(14.53-26.51) | 698.16(507.06-924.86) | 11.28(8.26-14.89) | -46.46(-62.75--23.46) | -3.05(-3.47--2.62) |
| Republic of San Marino | 0.58(0.45-0.71) | 1.67(1.31-2.07) | 0.68(0.44-1.00) | 0.96(0.60-1.42) | -42.51(-64.72--8.08) | -0.79(-1.17--0.41) |
| Republic of Senegal | 97.04(77.90-117.58) | 3.02(2.45-3.64) | 387.74(291.97-505.88) | 5.06(3.82-6.54) | 67.34(25.84-124.77) | 2.29(1.94-2.65) |
| Republic of Serbia | 268.88(201.24-363.53) | 2.37(1.80-3.17) | 340.29(245.95-464.93) | 2.14(1.54-2.94) | -9.67(-39.50-37.03) | -0.43(-0.57--0.30) |
| Republic of Seychelles | 3.02(2.54-3.57) | 5.41(4.54-6.38) | 5.70(4.59-6.95) | 4.73(3.85-5.73) | -12.60(-31.26-14.24) | -0.33(-0.51--0.15) |
| Republic of Sierra Leone | 56.15(43.83-69.89) | 2.78(2.19-3.43) | 181.20(129.99-241.72) | 4.83(3.47-6.39) | 73.77(28.34-133.30) | 2.55(2.25-2.86) |
| Republic of Singapore | 102.23(95.97-108.77) | 4.71(4.42-5.02) | 223.73(201.84-243.71) | 2.59(2.33-2.81) | -45.10(-50.87--39.18) | -1.92(-2.26--1.57) |
| Republic of Slovenia | 85.31(80.02-90.92) | 3.42(3.21-3.64) | 103.89(89.11-117.44) | 2.44(2.08-2.76) | -28.72(-39.57--18.85) | -1.41(-1.57--1.24) |
| Republic of South Africa | 2379.39(2101.28-2812.48) | 11.16(9.82-13.42) | 4856.66(4409.77-5432.83) | 10.43(9.45-11.60) | -6.58(-22.33-8.01) | -0.73(-1.25--0.21) |
| Republic of South Sudan | 449.15(331.06-610.84) | 17.52(12.94-23.53) | 599.44(429.64-799.21) | 15.16(11.11-20.13) | -13.52(-35.49-20.63) | -0.74(-0.93--0.55) |
| Republic of Sudan | 439.23(273.70-584.95) | 4.74(2.99-6.29) | 809.69(497.96-1154.48) | 4.24(2.70-5.95) | -10.68(-37.45-28.68) | -0.41(-0.47--0.35) |
| Republic of Suriname | 4.45(3.82-5.13) | 1.74(1.50-2.01) | 10.44(7.77-13.65) | 1.61(1.20-2.08) | -7.83(-36.00-23.81) | 0.02(-0.21-0.25) |
| Republic of Tajikistan | 307.71(252.41-368.92) | 11.40(9.40-13.62) | 327.98(241.84-428.03) | 6.13(4.58-7.87) | -46.22(-61.74--24.96) | -2.06(-2.29--1.82) |
| Republic of the Congo | 169.58(126.31-224.00) | 15.68(11.83-20.39) | 286.83(201.96-390.51) | 10.48(7.65-14.00) | -33.14(-51.26--9.74) | -1.60(-1.77--1.44) |
| Republic of the Gambia | 5.18(3.94-6.54) | 1.50(1.16-1.88) | 18.69(14.33-23.56) | 1.93(1.49-2.43) | 29.14(-8.68-73.12) | 0.80(0.67-0.92) |
| Republic of the Marshall Islands | 0.44(0.30-0.58) | 2.77(1.94-3.63) | 0.86(0.62-1.19) | 2.48(1.86-3.33) | -10.36(-36.35-27.05) | -0.27(-0.35--0.18) |
| Republic of the Niger | 76.53(57.20-96.39) | 2.80(2.11-3.49) | 329.11(226.93-440.88) | 4.11(2.84-5.43) | 47.17(5.90-105.38) | 1.91(1.64-2.18) |
| Republic of the Philippines | 416.15(366.60-484.59) | 1.37(1.21-1.59) | 1130.96(923.42-1345.81) | 1.34(1.10-1.60) | -2.35(-21.02-19.65) | -0.02(-0.11-0.08) |
| Republic of the Union of Myanmar | 936.08(698.42-1201.35) | 3.92(2.96-5.00) | 1103.75(847.42-1499.90) | 2.25(1.75-3.04) | -42.56(-60.52--14.26) | -2.13(-2.25--2.00) |
| Republic of Trinidad and Tobago | 19.20(18.13-20.31) | 2.33(2.21-2.46) | 37.48(28.59-47.62) | 1.93(1.47-2.44) | -17.47(-37.25-6.14) | -0.61(-0.85--0.37) |
| Republic of Tunisia | 32.21(25.71-39.62) | 0.66(0.53-0.82) | 88.18(60.57-123.95) | 0.67(0.46-0.93) | 0.40(-33.10-44.14) | -0.08(-0.12--0.04) |
| Republic of Turkey | 823.26(669.79-989.28) | 2.39(1.95-2.87) | 1509.53(1143.00-1918.15) | 1.63(1.24-2.07) | -31.81(-50.03--10.93) | -1.34(-1.62--1.05) |
| Republic of Uganda | 924.29(733.12-1142.27) | 14.52(11.61-17.73) | 2180.52(1672.91-2867.36) | 14.81(11.47-19.57) | 2.00(-29.64-41.95) | -0.36(-0.60--0.11) |
| Republic of Uzbekistan | 1623.14(1474.75-1776.37) | 14.18(12.85-15.51) | 964.13(795.11-1152.30) | 3.71(3.09-4.42) | -73.83(-78.40--68.94) | -4.70(-5.34--4.05) |
| Republic of Vanuatu | 1.51(1.10-2.04) | 2.47(1.85-3.26) | 3.77(2.92-4.88) | 2.21(1.73-2.82) | -10.34(-38.93-24.14) | -0.46(-0.51--0.40) |
| Republic of Yemen | 243.44(140.13-347.12) | 5.01(2.86-7.18) | 526.43(273.47-756.10) | 3.85(2.03-5.50) | -23.28(-48.94-17.74) | -1.11(-1.21--1.01) |
| Republic of Zambia | 557.52(431.21-687.85) | 19.30(14.97-23.88) | 1133.41(751.73-1742.53) | 16.02(10.95-24.45) | -16.98(-42.86-29.17) | -1.08(-1.30--0.85) |
| Republic of Zimbabwe | 503.27(404.44-606.77) | 12.58(10.21-15.06) | 1119.57(843.97-1401.82) | 15.93(12.31-19.64) | 26.59(-7.85-67.26) | 0.92(0.46-1.38) |
| Romania | 418.81(395.19-447.56) | 1.49(1.41-1.59) | 856.04(750.83-970.43) | 2.52(2.20-2.86) | 68.93(45.97-93.52) | 1.25(0.95-1.54) |
| Russian Federation | 9161.77(8974.07-9327.00) | 4.99(4.88-5.08) | 7919.24(7176.80-8607.86) | 3.33(3.01-3.61) | -33.38(-39.69--27.81) | -1.51(-1.75--1.27) |
| Saint Kitts and Nevis | 1.62(1.51-1.72) | 4.43(4.15-4.70) | 2.71(2.23-3.20) | 3.81(3.17-4.44) | -14.14(-29.12-2.12) | -0.02(-0.27-0.23) |
| Saint Lucia | 4.32(4.07-4.60) | 5.11(4.82-5.44) | 10.07(8.20-12.10) | 4.14(3.39-4.98) | -18.91(-33.76--1.27) | -0.73(-1.02--0.45) |
| Saint Vincent and the Grenadines | 1.62(1.48-1.75) | 2.31(2.12-2.48) | 3.55(3.11-4.05) | 2.47(2.17-2.81) | 7.22(-8.27-25.40) | 0.37(0.08-0.65) |
| Slovak Republic | 243.73(189.86-313.89) | 4.13(3.23-5.32) | 299.68(229.52-398.03) | 3.25(2.47-4.33) | -21.40(-46.84-13.92) | -0.76(-0.88--0.64) |
| Socialist Republic of Viet Nam | 823.05(611.94-1081.92) | 2.03(1.52-2.66) | 2425.57(1797.50-3097.43) | 2.33(1.77-2.94) | 14.53(-19.06-68.75) | 0.64(0.57-0.72) |
| Solomon Islands | 3.67(2.41-4.95) | 2.74(1.95-3.60) | 8.35(5.58-11.75) | 2.40(1.65-3.33) | -12.21(-40.80-34.31) | -0.48(-0.54--0.41) |
| State of Eritrea | 246.81(160.09-330.07) | 20.19(13.46-26.41) | 401.82(278.93-579.86) | 14.47(10.20-20.82) | -28.32(-45.60--5.28) | -1.21(-1.30--1.12) |
| State of Israel | 95.59(88.89-102.51) | 2.00(1.85-2.13) | 190.34(171.08-205.86) | 1.54(1.41-1.67) | -22.63(-29.60--15.52) | -1.00(-1.16--0.83) |
| State of Kuwait | 10.67(9.80-11.54) | 1.82(1.66-1.98) | 25.56(20.71-31.27) | 0.94(0.76-1.15) | -48.42(-58.17--36.84) | -2.56(-3.21--1.91) |
| State of Libya | 26.03(18.95-35.20) | 1.39(1.02-1.87) | 90.26(62.69-121.54) | 1.70(1.20-2.26) | 21.89(-20.57-88.11) | 0.96(0.80-1.12) |
| State of Qatar | 4.47(3.49-5.61) | 5.03(3.98-6.22) | 20.21(14.56-27.66) | 2.59(1.93-3.43) | -48.47(-64.49--25.17) | -2.55(-3.19--1.91) |
| Sultanate of Oman | 12.66(9.08-16.88) | 1.92(1.39-2.54) | 30.08(21.92-38.71) | 1.59(1.19-2.00) | -17.53(-43.58-23.71) | -0.33(-0.50--0.16) |
| Swiss Confederation | 459.80(433.02-485.71) | 4.58(4.31-4.83) | 574.70(511.38-623.61) | 3.22(2.90-3.49) | -29.56(-35.83--23.25) | -0.76(-0.99--0.54) |
| Syrian Arab Republic | 42.99(33.53-53.87) | 0.85(0.67-1.06) | 110.32(79.34-145.67) | 0.89(0.66-1.15) | 5.10(-27.63-54.15) | -0.11(-0.29-0.07) |
| Taiwan (Province of China) | 1470.52(1395.04-1556.97) | 9.05(8.61-9.58) | 3798.95(3434.33-4120.22) | 9.30(8.41-10.06) | 2.76(-8.56-12.11) | 0.07(-0.33-0.47) |
| Togolese Republic | 36.71(29.02-45.73) | 3.01(2.39-3.72) | 226.71(160.61-309.32) | 5.82(4.10-7.74) | 93.51(42.77-171.00) | 2.72(2.48-2.97) |
| Tokelau | 0.03(0.02-0.04) | 2.14(1.47-2.80) | 0.03(0.02-0.03) | 1.82(1.37-2.28) | -15.19(-36.88-13.00) | -0.69(-0.75--0.63) |
| Turkmenistan | 556.93(521.77-592.62) | 29.45(27.59-31.34) | 336.56(255.27-432.78) | 8.40(6.42-10.73) | -71.49(-78.36--63.42) | -4.10(-4.66--3.54) |
| Tuvalu | 0.17(0.13-0.21) | 2.58(2.05-3.13) | 0.22(0.17-0.27) | 2.11(1.67-2.63) | -18.09(-37.50-10.07) | -0.72(-0.77--0.67) |
| Ukraine | 2422.73(2268.18-2596.80) | 3.36(3.15-3.59) | 1821.08(1257.95-2535.77) | 2.45(1.68-3.42) | -26.95(-50.74-1.71) | -1.26(-1.50--1.03) |
| Union of the Comoros | 33.19(23.48-43.84) | 16.61(11.98-21.70) | 60.68(44.69-82.22) | 12.50(9.29-16.79) | -24.75(-48.87-7.06) | -1.23(-1.39--1.07) |
| United Arab Emirates | 10.09(7.40-13.84) | 2.38(1.75-3.19) | 62.82(47.41-81.33) | 2.14(1.65-2.67) | -9.83(-32.23-22.06) | 1.11(0.56-1.66) |
| United Kingdom of Great Britain and Northern Ireland | 6609.19(6337.31-6755.53) | 7.30(7.03-7.45) | 9949.15(9232.38-10365.29) | 7.54(7.08-7.83) | 3.36(-0.09-6.27) | 0.01(-0.19-0.22) |
| United Mexican States | 880.99(855.65-897.21) | 2.21(2.13-2.26) | 1796.48(1552.83-2051.36) | 1.43(1.24-1.63) | -35.17(-43.71--26.20) | -1.33(-1.45--1.22) |
| United Republic of Tanzania | 1748.16(1309.50-2185.27) | 16.05(12.16-20.06) | 2877.06(2113.19-3764.24) | 11.36(8.38-14.77) | -29.25(-48.43--5.73) | -1.38(-1.48--1.28) |
| United States of America | 12883.46(12245.16-13191.47) | 4.16(3.97-4.25) | 24328.54(22769.16-25278.69) | 4.20(3.95-4.36) | 1.14(-2.00-3.99) | -0.09(-0.26-0.09) |
| United States Virgin Islands | 2.81(2.27-3.39) | 3.33(2.73-4.00) | 4.06(3.07-5.25) | 2.32(1.77-3.00) | -30.29(-49.00--5.65) | -0.97(-1.13--0.82) |

**ASIR, age-standardized incidence rate; SDI, Socio-demographic index; CI, confidential interval; UI, uncertainty interval.**

**Supplementary Table 5 Age-standardised Deaths of Esophageal Cancer Between 1990 and 2021 at 204 Countries and Territories Level**

|  | **1990** | | **2021** | | **Percentage change 1990-2021**  **ASDR (95%UI)** | **EAPC of**  **ASDR**  **(95% CI)** |
| --- | --- | --- | --- | --- | --- | --- |
|  | **Deaths cases**  **(95%UI)** | **ASDR per 10^5^**  **(95%UI)** | **Deaths cases**  **(95%UI)** | **ASDR per 10^5^**  **(95%UI)** |  |  |
| American Samoa | 0.26(0.22-0.32) | 1.30(1.09-1.55) | 0.74(0.60-0.90) | 1.67(1.38-2.01) | 28.78(-1.69-60.92) | 0.95(0.70-1.21) |
| Antigua and Barbuda | 1.89(1.76-2.02) | 3.51(3.26-3.75) | 3.12(2.92-3.34) | 2.99(2.81-3.19) | -14.63(-21.72--6.66) | -0.48(-0.62--0.34) |
| Arab Republic of Egypt | 317.46(277.55-374.43) | 1.29(1.11-1.57) | 619.02(485.69-783.30) | 1.11(0.88-1.39) | -13.85(-32.92-11.32) | -0.23(-0.37--0.08) |
| Argentine Republic | 2378.49(2256.13-2516.96) | 7.52(7.12-7.96) | 2468.52(2277.81-2672.66) | 4.34(4.01-4.69) | -42.26(-46.97--36.81) | -1.43(-1.58--1.28) |
| Australia | 830.83(781.23-886.42) | 4.24(4.00-4.51) | 1757.22(1574.10-1891.50) | 3.75(3.37-4.03) | -11.58(-19.15--4.16) | -0.14(-0.28--0.00) |
| Barbados | 17.79(16.63-18.96) | 6.08(5.71-6.47) | 27.41(21.60-33.46) | 5.28(4.17-6.44) | -13.28(-32.55-7.69) | -0.68(-0.79--0.57) |
| Belize | 1.90(1.77-2.03) | 2.07(1.93-2.21) | 6.64(5.75-7.57) | 2.24(1.95-2.55) | 8.41(-7.56-24.29) | 0.40(0.10-0.69) |
| Bermuda | 4.49(4.13-4.85) | 7.32(6.74-7.88) | 5.62(4.76-6.70) | 4.06(3.42-4.84) | -44.56(-53.95--32.70) | -2.01(-2.23--1.79) |
| Bolivarian Republic of Venezuela | 247.69(233.61-259.77) | 2.74(2.58-2.87) | 515.07(375.74-684.79) | 1.75(1.28-2.32) | -35.93(-53.04--13.83) | -1.72(-1.83--1.61) |
| Bosnia and Herzegovina | 101.50(86.63-120.09) | 2.45(2.08-2.88) | 137.74(102.70-175.24) | 2.22(1.65-2.82) | -9.41(-33.85-17.85) | -0.62(-0.75--0.49) |
| Brunei Darussalam | 2.99(2.39-3.64) | 3.20(2.55-3.86) | 6.98(5.54-8.49) | 2.15(1.72-2.60) | -33.01(-49.77--10.49) | -0.99(-1.14--0.85) |
| Burkina Faso | 145.26(110.74-180.80) | 3.48(2.70-4.31) | 521.75(361.63-672.29) | 5.87(4.10-7.56) | 68.77(24.39-124.42) | 1.33(1.00-1.66) |
| Canada | 1146.96(1075.72-1215.97) | 3.53(3.31-3.74) | 2611.02(2380.99-2808.69) | 3.55(3.27-3.82) | 0.63(-7.10-9.00) | 0.37(0.24-0.49) |
| Central African Republic | 167.82(117.08-207.28) | 14.66(10.37-17.78) | 252.23(168.62-348.21) | 11.46(7.83-15.35) | -21.82(-42.10-1.57) | -0.57(-0.69--0.45) |
| Commonwealth of Dominica | 3.10(2.67-3.66) | 5.24(4.52-6.19) | 4.39(3.55-5.58) | 5.24(4.24-6.60) | -0.07(-24.03-30.28) | 0.05(-0.06-0.15) |
| Commonwealth of the Bahamas | 10.25(9.42-11.17) | 6.65(6.11-7.25) | 21.97(17.43-27.52) | 5.33(4.25-6.63) | -19.81(-37.46-2.64) | -1.00(-1.26--0.74) |
| Cook Islands | 0.36(0.30-0.44) | 3.17(2.62-3.79) | 0.65(0.51-0.78) | 2.54(2.01-3.06) | -19.68(-40.92-2.98) | -0.72(-0.77--0.67) |
| Czech Republic | 395.15(361.88-433.97) | 2.90(2.65-3.19) | 631.97(542.03-727.15) | 3.02(2.59-3.48) | 4.19(-12.61-23.33) | 0.77(0.56-0.99) |
| Democratic People's Republic of Korea | 1745.44(1252.35-2317.25) | 10.99(8.11-14.31) | 3046.19(2259.72-4013.57) | 9.13(6.80-11.98) | -16.89(-40.61-18.60) | -0.42(-0.48--0.36) |
| Democratic Republic of Sao Tome and Principe | 1.58(1.27-1.96) | 2.49(2.00-3.05) | 5.27(4.02-6.80) | 4.93(3.76-6.26) | 97.93(56.18-158.94) | 2.03(1.78-2.29) |
| Democratic Republic of the Congo | 1551.61(1080.98-2079.24) | 10.37(7.44-13.65) | 2990.85(2015.83-4074.08) | 8.58(5.91-11.65) | -17.26(-41.76-12.46) | -0.78(-0.84--0.72) |
| Democratic Republic of Timor-Leste | 7.47(5.20-9.94) | 2.76(1.99-3.58) | 18.58(13.56-25.68) | 2.23(1.65-3.05) | -19.11(-45.06-24.14) | -0.92(-1.07--0.77) |
| Democratic Socialist Republic of Sri Lanka | 618.30(521.46-743.52) | 6.03(5.10-7.17) | 1121.91(733.19-1528.61) | 4.16(2.76-5.69) | -31.01(-55.66--1.13) | -0.76(-0.93--0.59) |
| Dominican Republic | 71.84(58.49-86.74) | 2.06(1.69-2.48) | 229.32(173.22-294.97) | 2.30(1.74-2.96) | 11.46(-20.76-54.25) | 0.12(-0.13-0.37) |
| Eastern Republic of Uruguay | 351.64(329.75-372.29) | 8.97(8.41-9.49) | 325.25(298.30-352.04) | 5.64(5.19-6.11) | -37.11(-42.29--31.58) | -1.61(-1.74--1.48) |
| Federal Democratic Republic of Ethiopia | 1959.08(1375.40-2517.58) | 10.06(7.10-13.03) | 2208.32(1784.20-2946.89) | 5.38(4.35-7.22) | -46.48(-62.06--26.16) | -2.18(-2.33--2.04) |
| Federal Democratic Republic of Nepal | 474.71(360.71-617.29) | 5.13(3.89-6.62) | 910.38(648.48-1297.60) | 4.04(2.89-5.73) | -21.18(-43.18-9.23) | -1.20(-1.47--0.93) |
| Federal Republic of Germany | 4090.08(3869.38-4313.95) | 3.35(3.16-3.53) | 6948.59(6330.79-7476.90) | 3.69(3.39-3.93) | 10.19(1.51-20.21) | 0.47(0.31-0.64) |
| Federal Republic of Nigeria | 1222.04(937.63-1643.78) | 2.86(2.21-3.80) | 3946.92(2694.96-5274.37) | 4.64(3.22-6.04) | 62.57(17.34-118.19) | 1.63(1.41-1.85) |
| Federal Republic of Somalia | 515.60(354.82-709.38) | 21.52(15.20-28.90) | 940.27(657.18-1263.65) | 15.97(11.10-21.10) | -25.83(-43.16--0.85) | -0.88(-0.98--0.79) |
| Federated States of Micronesia | 1.56(1.23-1.98) | 3.39(2.69-4.24) | 2.00(1.50-2.64) | 2.85(2.22-3.72) | -16.06(-40.35-20.02) | -0.57(-0.61--0.52) |
| Federative Republic of Brazil | 6224.83(5970.09-6409.72) | 7.14(6.77-7.37) | 12869.95(12150.28-13441.01) | 5.09(4.80-5.32) | -28.62(-32.17--25.61) | -0.98(-1.03--0.92) |
| French Republic | 6279.68(5890.82-6663.77) | 7.96(7.48-8.43) | 5108.43(4567.82-5570.62) | 3.65(3.30-3.97) | -54.13(-58.71--49.39) | -2.50(-2.58--2.42) |
| Gabonese Republic | 73.67(55.13-92.46) | 13.19(9.87-16.50) | 113.58(82.20-147.43) | 11.20(8.24-14.38) | -15.08(-36.32-14.80) | -0.35(-0.47--0.22) |
| Georgia | 140.04(121.60-161.25) | 2.23(1.94-2.56) | 67.63(57.23-79.72) | 1.13(0.96-1.33) | -49.30(-58.96--38.02) | -2.31(-2.73--1.90) |
| Grand Duchy of Luxembourg | 26.31(24.85-27.71) | 4.89(4.61-5.14) | 36.23(32.65-39.96) | 3.35(3.02-3.70) | -31.39(-38.36--24.18) | -1.01(-1.12--0.91) |
| Greenland | 5.67(4.85-6.55) | 17.01(14.66-19.72) | 7.78(6.18-9.59) | 11.00(8.74-13.72) | -35.32(-50.85--16.00) | -1.02(-1.13--0.92) |
| Grenada | 4.78(4.30-5.32) | 6.78(6.08-7.55) | 5.66(4.80-6.59) | 4.84(4.12-5.60) | -28.57(-40.91--15.14) | -0.34(-0.65--0.03) |
| Guam | 1.65(1.42-1.91) | 2.49(2.14-2.83) | 3.93(3.29-4.63) | 1.84(1.55-2.17) | -26.22(-41.39--8.61) | -1.05(-1.41--0.70) |
| Hashemite Kingdom of Jordan | 14.79(11.62-18.37) | 1.19(0.94-1.46) | 60.19(46.35-77.38) | 0.88(0.69-1.12) | -25.79(-46.60-3.03) | -1.18(-1.33--1.04) |
| Hellenic Republic | 347.60(325.32-367.29) | 2.27(2.12-2.39) | 399.38(361.97-426.40) | 1.66(1.54-1.76) | -26.63(-31.80--21.67) | -0.84(-1.08--0.60) |
| Hungary | 603.91(560.10-646.85) | 4.26(3.95-4.55) | 582.73(514.58-659.72) | 3.19(2.81-3.60) | -25.15(-33.88--13.67) | -0.06(-0.49-0.36) |
| Independent State of Papua New Guinea | 34.43(23.86-50.28) | 2.08(1.46-3.00) | 78.27(54.61-112.16) | 1.72(1.20-2.43) | -17.69(-47.99-21.37) | -0.65(-0.69--0.61) |
| Independent State of Samoa | 1.09(0.85-1.34) | 1.35(1.06-1.63) | 1.63(1.27-2.07) | 1.17(0.92-1.47) | -13.40(-33.89-18.30) | -0.30(-0.41--0.20) |
| Ireland | 322.67(302.04-345.40) | 7.92(7.40-8.44) | 434.74(381.88-481.48) | 5.39(4.76-5.96) | -31.92(-39.08--24.01) | -0.63(-0.77--0.48) |
| Islamic Republic of Afghanistan | 684.74(333.49-986.62) | 9.91(4.94-14.04) | 733.06(346.77-1099.53) | 7.77(3.85-11.29) | -21.59(-42.87-13.61) | -0.73(-0.84--0.62) |
| Islamic Republic of Iran | 1262.63(1084.94-1413.53) | 5.47(4.65-6.14) | 2869.74(2594.56-3129.26) | 3.99(3.58-4.35) | -27.06(-36.29--15.54) | -0.84(-0.92--0.76) |
| Islamic Republic of Mauritania | 37.01(28.61-47.42) | 3.81(2.95-4.91) | 115.90(82.01-159.49) | 5.62(4.00-7.63) | 47.71(3.66-102.26) | 0.92(0.63-1.22) |
| Islamic Republic of Pakistan | 3635.37(3051.56-4180.59) | 6.60(5.49-7.57) | 7912.71(6283.42-9968.76) | 6.70(5.36-8.40) | 1.54(-19.73-32.53) | 0.42(0.18-0.67) |
| Jamaica | 56.21(52.36-60.48) | 3.12(2.91-3.35) | 97.14(73.50-124.43) | 3.13(2.36-4.01) | 0.07(-24.17-29.28) | -0.51(-0.84--0.19) |
| Japan | 8493.33(8105.25-8716.17) | 4.99(4.73-5.12) | 14565.16(12956.99-15419.32) | 3.81(3.49-3.99) | -23.48(-26.63--21.15) | -0.78(-0.87--0.69) |
| Kingdom of Bahrain | 5.90(4.99-6.88) | 4.22(3.64-4.90) | 15.14(11.90-19.41) | 2.31(1.86-2.86) | -45.19(-57.34--28.99) | -1.94(-2.27--1.61) |
| Kingdom of Belgium | 613.59(572.96-651.92) | 4.06(3.80-4.31) | 961.41(871.28-1046.89) | 4.11(3.77-4.44) | 1.27(-7.76-9.57) | 0.13(0.00-0.25) |
| Kingdom of Bhutan | 12.76(9.16-17.36) | 5.34(3.78-7.22) | 22.61(16.24-31.45) | 3.82(2.76-5.27) | -28.41(-50.11-6.98) | -1.33(-1.47--1.20) |
| Kingdom of Cambodia | 200.98(156.04-252.92) | 4.48(3.46-5.55) | 375.16(275.90-493.91) | 3.12(2.32-4.07) | -30.40(-49.97--4.31) | -1.26(-1.32--1.19) |
| Kingdom of Denmark | 383.46(363.03-406.07) | 4.84(4.59-5.12) | 565.79(508.17-610.69) | 4.70(4.26-5.05) | -2.96(-11.48-5.26) | 0.39(0.18-0.61) |
| Kingdom of Eswatini | 49.28(36.65-62.59) | 17.39(12.95-21.76) | 100.36(70.24-138.63) | 17.47(12.43-23.38) | 0.49(-27.01-42.51) | 0.70(0.30-1.09) |
| Kingdom of Lesotho | 90.58(70.44-116.01) | 10.92(8.51-14.02) | 180.57(133.84-233.70) | 16.67(12.38-21.26) | 52.65(10.49-113.69) | 1.65(1.37-1.94) |
| Kingdom of Morocco | 120.19(93.76-147.41) | 0.87(0.68-1.06) | 301.57(219.11-380.32) | 0.90(0.66-1.12) | 3.62(-24.46-34.32) | -0.22(-0.38--0.07) |
| Kingdom of Norway | 173.62(164.82-180.52) | 2.56(2.45-2.65) | 301.45(277.02-318.42) | 2.92(2.70-3.07) | 13.94(8.28-19.44) | 0.31(0.20-0.43) |
| Kingdom of Saudi Arabia | 124.94(90.01-167.07) | 2.32(1.70-3.07) | 311.48(238.22-418.90) | 1.81(1.43-2.33) | -22.21(-46.01-9.24) | -0.65(-0.81--0.49) |
| Kingdom of Spain | 2199.17(2068.75-2321.85) | 4.15(3.90-4.38) | 2292.10(2054.65-2476.38) | 2.40(2.18-2.60) | -42.06(-46.58--36.76) | -1.53(-1.63--1.42) |
| Kingdom of Sweden | 415.08(385.33-438.52) | 2.69(2.52-2.82) | 596.51(515.77-665.64) | 2.62(2.28-2.93) | -2.30(-14.43-9.75) | 0.08(-0.01-0.18) |
| Kingdom of Thailand | 1776.89(1384.98-2384.70) | 5.03(3.88-6.72) | 4848.53(3619.31-6300.69) | 4.44(3.33-5.75) | -11.84(-39.86-30.83) | -0.74(-0.85--0.63) |
| Kingdom of the Netherlands | 959.19(896.38-1013.32) | 4.82(4.50-5.08) | 2262.73(2042.18-2450.10) | 6.21(5.63-6.69) | 28.96(19.24-38.90) | 1.48(1.25-1.70) |
| Kingdom of Tonga | 1.12(0.90-1.40) | 2.18(1.74-2.71) | 1.65(1.27-2.14) | 2.12(1.63-2.74) | -2.79(-28.59-28.16) | -0.12(-0.24-0.01) |
| Kyrgyz Republic | 259.80(224.73-296.73) | 8.80(7.66-10.00) | 138.03(108.96-168.68) | 3.03(2.44-3.64) | -65.57(-72.33--56.81) | -3.05(-3.16--2.94) |
| Lao People's Democratic Republic | 95.63(68.02-128.87) | 4.60(3.30-6.12) | 118.15(87.13-165.37) | 2.64(1.99-3.63) | -42.64(-62.61--13.58) | -1.84(-1.90--1.78) |
| Lebanese Republic | 28.45(22.30-36.28) | 1.37(1.08-1.73) | 57.74(46.20-70.21) | 0.94(0.75-1.14) | -31.51(-48.89--5.05) | -1.04(-1.17--0.92) |
| Malaysia | 245.67(205.11-285.31) | 2.76(2.32-3.19) | 742.88(632.21-864.99) | 2.69(2.30-3.11) | -2.74(-22.04-20.14) | -0.23(-0.32--0.14) |
| Mongolia | 253.97(208.80-311.91) | 25.54(20.87-31.41) | 359.35(286.62-434.71) | 17.98(14.50-21.49) | -29.60(-45.10--8.21) | -0.82(-1.03--0.61) |
| Montenegro | 12.78(10.50-15.81) | 2.00(1.65-2.48) | 22.13(17.14-28.34) | 2.29(1.78-2.92) | 14.62(-14.88-62.43) | 0.42(0.32-0.52) |
| New Zealand | 190.12(178.04-201.14) | 4.82(4.51-5.11) | 292.65(265.18-316.72) | 3.33(3.03-3.59) | -31.00(-36.08--24.79) | -0.66(-0.90--0.42) |
| North Macedonia | 28.02(24.32-32.69) | 1.50(1.31-1.73) | 46.83(36.10-59.06) | 1.46(1.14-1.81) | -2.68(-25.19-26.99) | 0.59(0.31-0.88) |
| Northern Mariana Islands | 0.22(0.17-0.29) | 1.47(1.20-1.89) | 1.26(1.04-1.49) | 2.71(2.22-3.24) | 84.32(35.10-140.86) | 2.26(1.85-2.67) |
| Palestine | 11.95(8.82-15.46) | 1.50(1.12-1.93) | 21.26(17.19-26.10) | 0.96(0.78-1.18) | -35.79(-53.78--10.89) | -1.22(-1.40--1.04) |
| People's Democratic Republic of Algeria | 86.34(70.34-104.13) | 0.82(0.67-0.97) | 241.11(185.36-302.99) | 0.75(0.59-0.94) | -8.27(-32.73-18.17) | -0.82(-1.08--0.56) |
| People's Republic of Bangladesh | 2549.00(1820.74-3398.44) | 5.44(3.88-7.19) | 4694.63(3318.21-6524.79) | 3.48(2.46-4.82) | -35.91(-55.06--9.93) | -1.37(-1.47--1.27) |
| People's Republic of China | 210820.63(176081.38-244586.52) | 26.06(21.77-30.10) | 296443.04(236647.81-362831.35) | 14.13(11.36-17.18) | -45.78(-58.18--30.43) | -1.88(-2.06--1.70) |
| Plurinational State of Bolivia | 90.81(69.69-116.07) | 3.06(2.35-3.88) | 206.99(152.03-278.10) | 2.45(1.82-3.27) | -19.80(-42.21-14.76) | -0.79(-0.85--0.74) |
| Portuguese Republic | 662.93(621.94-701.91) | 4.88(4.57-5.16) | 744.83(677.09-813.38) | 3.24(2.96-3.52) | -33.63(-39.95--26.84) | -1.17(-1.25--1.10) |
| Principality of Andorra | 1.10(0.76-1.58) | 1.93(1.34-2.75) | 1.84(1.20-2.64) | 1.18(0.76-1.69) | -39.16(-64.83--0.59) | -1.20(-1.37--1.03) |
| Principality of Monaco | 3.47(2.62-4.36) | 4.92(3.75-6.14) | 5.46(4.31-6.66) | 5.48(4.33-6.71) | 11.34(-17.08-52.59) | 0.31(0.14-0.47) |
| Puerto Rico | 230.69(216.30-245.39) | 6.44(6.07-6.83) | 168.91(137.97-198.01) | 2.39(1.95-2.80) | -62.96(-69.83--56.16) | -3.07(-3.19--2.94) |
| Republic of Albania | 40.36(33.70-48.34) | 2.05(1.71-2.45) | 65.48(47.92-85.93) | 1.52(1.11-2.00) | -26.00(-46.58-6.01) | -1.02(-1.19--0.85) |
| Republic of Angola | 477.04(320.51-645.60) | 12.44(8.69-16.54) | 958.73(688.78-1264.14) | 8.58(6.13-11.20) | -31.04(-50.95-2.08) | -1.18(-1.28--1.08) |
| Republic of Armenia | 78.50(72.00-84.96) | 2.98(2.72-3.24) | 48.05(42.14-55.20) | 1.08(0.94-1.24) | -63.88(-69.51--56.99) | -2.98(-3.21--2.75) |
| Republic of Austria | 294.43(278.09-311.59) | 2.57(2.43-2.71) | 416.88(376.30-449.86) | 2.31(2.11-2.48) | -10.13(-17.34--2.18) | -0.12(-0.19--0.04) |
| Republic of Azerbaijan | 437.03(378.24-496.95) | 8.93(7.74-10.24) | 559.28(411.62-718.14) | 5.79(4.25-7.43) | -35.20(-53.11--15.87) | -1.25(-1.37--1.12) |
| Republic of Belarus | 382.81(352.09-414.03) | 2.91(2.67-3.16) | 461.15(373.16-573.10) | 2.86(2.32-3.55) | -1.73(-21.59-23.82) | 0.51(0.18-0.83) |
| Republic of Benin | 65.20(54.47-78.77) | 3.37(2.81-4.09) | 267.75(200.98-348.75) | 5.41(4.05-7.02) | 60.33(18.72-115.14) | 1.33(1.06-1.61) |
| Republic of Botswana | 69.29(49.79-92.74) | 12.84(9.28-16.85) | 129.28(96.24-167.83) | 9.22(7.01-11.95) | -28.14(-49.65-1.84) | -0.80(-1.01--0.59) |
| Republic of Bulgaria | 317.71(295.76-343.25) | 2.63(2.46-2.83) | 306.73(263.27-351.88) | 2.30(1.98-2.63) | -12.54(-25.81-2.61) | 0.23(-0.11-0.57) |
| Republic of Burundi | 463.14(334.14-581.36) | 20.27(14.65-25.34) | 533.56(383.55-692.67) | 11.50(8.29-14.73) | -43.27(-59.00--20.65) | -1.83(-2.06--1.60) |
| Republic of Cabo Verde | 21.74(17.70-25.70) | 9.39(7.75-11.11) | 69.65(55.08-85.79) | 15.93(12.58-19.70) | 69.66(29.60-122.00) | 0.85(0.58-1.11) |
| Republic of Cameroon | 162.70(124.23-205.23) | 3.80(2.97-4.74) | 822.35(559.91-1140.37) | 6.71(4.67-9.12) | 76.53(26.37-146.91) | 1.84(1.63-2.04) |
| Republic of Chad | 75.06(60.79-90.92) | 2.73(2.22-3.30) | 326.84(242.77-427.55) | 5.85(4.39-7.49) | 114.76(59.29-196.01) | 2.30(2.03-2.58) |
| Republic of Chile | 769.96(726.29-810.60) | 8.12(7.64-8.55) | 833.11(750.31-899.88) | 3.18(2.87-3.44) | -60.77(-63.92--57.23) | -2.52(-2.75--2.28) |
| Republic of Colombia | 728.21(687.75-765.82) | 4.45(4.18-4.69) | 1040.54(861.26-1238.59) | 1.88(1.56-2.25) | -57.65(-64.67--49.12) | -3.09(-3.22--2.96) |
| Republic of Costa Rica | 44.74(41.34-47.88) | 2.65(2.44-2.84) | 89.80(78.23-100.77) | 1.63(1.42-1.83) | -38.49(-46.04--30.83) | -1.43(-1.59--1.28) |
| Republic of Croatia | 235.03(218.24-251.77) | 3.81(3.56-4.08) | 221.66(192.72-250.47) | 2.58(2.24-2.91) | -32.40(-42.40--22.97) | -0.94(-1.09--0.78) |
| Republic of Cuba | 398.68(377.68-422.10) | 3.95(3.74-4.17) | 979.57(830.96-1134.88) | 4.95(4.20-5.75) | 25.43(7.64-46.73) | 0.52(0.30-0.73) |
| Republic of Cyprus | 10.86(8.84-13.37) | 1.56(1.26-1.90) | 27.69(21.70-35.33) | 1.37(1.08-1.72) | -12.18(-34.53-17.57) | -0.23(-0.42--0.04) |
| Republic of Cote d'Ivoire | 52.51(41.80-63.68) | 1.39(1.15-1.65) | 149.59(110.69-201.60) | 1.39(1.06-1.84) | 0.29(-24.29-38.48) | 0.35(0.21-0.50) |
| Republic of Djibouti | 20.75(14.67-28.81) | 15.76(11.49-21.07) | 79.23(52.43-115.38) | 13.06(8.89-18.52) | -17.15(-42.24-19.93) | -0.40(-0.53--0.27) |
| Republic of Ecuador | 112.32(105.00-118.92) | 2.27(2.11-2.41) | 209.24(166.10-260.95) | 1.32(1.05-1.64) | -41.57(-53.89--27.18) | -1.54(-1.72--1.36) |
| Republic of El Salvador | 57.96(51.50-64.80) | 2.00(1.77-2.24) | 112.17(89.28-140.69) | 1.78(1.42-2.24) | -10.74(-31.08-13.00) | -0.78(-0.99--0.57) |
| Republic of Equatorial Guinea | 26.99(18.74-35.54) | 13.97(10.01-18.16) | 45.03(30.68-65.49) | 9.42(6.55-13.41) | -32.53(-54.93--1.39) | -1.42(-1.63--1.21) |
| Republic of Estonia | 69.52(64.75-74.54) | 3.38(3.15-3.62) | 66.72(57.32-76.71) | 2.58(2.21-2.99) | -23.48(-35.86--9.10) | -0.24(-0.57-0.09) |
| Republic of Fiji | 7.89(6.25-9.92) | 2.46(1.97-3.07) | 19.35(13.72-25.21) | 2.86(2.01-3.72) | 16.23(-19.51-59.99) | 0.58(0.48-0.68) |
| Republic of Finland | 238.13(223.39-251.39) | 3.34(3.14-3.53) | 361.02(326.80-388.58) | 2.82(2.58-3.02) | -15.65(-21.77--9.44) | -0.89(-1.03--0.75) |
| Republic of Ghana | 155.42(119.61-198.02) | 2.59(2.01-3.26) | 594.54(404.61-772.26) | 3.80(2.53-4.97) | 46.39(1.38-97.78) | 1.26(1.09-1.44) |
| Republic of Guatemala | 84.56(81.09-88.00) | 2.89(2.77-3.01) | 190.83(163.15-223.40) | 1.81(1.56-2.12) | -37.26(-46.18--27.01) | -1.30(-1.52--1.09) |
| Republic of Guinea | 41.87(32.93-51.59) | 1.29(1.01-1.58) | 78.83(55.95-106.67) | 1.44(1.03-1.94) | 11.98(-27.25-66.22) | 0.42(0.30-0.53) |
| Republic of Guinea-Bissau | 19.64(14.62-24.73) | 5.01(3.78-6.23) | 55.25(41.13-69.56) | 7.80(5.81-9.72) | 55.72(16.09-116.49) | 1.23(0.94-1.52) |
| Republic of Guyana | 8.28(7.38-9.23) | 2.24(2.00-2.48) | 13.97(10.58-17.93) | 2.15(1.63-2.74) | -4.24(-28.99-23.67) | -0.24(-0.42--0.07) |
| Republic of Haiti | 175.32(125.48-228.13) | 5.63(4.12-7.21) | 294.13(205.80-414.91) | 4.26(3.02-5.88) | -24.39(-45.95-2.73) | -0.89(-0.97--0.81) |
| Republic of Honduras | 21.24(17.52-25.22) | 1.10(0.91-1.30) | 93.75(73.63-118.91) | 1.60(1.26-2.00) | 45.69(11.06-92.21) | 1.56(1.44-1.68) |
| Republic of Iceland | 12.95(11.98-13.80) | 4.53(4.19-4.82) | 22.69(19.96-25.23) | 3.85(3.41-4.27) | -15.05(-23.89--4.38) | -0.37(-0.49--0.26) |
| Republic of India | 17155.50(14894.89-21464.34) | 3.68(3.18-4.65) | 38002.11(33296.32-45561.82) | 3.23(2.83-3.89) | -12.43(-24.12-3.49) | -0.45(-0.55--0.36) |
| Republic of Indonesia | 1881.72(1400.85-2288.61) | 1.95(1.47-2.39) | 3889.04(2981.00-4884.69) | 1.71(1.32-2.14) | -12.21(-30.98-10.71) | -0.23(-0.29--0.16) |
| Republic of Iraq | 97.03(76.09-121.92) | 1.25(0.98-1.56) | 273.94(198.01-351.98) | 1.23(0.92-1.56) | -1.26(-32.46-39.28) | -0.11(-0.25-0.03) |
| Republic of Italy | 2920.05(2765.44-3019.37) | 3.32(3.15-3.43) | 2395.33(2149.03-2539.89) | 1.61(1.48-1.69) | -51.58(-53.64--49.47) | -2.29(-2.35--2.23) |
| Republic of Kazakhstan | 2490.02(2308.55-2686.50) | 20.32(18.80-21.94) | 868.04(747.05-1004.88) | 5.02(4.34-5.80) | -75.29(-78.89--71.06) | -3.82(-4.10--3.54) |
| Republic of Kenya | 765.21(552.27-1102.20) | 9.58(6.90-13.92) | 2736.43(1989.02-3846.52) | 12.69(9.20-17.97) | 32.42(0.61-75.99) | 1.05(0.89-1.21) |
| Republic of Kiribati | 2.30(1.81-2.85) | 6.57(5.18-8.20) | 4.02(2.89-5.26) | 5.96(4.23-7.64) | -9.20(-35.12-23.48) | -0.18(-0.27--0.09) |
| Republic of Korea | 1716.26(1389.77-2075.16) | 5.80(4.72-6.98) | 2195.94(1757.30-2698.35) | 2.29(1.83-2.81) | -60.53(-70.49--46.27) | -3.10(-3.28--2.92) |
| Republic of Latvia | 113.04(105.11-121.48) | 3.13(2.91-3.36) | 107.65(92.83-123.10) | 2.90(2.49-3.33) | -7.36(-21.04-7.43) | 0.19(0.02-0.37) |
| Republic of Liberia | 37.16(29.31-45.91) | 3.33(2.66-4.07) | 123.32(84.93-175.81) | 6.07(4.24-8.44) | 82.33(28.28-153.86) | 1.64(1.31-1.98) |
| Republic of Lithuania | 146.48(137.11-156.94) | 3.22(3.02-3.44) | 191.42(163.96-219.39) | 3.56(3.06-4.08) | 10.71(-5.93-27.02) | 1.06(0.82-1.30) |
| Republic of Madagascar | 768.90(565.62-933.78) | 15.40(11.33-18.62) | 1256.68(862.14-1726.80) | 11.59(7.93-15.79) | -24.73(-46.33-0.19) | -0.84(-0.95--0.73) |
| Republic of Malawi | 837.84(689.78-1010.05) | 22.60(18.55-27.08) | 2017.24(1625.49-2540.78) | 27.77(22.45-34.72) | 22.92(-5.39-60.00) | 0.91(0.68-1.15) |
| Republic of Maldives | 2.86(2.03-3.68) | 3.20(2.38-4.06) | 3.90(3.06-4.83) | 1.22(0.97-1.49) | -61.87(-71.40--45.25) | -3.12(-3.31--2.93) |
| Republic of Mali | 99.83(83.94-117.53) | 2.59(2.20-3.03) | 222.86(168.65-288.98) | 2.63(2.02-3.39) | 1.54(-28.70-34.80) | -0.04(-0.19-0.10) |
| Republic of Malta | 12.41(11.46-13.42) | 2.92(2.70-3.15) | 20.88(18.35-23.24) | 2.17(1.93-2.41) | -25.71(-34.36--15.91) | -1.03(-1.14--0.93) |
| Republic of Mauritius | 26.55(25.19-27.91) | 3.76(3.56-3.96) | 65.02(60.16-68.79) | 3.51(3.26-3.70) | -6.84(-14.78-0.51) | -0.54(-0.83--0.24) |
| Republic of Moldova | 112.95(106.25-119.27) | 2.56(2.41-2.70) | 111.09(99.25-124.37) | 1.84(1.65-2.05) | -28.14(-36.28--17.88) | -0.61(-0.93--0.30) |
| Republic of Mozambique | 448.07(356.64-550.51) | 8.35(6.68-10.10) | 993.47(749.23-1247.91) | 9.79(7.56-12.22) | 17.22(-15.02-53.40) | 0.80(0.68-0.92) |
| Republic of Namibia | 15.96(13.18-19.41) | 2.49(2.07-3.00) | 34.37(26.35-45.76) | 2.46(1.93-3.19) | -0.97(-26.96-35.14) | 0.11(-0.11-0.33) |
| Republic of Nauru | 0.19(0.14-0.25) | 4.27(3.19-5.50) | 0.19(0.14-0.25) | 3.44(2.58-4.38) | -19.55(-41.01-9.14) | -0.59(-0.72--0.47) |
| Republic of Nicaragua | 16.07(13.88-18.07) | 1.12(0.97-1.27) | 41.48(33.53-51.25) | 0.89(0.72-1.09) | -21.02(-36.97--1.03) | -1.02(-1.22--0.83) |
| Republic of Niue | 0.06(0.04-0.07) | 2.48(1.99-3.01) | 0.05(0.04-0.07) | 2.54(1.99-3.10) | 2.66(-22.75-34.35) | 0.21(0.10-0.33) |
| Republic of Palau | 0.29(0.23-0.37) | 3.45(2.78-4.34) | 0.59(0.47-0.75) | 3.13(2.55-3.88) | -9.26(-33.06-22.56) | -0.28(-0.31--0.25) |
| Republic of Panama | 28.70(26.66-30.73) | 2.02(1.87-2.16) | 57.04(44.50-68.85) | 1.28(1.00-1.55) | -36.47(-50.05--23.77) | -1.37(-1.47--1.26) |
| Republic of Paraguay | 72.44(60.16-87.48) | 3.35(2.77-4.06) | 243.02(180.45-325.08) | 4.22(3.14-5.65) | 25.88(-12.67-74.63) | 0.83(0.73-0.93) |
| Republic of Peru | 215.35(177.76-261.03) | 1.91(1.58-2.29) | 450.06(338.17-591.33) | 1.36(1.02-1.79) | -28.52(-49.04-0.53) | -1.19(-1.37--1.00) |
| Republic of Poland | 1632.87(1571.03-1685.78) | 3.78(3.63-3.91) | 2201.00(1994.92-2398.60) | 3.14(2.84-3.42) | -17.13(-24.97--9.66) | -0.55(-0.69--0.41) |
| Republic of Rwanda | 628.37(428.57-793.79) | 22.38(15.44-28.06) | 724.22(526.65-968.68) | 12.22(9.01-16.33) | -45.41(-61.79--21.69) | -1.87(-2.27--1.48) |
| Republic of San Marino | 0.56(0.44-0.70) | 1.61(1.27-1.99) | 0.64(0.41-0.92) | 0.85(0.53-1.25) | -47.22(-67.49--15.83) | -1.14(-1.36--0.91) |
| Republic of Senegal | 101.85(82.41-122.54) | 3.25(2.64-3.89) | 407.00(307.19-528.64) | 5.46(4.09-7.05) | 68.01(26.84-123.58) | 1.41(1.08-1.74) |
| Republic of Serbia | 278.44(208.66-377.16) | 2.52(1.92-3.36) | 349.61(255.19-477.50) | 2.17(1.57-2.98) | -14.04(-41.95-28.67) | -0.33(-0.45--0.22) |
| Republic of Seychelles | 3.13(2.65-3.68) | 5.59(4.73-6.56) | 5.63(4.54-6.87) | 4.77(3.88-5.76) | -14.70(-32.37-11.10) | 0.34(0.10-0.59) |
| Republic of Sierra Leone | 59.58(46.43-73.86) | 3.00(2.35-3.69) | 189.12(136.36-254.19) | 5.19(3.75-6.89) | 73.19(27.31-132.91) | 1.50(1.15-1.85) |
| Republic of Singapore | 91.08(85.71-96.71) | 4.30(4.05-4.57) | 146.12(131.19-157.81) | 1.71(1.53-1.84) | -60.32(-64.24--56.33) | -3.38(-3.64--3.12) |
| Republic of Slovenia | 85.81(80.49-91.37) | 3.43(3.22-3.65) | 96.42(82.87-109.17) | 2.21(1.88-2.49) | -35.71(-45.49--26.67) | -1.36(-1.54--1.18) |
| Republic of South Africa | 2439.09(2149.81-2917.86) | 11.75(10.35-14.28) | 5008.86(4543.59-5609.08) | 11.06(10.06-12.30) | -5.87(-22.17-8.81) | -0.08(-0.44-0.28) |
| Republic of South Sudan | 469.36(347.39-639.60) | 18.71(13.92-25.25) | 614.05(442.95-820.35) | 16.21(11.98-21.42) | -13.35(-35.51-21.73) | -0.54(-0.67--0.42) |
| Republic of Sudan | 459.46(288.00-617.26) | 5.10(3.20-6.88) | 838.96(526.25-1188.55) | 4.56(2.97-6.31) | -10.50(-38.04-28.33) | -0.43(-0.46--0.40) |
| Republic of Suriname | 4.67(4.03-5.39) | 1.86(1.61-2.15) | 10.85(8.10-13.96) | 1.69(1.26-2.16) | -9.39(-36.49-22.21) | -0.27(-0.45--0.10) |
| Republic of Tajikistan | 327.47(269.03-392.16) | 12.34(10.23-14.66) | 345.95(257.19-445.25) | 6.77(5.07-8.66) | -45.14(-60.54--23.86) | -1.61(-1.79--1.43) |
| Republic of the Congo | 174.33(131.08-229.07) | 16.70(12.72-21.64) | 292.90(206.55-398.23) | 11.26(8.25-14.92) | -32.56(-50.58--10.07) | -1.20(-1.34--1.06) |
| Republic of the Gambia | 5.39(4.12-6.81) | 1.60(1.25-2.02) | 19.48(14.78-24.83) | 2.07(1.58-2.62) | 28.75(-9.15-71.98) | 0.96(0.86-1.06) |
| Republic of the Marshall Islands | 0.45(0.31-0.60) | 3.00(2.11-3.88) | 0.87(0.64-1.18) | 2.67(2.03-3.54) | -11.02(-36.17-25.66) | -0.03(-0.13-0.07) |
| Republic of the Niger | 79.41(59.47-100.17) | 3.01(2.30-3.76) | 344.67(236.11-458.69) | 4.48(3.08-5.89) | 48.47(6.90-108.09) | 0.84(0.48-1.19) |
| Republic of the Philippines | 422.68(372.00-492.13) | 1.46(1.29-1.69) | 1140.50(931.38-1364.36) | 1.39(1.15-1.66) | -4.70(-22.71-16.52) | -0.07(-0.15-0.01) |
| Republic of the Union of Myanmar | 961.23(722.62-1234.49) | 4.14(3.17-5.27) | 1133.80(869.06-1546.74) | 2.38(1.83-3.22) | -42.63(-60.46--15.39) | -1.77(-1.90--1.64) |
| Republic of Trinidad and Tobago | 20.27(19.14-21.46) | 2.51(2.37-2.65) | 38.92(29.91-49.44) | 2.01(1.55-2.54) | -20.08(-38.72-2.26) | -1.27(-1.49--1.06) |
| Republic of Tunisia | 33.63(26.88-41.44) | 0.71(0.57-0.88) | 89.37(61.67-124.90) | 0.69(0.48-0.96) | -3.85(-36.03-36.66) | -0.45(-0.58--0.31) |
| Republic of Turkey | 855.14(695.81-1026.83) | 2.56(2.11-3.08) | 1526.11(1155.48-1932.19) | 1.67(1.28-2.12) | -34.59(-51.89--14.75) | -1.22(-1.41--1.04) |
| Republic of Uganda | 965.80(764.58-1189.61) | 15.62(12.53-19.03) | 2252.80(1721.14-2971.59) | 15.90(12.41-20.93) | 1.80(-29.54-43.18) | 0.14(-0.06-0.35) |
| Republic of Uzbekistan | 1708.50(1546.05-1869.89) | 15.10(13.64-16.50) | 998.97(826.44-1200.85) | 3.98(3.31-4.76) | -73.66(-78.20--68.69) | -4.52(-4.92--4.13) |
| Republic of Vanuatu | 1.54(1.13-2.09) | 2.67(2.01-3.51) | 3.85(3.00-4.98) | 2.39(1.88-3.05) | -10.58(-38.69-22.24) | -0.40(-0.43--0.36) |
| Republic of Yemen | 251.34(144.86-358.14) | 5.37(3.05-7.71) | 546.18(283.16-783.44) | 4.16(2.19-5.92) | -22.62(-47.99-17.77) | -0.96(-1.03--0.90) |
| Republic of Zambia | 575.68(441.99-711.53) | 20.60(16.02-25.52) | 1159.46(770.04-1800.82) | 17.13(11.78-26.09) | -16.87(-42.44-30.35) | -0.21(-0.48-0.07) |
| Republic of Zimbabwe | 524.72(420.81-629.85) | 13.54(10.92-16.14) | 1148.38(870.62-1443.91) | 17.02(13.23-21.11) | 25.74(-8.19-65.92) | 0.89(0.61-1.17) |
| Romania | 433.10(408.71-461.62) | 1.56(1.48-1.66) | 878.69(774.86-991.06) | 2.54(2.24-2.89) | 62.84(42.00-86.00) | 1.64(1.42-1.87) |
| Russian Federation | 9346.64(9150.25-9516.03) | 5.14(5.02-5.23) | 7709.34(7022.19-8377.31) | 3.22(2.93-3.49) | -37.43(-43.06--32.28) | -1.46(-1.62--1.31) |
| Saint Kitts and Nevis | 1.76(1.64-1.86) | 4.78(4.48-5.06) | 2.77(2.29-3.26) | 4.03(3.35-4.66) | -15.69(-29.96--0.30) | -0.75(-0.99--0.51) |
| Saint Lucia | 4.61(4.36-4.91) | 5.54(5.23-5.89) | 10.57(8.64-12.69) | 4.38(3.59-5.25) | -20.89(-35.26--4.32) | -1.07(-1.26--0.88) |
| Saint Vincent and the Grenadines | 1.73(1.59-1.87) | 2.47(2.26-2.67) | 3.72(3.26-4.22) | 2.62(2.30-2.97) | 5.78(-9.08-23.14) | -0.04(-0.23-0.16) |
| Slovak Republic | 245.06(192.88-315.67) | 4.14(3.27-5.32) | 298.98(231.12-393.61) | 3.21(2.47-4.27) | -22.49(-47.05-12.02) | -0.24(-0.42--0.05) |
| Socialist Republic of Viet Nam | 854.78(641.50-1119.69) | 2.13(1.61-2.78) | 2344.36(1757.11-2997.04) | 2.30(1.75-2.90) | 7.68(-23.14-57.39) | 0.19(0.11-0.27) |
| Solomon Islands | 3.75(2.48-5.08) | 2.96(2.16-3.85) | 8.44(5.69-11.80) | 2.58(1.81-3.55) | -12.88(-40.34-30.07) | -0.36(-0.42--0.31) |
| State of Eritrea | 247.70(161.70-327.83) | 21.36(14.31-27.90) | 410.00(282.83-594.24) | 15.60(10.93-22.48) | -26.96(-44.39--4.78) | -0.81(-0.92--0.70) |
| State of Israel | 100.19(92.77-107.52) | 2.10(1.94-2.25) | 185.24(165.06-200.67) | 1.47(1.32-1.58) | -30.27(-36.54--23.74) | -1.09(-1.22--0.96) |
| State of Kuwait | 10.32(9.48-11.20) | 1.86(1.69-2.03) | 24.06(19.50-29.69) | 0.95(0.76-1.17) | -49.15(-58.69--37.71) | -2.43(-2.84--2.02) |
| State of Libya | 27.00(19.71-36.29) | 1.48(1.08-1.97) | 90.38(63.06-120.96) | 1.76(1.25-2.33) | 19.32(-22.22-83.97) | 0.62(0.50-0.74) |
| State of Qatar | 4.46(3.51-5.57) | 5.53(4.40-6.84) | 18.02(12.97-24.59) | 2.65(1.98-3.51) | -52.21(-67.01--30.34) | -1.72(-2.23--1.21) |
| Sultanate of Oman | 12.94(9.30-17.25) | 2.03(1.48-2.71) | 28.91(21.26-37.24) | 1.62(1.22-2.04) | -20.06(-46.09-18.88) | -0.31(-0.41--0.21) |
| Swiss Confederation | 449.99(422.26-475.41) | 4.38(4.14-4.62) | 531.79(467.24-577.94) | 2.84(2.54-3.07) | -35.24(-41.11--29.86) | -1.49(-1.66--1.33) |
| Syrian Arab Republic | 44.97(35.06-56.17) | 0.92(0.73-1.14) | 111.53(80.84-144.86) | 0.95(0.72-1.20) | 3.59(-27.92-49.49) | 0.55(0.32-0.79) |
| Taiwan (Province of China) | 1407.23(1335.82-1491.32) | 8.90(8.44-9.40) | 3092.54(2777.99-3356.42) | 7.45(6.72-8.07) | -16.20(-25.63--8.43) | -0.11(-0.37-0.14) |
| Togolese Republic | 38.12(30.26-47.35) | 3.23(2.59-3.97) | 232.34(164.48-314.80) | 6.22(4.33-8.23) | 92.53(41.16-168.54) | 2.00(1.74-2.25) |
| Tokelau | 0.03(0.02-0.04) | 2.34(1.61-3.04) | 0.03(0.02-0.04) | 1.94(1.46-2.42) | -17.20(-38.39-9.16) | -0.66(-0.70--0.62) |
| Turkmenistan | 584.17(546.84-621.28) | 31.58(29.53-33.60) | 349.96(268.11-451.05) | 8.98(6.94-11.48) | -71.57(-78.12--63.77) | -4.43(-4.79--4.07) |
| Tuvalu | 0.18(0.14-0.22) | 2.79(2.22-3.39) | 0.22(0.17-0.28) | 2.26(1.78-2.83) | -19.03(-37.30-8.36) | -0.71(-0.75--0.68) |
| Ukraine | 2298.27(2156.49-2463.85) | 3.18(2.99-3.40) | 1657.73(1139.26-2299.26) | 2.21(1.52-3.08) | -30.48(-52.90--3.93) | -0.49(-0.81--0.16) |
| Union of the Comoros | 34.28(24.28-45.58) | 17.74(12.84-23.24) | 63.44(46.62-85.34) | 13.47(10.01-18.11) | -24.08(-47.75-7.10) | -1.02(-1.12--0.92) |
| United Arab Emirates | 10.09(7.49-13.73) | 2.56(1.90-3.40) | 59.83(44.36-77.68) | 2.36(1.85-2.93) | -7.82(-29.84-23.32) | 1.18(0.85-1.51) |
| United Kingdom of Great Britain and Northern Ireland | 6748.03(6456.21-6906.75) | 7.35(7.06-7.51) | 9745.53(8990.11-10170.22) | 7.16(6.69-7.44) | -2.57(-6.04-0.14) | 0.48(0.25-0.71) |
| United Mexican States | 950.72(923.05-969.42) | 2.48(2.38-2.53) | 1888.10(1639.74-2150.23) | 1.53(1.33-1.74) | -38.21(-46.09--29.91) | -1.60(-1.69--1.51) |
| United Republic of Tanzania | 1815.31(1351.98-2262.65) | 17.19(12.96-21.35) | 2994.51(2201.54-3927.97) | 12.24(9.12-16.06) | -28.80(-47.32--4.18) | -1.03(-1.14--0.92) |
| United States of America | 11773.69(11160.60-12076.60) | 3.75(3.57-3.84) | 21340.66(19926.58-22215.89) | 3.62(3.40-3.76) | -3.52(-6.52--0.90) | 0.05(-0.07-0.17) |
| United States Virgin Islands | 2.88(2.34-3.49) | 3.55(2.92-4.25) | 4.29(3.25-5.50) | 2.42(1.86-3.09) | -31.71(-49.46--8.03) | -1.15(-1.26--1.05) |

**ASDR, age-standardized deaths rate; SDI, Socio-demographic index; CI, confidential interval; UI, uncertainty interval.**

**Supplementary Table 6 Age-standardised DALYs of Esophageal Cancer Between 1990 and 2021 at 204 Countries and Territories Level**

|  | **1990** | | | **2021** | | **Percentage change 1990-2021**  **DALYs (95%UI)** | **EAPC of**  **DALYs**  **(95% CI)** |
| --- | --- | --- | --- | --- | --- | --- | --- |
|  | **DALYs cases**  **(95%UI)** | | **DALYs per 10^5^**  **(95%UI)** | **DALYs cases**  **(95%UI)** | **DALYs per 10^5^**  **(95%UI)** |  |  |
| American Samoa | 7.56(6.26-9.22) | 30.78(25.63-37.44) | | 19.92(15.85-24.49) | 39.92(32.07-48.77) | 29.72(-3.29-65.88) | 1.36(0.98-1.74) |
| Antigua and Barbuda | 42.59(39.66-45.49) | 83.79(78.07-89.39) | | 75.56(70.27-81.34) | 68.17(63.54-73.05) | -18.64(-25.89--10.60) | -0.51(-0.74--0.28) |
| Arab Republic of Egypt | 9419.33(8248.71-10874.43) | 31.36(27.39-36.69) | | 17043.20(13289.97-21442.89) | 25.33(19.97-31.93) | -19.23(-38.46-4.82) | -0.77(-0.92--0.62) |
| Argentine Republic | 56905.32(53872.79-60083.91) | 175.58(166.22-185.25) | | 53732.92(49964.13-58118.79) | 97.43(90.62-105.34) | -44.51(-49.12--39.39) | -1.82(-2.06--1.58) |
| Australia | 18924.47(17861.20-20075.07) | 97.45(91.92-103.33) | | 35246.54(32133.15-37878.42) | 82.27(75.67-88.24) | -15.58(-22.64--8.23) | -0.61(-0.70--0.52) |
| Barbados | 393.38(370.52-419.40) | 146.57(137.37-156.58) | | 621.75(479.39-768.90) | 123.56(95.66-153.38) | -15.70(-35.71-6.37) | -0.44(-0.55--0.33) |
| Belize | 48.27(45.14-51.73) | 51.42(48.05-55.10) | | 183.86(157.58-209.94) | 56.64(48.89-64.71) | 10.16(-6.03-27.36) | 0.52(0.03-1.00) |
| Bermuda | 111.86(102.88-121.30) | 176.39(162.35-190.78) | | 121.36(101.11-144.90) | 95.35(79.29-114.35) | -45.94(-56.38--34.06) | -1.64(-1.95--1.33) |
| Bolivarian Republic of Venezuela | 6197.29(5889.26-6501.36) | 62.42(59.10-65.51) | | 12625.34(8928.06-16988.74) | 41.29(29.45-55.37) | -33.85(-53.29--9.91) | -1.47(-1.61--1.33) |
| Bosnia and Herzegovina | 2864.23(2443.35-3396.66) | 63.47(54.41-74.97) | | 3362.01(2481.82-4283.24) | 56.57(41.56-71.86) | -10.88(-34.85-17.39) | -0.36(-0.52--0.21) |
| Brunei Darussalam | 79.22(63.30-97.40) | 75.66(59.98-92.82) | | 191.91(152.19-235.41) | 50.19(40.09-61.35) | -33.67(-51.12--10.91) | -1.00(-1.20--0.80) |
| Burkina Faso | 3946.40(2982.19-4954.58) | 86.25(65.40-107.78) | | 14271.05(9911.97-18427.52) | 143.94(100.00-185.19) | 66.89(21.15-123.62) | 2.30(2.05-2.56) |
| Canada | 26659.88(25185.80-28170.05) | 83.43(78.87-88.06) | | 55597.12(51416.83-59559.04) | 82.27(76.51-88.31) | -1.39(-9.18-6.61) | 0.03(-0.11-0.16) |
| Central African Republic | 5168.62(3553.17-6451.58) | 392.40(274.19-483.74) | | 7962.67(5243.36-11168.82) | 299.91(203.48-413.70) | -23.57(-43.52-1.92) | -1.04(-1.14--0.93) |
| Commonwealth of Dominica | 71.04(60.66-84.05) | 122.57(104.64-144.72) | | 110.20(87.12-144.51) | 127.81(101.27-167.10) | 4.28(-21.45-38.91) | 0.32(0.12-0.51) |
| Commonwealth of the Bahamas | 295.03(269.48-323.78) | 179.95(164.40-196.85) | | 618.86(487.34-781.77) | 139.84(110.50-175.88) | -22.29(-40.27-1.13) | -0.35(-0.60--0.10) |
| Cook Islands | 9.43(7.58-11.52) | 73.56(59.53-89.23) | | 15.14(11.71-18.71) | 59.74(46.20-74.30) | -18.79(-40.69-7.84) | -0.64(-0.72--0.57) |
| Czech Republic | 10312.25(9450.24-11267.15) | 77.90(71.25-85.33) | | 14920.43(12685.12-17271.52) | 77.31(65.69-89.43) | -0.76(-16.55-17.83) | -0.10(-0.23-0.03) |
| Democratic People's Republic of Korea | 50293.31(35683.15-68122.92) | 283.37(203.89-378.62) | | 83332.08(60440.99-111995.91) | 241.08(176.36-322.41) | -14.92(-41.32-24.17) | -0.49(-0.58--0.41) |
| Democratic Republic of Sao Tome and Principe | 40.80(31.84-51.45) | 61.40(48.23-76.95) | | 145.85(108.97-194.50) | 120.42(91.75-156.99) | 96.13(50.92-164.72) | 2.67(2.50-2.84) |
| Democratic Republic of the Congo | 45473.52(31044.14-61627.79) | 262.82(183.46-350.30) | | 87852.43(58112.92-121143.97) | 213.24(143.54-291.29) | -18.86(-43.05-14.02) | -0.78(-0.89--0.68) |
| Democratic Republic of Timor-Leste | 234.16(159.45-318.37) | 70.60(49.61-93.55) | | 490.85(349.83-691.43) | 54.95(39.45-76.40) | -22.16(-49.25-18.08) | -0.82(-1.08--0.55) |
| Democratic Socialist Republic of Sri Lanka | 17175.75(14415.40-20695.32) | 148.16(125.13-177.69) | | 28218.89(18168.27-39075.13) | 101.75(65.82-140.49) | -31.33(-57.49-0.61) | -0.86(-1.15--0.57) |
| Dominican Republic | 1952.05(1584.53-2373.64) | 49.95(40.42-60.75) | | 6007.98(4529.46-7753.03) | 58.46(43.94-75.31) | 17.06(-18.94-59.06) | 1.05(0.86-1.24) |
| Eastern Republic of Uruguay | 7944.83(7461.50-8441.23) | 206.97(194.57-220.05) | | 6618.19(6079.51-7188.04) | 126.68(116.38-136.86) | -38.79(-44.42--33.38) | -1.80(-2.03--1.56) |
| Federal Democratic Republic of Ethiopia | 58050.17(40275.78-74818.46) | 262.78(183.93-339.39) | | 60062.97(47898.69-80044.00) | 129.60(104.33-172.95) | -50.68(-65.53--29.31) | -2.74(-2.94--2.54) |
| Federal Democratic Republic of Nepal | 14211.58(10736.46-18714.43) | 135.13(102.53-176.70) | | 24539.28(17263.28-35170.26) | 100.19(70.67-142.84) | -25.86(-47.84-3.56) | -0.84(-1.24--0.44) |
| Federal Republic of Germany | 107035.05(100726.23-113370.11) | 92.32(86.78-98.16) | | 152265.15(140344.67-162754.48) | 90.66(84.22-96.82) | -1.80(-10.36-7.48) | -0.38(-0.57--0.18) |
| Federal Republic of Nigeria | 33104.36(25128.94-45424.15) | 70.90(54.16-96.33) | | 107905.02(72624.31-147643.87) | 108.92(74.14-146.09) | 53.62(8.38-112.64) | 1.96(1.72-2.19) |
| Federal Republic of Somalia | 16532.13(11199.91-23158.53) | 575.46(400.10-782.86) | | 29090.92(20093.29-39371.97) | 410.58(287.39-546.22) | -28.65(-46.46--1.29) | -1.31(-1.42--1.19) |
| Federated States of Micronesia | 44.37(34.22-57.35) | 86.36(67.35-110.45) | | 59.67(43.91-80.31) | 72.06(54.08-95.92) | -16.56(-42.29-24.77) | -0.69(-0.74--0.65) |
| Federative Republic of Brazil | 175844.88(170377.58-180734.59) | 183.52(176.81-188.74) | | 342230.08(325426.76-356454.79) | 132.78(126.26-138.35) | -27.65(-31.28--24.16) | -1.06(-1.16--0.95) |
| French Republic | 159850.71(150363.84-169863.88) | 216.10(203.46-229.80) | | 107528.81(97129.17-117303.84) | 88.64(80.73-95.95) | -58.98(-63.09--54.70) | -2.92(-3.06--2.77) |
| Gabonese Republic | 1999.17(1485.68-2542.37) | 337.16(251.22-426.97) | | 3209.22(2280.75-4263.03) | 277.42(199.59-360.92) | -17.72(-40.35-13.50) | -0.80(-0.93--0.68) |
| Georgia | 3628.27(3162.34-4155.75) | 56.36(49.21-64.47) | | 1576.35(1338.84-1849.43) | 27.43(23.33-32.09) | -51.33(-60.55--41.02) | -1.54(-2.05--1.02) |
| Grand Duchy of Luxembourg | 642.44(605.23-679.35) | 122.96(115.87-130.07) | | 800.00(724.53-884.64) | 78.00(70.60-86.25) | -36.57(-43.31--29.00) | -1.50(-1.68--1.33) |
| Greenland | 169.60(143.99-197.84) | 437.48(374.32-506.61) | | 210.66(167.27-258.34) | 272.77(217.03-334.96) | -37.65(-52.98--20.25) | -1.42(-1.48--1.37) |
| Grenada | 115.19(103.10-128.18) | 176.76(157.85-197.80) | | 150.22(126.38-176.51) | 122.78(103.70-143.29) | -30.54(-43.13--16.36) | -0.62(-1.06--0.17) |
| Guam | 46.75(40.29-53.75) | 57.08(49.27-65.26) | | 106.90(89.48-125.89) | 51.70(43.22-60.85) | -9.42(-27.38-11.76) | 0.54(0.16-0.93) |
| Hashemite Kingdom of Jordan | 421.37(327.73-527.23) | 28.83(22.64-35.83) | | 1637.14(1243.83-2111.97) | 20.17(15.53-25.91) | -30.04(-50.39--0.22) | -1.45(-1.70--1.19) |
| Hellenic Republic | 7438.41(7023.27-7844.13) | 49.29(46.57-51.86) | | 8175.68(7590.56-8674.41) | 40.62(38.21-43.13) | -17.59(-23.68--11.35) | -0.84(-1.04--0.63) |
| Hungary | 17829.39(16519.02-19165.54) | 128.98(119.34-138.43) | | 14800.85(13070.83-16774.22) | 87.03(76.55-98.84) | -32.53(-40.65--22.01) | -1.84(-2.19--1.49) |
| Independent State of Papua New Guinea | 1036.83(709.34-1537.95) | 51.52(35.86-74.89) | | 2321.54(1613.07-3322.04) | 41.07(28.72-58.69) | -20.29(-51.70-20.10) | -0.84(-0.90--0.78) |
| Independent State of Samoa | 29.18(22.69-36.64) | 32.79(25.57-40.76) | | 44.07(33.57-56.32) | 28.91(22.24-36.88) | -11.82(-34.51-24.69) | -0.41(-0.49--0.32) |
| Ireland | 7141.60(6727.62-7609.93) | 179.70(169.37-191.24) | | 9183.94(8233.83-10069.02) | 120.15(108.17-131.85) | -33.14(-40.06--25.74) | -1.11(-1.21--1.01) |
| Islamic Republic of Afghanistan | 19373.44(8993.86-28546.45) | 261.91(124.81-382.36) | | 22621.89(10196.87-34777.66) | 197.42(92.41-295.41) | -24.62(-45.99-11.66) | -1.12(-1.29--0.95) |
| Islamic Republic of Iran | 34925.42(29950.52-39099.12) | 126.69(108.34-141.56) | | 68678.43(62630.37-74957.47) | 86.71(78.90-94.51) | -31.56(-40.89--19.33) | -1.24(-1.34--1.14) |
| Islamic Republic of Mauritania | 982.69(740.95-1255.65) | 94.91(71.89-121.28) | | 2997.45(2119.39-4202.57) | 132.93(93.64-184.97) | 40.06(-2.03-93.85) | 1.50(1.16-1.84) |
| Islamic Republic of Pakistan | 99797.04(83926.17-115914.78) | 167.82(141.76-193.56) | | 228390.68(182178.13-289878.53) | 168.00(133.54-212.35) | 0.11(-21.80-32.28) | -0.37(-0.62--0.12) |
| Jamaica | 1264.82(1177.89-1360.23) | 72.98(67.92-78.47) | | 2321.06(1714.62-3036.21) | 75.25(55.67-98.34) | 3.11(-23.68-35.77) | 0.11(-0.37-0.60) |
| Japan | 204566.68(197740.15-208796.50) | 118.07(113.93-120.60) | | 267046.36(244223.37-279927.74) | 84.34(78.99-87.57) | -28.57(-31.07--26.57) | -1.21(-1.41--1.02) |
| Kingdom of Bahrain | 163.01(137.17-189.89) | 90.46(76.76-105.13) | | 418.30(325.45-548.67) | 45.61(36.18-57.24) | -49.58(-61.60--33.59) | -3.13(-3.50--2.75) |
| Kingdom of Belgium | 14396.29(13514.67-15319.90) | 101.20(95.31-107.16) | | 20428.02(18838.58-22068.06) | 97.70(90.31-105.10) | -3.47(-12.09-5.06) | -0.19(-0.46-0.07) |
| Kingdom of Bhutan | 387.20(276.54-527.80) | 139.78(100.36-189.61) | | 576.92(410.75-807.53) | 91.87(65.63-128.50) | -34.27(-56.24-2.51) | -1.44(-1.66--1.23) |
| Kingdom of Cambodia | 5978.84(4578.98-7536.79) | 118.87(92.05-149.47) | | 10455.08(7574.49-13840.17) | 77.76(56.89-102.65) | -34.58(-54.78--7.70) | -1.58(-1.68--1.47) |
| Kingdom of Denmark | 8967.62(8484.95-9469.34) | 121.96(115.50-128.49) | | 11680.10(10641.30-12530.26) | 106.78(97.80-114.86) | -12.45(-20.43--4.56) | -0.58(-0.73--0.42) |
| Kingdom of Eswatini | 1464.41(1083.52-1886.79) | 457.53(339.53-581.34) | | 3104.83(2112.13-4386.97) | 478.85(332.56-665.21) | 4.66(-26.76-53.54) | 0.39(-0.33-1.12) |
| Kingdom of Lesotho | 2446.77(1874.08-3151.41) | 277.58(213.01-357.19) | | 5307.80(3839.22-6987.96) | 450.01(327.62-588.49) | 62.12(15.28-134.14) | 2.14(1.63-2.66) |
| Kingdom of Morocco | 3173.46(2481.97-3919.81) | 21.48(16.75-26.50) | | 7681.12(5482.63-9926.48) | 21.36(15.36-27.30) | -0.54(-28.61-32.77) | 0.07(-0.04-0.18) |
| Kingdom of Norway | 3767.38(3626.56-3896.93) | 61.12(59.14-63.18) | | 6093.77(5701.23-6391.33) | 64.09(60.46-67.04) | 4.86(-0.21-10.30) | 0.12(-0.04-0.27) |
| Kingdom of Saudi Arabia | 3420.66(2430.91-4663.44) | 54.80(39.31-73.76) | | 9773.16(7331.44-13484.03) | 41.63(32.40-54.55) | -24.03(-48.99-10.82) | -1.21(-1.34--1.08) |
| Kingdom of Spain | 55592.27(52507.72-58824.02) | 109.78(103.33-116.07) | | 51240.70(46889.35-55433.46) | 60.24(55.22-65.15) | -45.13(-49.80--39.56) | -2.04(-2.13--1.95) |
| Kingdom of Sweden | 8487.76(8009.87-8904.13) | 60.57(57.53-63.51) | | 11286.06(9814.13-12626.23) | 56.48(49.49-63.53) | -6.76(-18.97-5.88) | -0.09(-0.26-0.08) |
| Kingdom of Thailand | 51500.00(40451.49-69513.66) | 131.03(102.87-176.00) | | 134350.33(99350.61-177193.04) | 124.84(92.39-164.75) | -4.72(-37.21-46.66) | -0.47(-0.60--0.33) |
| Kingdom of the Netherlands | 21875.85(20653.25-23064.81) | 114.56(108.27-120.99) | | 46289.55(42366.81-49717.61) | 137.08(126.38-147.01) | 19.65(10.10-29.36) | 0.64(0.33-0.94) |
| Kingdom of Tonga | 29.45(23.66-36.57) | 51.39(41.12-63.54) | | 40.46(30.48-53.26) | 49.66(37.64-64.90) | -3.36(-31.25-32.82) | -0.22(-0.41--0.03) |
| Kyrgyz Republic | 7116.56(6112.28-8171.01) | 229.89(198.38-264.05) | | 3693.56(2886.23-4569.70) | 72.51(56.99-88.94) | -68.46(-75.23--59.37) | -3.71(-3.82--3.61) |
| Lao People's Democratic Republic | 2847.80(2025.95-3854.33) | 124.04(88.60-167.07) | | 3386.70(2451.13-4752.21) | 66.11(48.58-92.57) | -46.70(-65.84--17.15) | -2.21(-2.30--2.12) |
| Lebanese Republic | 748.17(578.04-957.18) | 32.88(25.63-41.80) | | 1234.26(982.28-1517.48) | 20.85(16.58-25.70) | -36.57(-53.47--10.15) | -1.13(-1.33--0.92) |
| Malaysia | 6594.92(5523.87-7661.44) | 68.38(57.28-79.31) | | 19234.32(16300.90-22633.46) | 64.90(54.96-76.09) | -5.10(-24.76-18.82) | -0.30(-0.47--0.13) |
| Mongolia | 6456.53(5243.05-7954.80) | 600.56(490.34-739.28) | | 9354.08(7332.62-11600.70) | 397.98(317.66-481.22) | -33.73(-50.50--12.67) | -1.66(-1.82--1.51) |
| Montenegro | 363.32(298.95-444.28) | 55.12(45.34-67.48) | | 557.71(427.51-715.82) | 57.88(44.64-74.13) | 5.02(-23.51-49.30) | 0.34(0.22-0.45) |
| New Zealand | 4123.82(3879.08-4344.74) | 105.92(99.66-111.83) | | 5770.42(5348.12-6180.82) | 69.67(64.87-74.50) | -34.22(-38.92--28.44) | -1.48(-1.65--1.30) |
| North Macedonia | 765.34(659.09-904.34) | 38.65(33.62-45.33) | | 1184.97(893.21-1521.29) | 35.28(26.69-45.06) | -8.72(-32.55-24.34) | -0.38(-0.66--0.10) |
| Northern Mariana Islands | 7.27(5.59-9.75) | 34.36(27.65-44.63) | | 34.75(28.64-40.77) | 62.76(52.12-73.90) | 82.68(34.58-139.49) | 3.07(2.46-3.67) |
| Palestine | 291.99(211.90-386.25) | 33.29(24.42-43.63) | | 546.02(438.61-674.74) | 20.82(16.83-25.57) | -37.47(-55.72--11.20) | -1.73(-1.92--1.55) |
| People's Democratic Republic of Algeria | 2243.97(1832.35-2700.77) | 18.22(14.88-21.72) | | 5982.93(4532.69-7551.51) | 16.36(12.54-20.52) | -10.18(-35.24-18.20) | -0.32(-0.36--0.27) |
| People's Republic of Bangladesh | 74614.36(53817.08-100295.70) | 146.09(104.47-197.09) | | 124792.87(87649.82-175595.81) | 86.66(61.04-121.66) | -40.68(-58.66--15.65) | -1.61(-1.72--1.49) |
| People's Republic of China | 5852132.32(4841614.21-6818927.28) | 653.31(543.18-758.88) | | 6898666.18(5471181.02-8553365.98) | 317.18(252.46-392.42) | -51.45(-63.81--36.54) | -2.67(-2.91--2.43) |
| Plurinational State of Bolivia | 2337.82(1779.87-3018.51) | 70.78(53.88-90.88) | | 4871.75(3510.10-6693.94) | 52.85(38.37-71.77) | -25.33(-47.40-9.08) | -1.00(-1.10--0.90) |
| Portuguese Republic | 15946.02(15023.10-16935.08) | 118.92(112.03-126.43) | | 17156.79(15727.65-18607.44) | 85.41(78.58-92.66) | -28.18(-35.16--20.40) | -0.96(-1.16--0.76) |
| Principality of Andorra | 28.65(19.53-41.49) | 48.79(33.29-70.61) | | 43.61(27.84-63.54) | 28.60(18.34-41.58) | -41.39(-67.40--2.81) | -1.41(-1.67--1.16) |
| Principality of Monaco | 71.19(54.21-88.79) | 116.38(89.73-145.11) | | 107.65(84.84-132.72) | 127.03(98.32-157.38) | 9.15(-20.20-51.31) | 0.43(0.19-0.67) |
| Puerto Rico | 5462.20(5153.46-5820.10) | 151.96(143.54-161.67) | | 3635.25(2966.89-4285.00) | 59.50(48.62-70.75) | -60.85(-68.21--53.09) | -2.88(-3.11--2.66) |
| Republic of Albania | 1057.86(883.98-1270.76) | 48.87(40.84-58.74) | | 1492.30(1088.32-1971.03) | 35.34(25.92-46.37) | -27.68(-47.99-1.82) | -0.78(-1.02--0.54) |
| Republic of Angola | 14593.35(9760.48-19971.11) | 326.22(220.63-440.80) | | 28517.75(20271.30-38319.14) | 212.85(153.26-279.88) | -34.75(-54.32--2.92) | -1.59(-1.71--1.47) |
| Republic of Armenia | 2064.57(1902.34-2222.32) | 72.45(66.54-78.16) | | 1109.34(972.18-1274.09) | 24.94(21.88-28.62) | -65.57(-70.94--59.20) | -3.30(-3.68--2.92) |
| Republic of Austria | 7182.86(6752.92-7602.04) | 67.10(63.07-71.20) | | 9094.85(8313.55-9795.42) | 55.39(50.91-59.59) | -17.45(-24.47--9.42) | -0.43(-0.57--0.29) |
| Republic of Azerbaijan | 12063.30(10484.06-13714.53) | 227.43(197.38-260.20) | | 14738.75(10874.83-18781.58) | 135.65(99.95-174.28) | -40.35(-56.86--22.82) | -1.61(-1.80--1.42) |
| Republic of Belarus | 10667.00(9863.48-11552.71) | 81.19(75.21-88.02) | | 12754.16(10231.73-15907.62) | 81.52(65.90-101.55) | 0.41(-20.78-27.47) | -0.46(-0.65--0.28) |
| Republic of Benin | 1729.77(1412.77-2105.62) | 84.44(69.36-102.55) | | 7435.66(5516.97-9901.92) | 132.87(99.51-174.31) | 57.35(12.86-117.36) | 2.04(1.80-2.28) |
| Republic of Botswana | 2011.39(1435.58-2755.22) | 330.63(236.54-447.50) | | 3659.86(2690.40-4834.25) | 226.60(168.23-296.06) | -31.46(-53.48--0.33) | -1.53(-1.85--1.20) |
| Republic of Bulgaria | 8662.17(8065.67-9318.29) | 70.23(65.64-75.44) | | 7724.63(6591.17-8877.38) | 62.88(53.55-72.67) | -10.47(-24.65-5.86) | -0.64(-0.91--0.36) |
| Republic of Burundi | 13276.92(9416.96-16823.81) | 536.74(383.17-675.73) | | 15545.95(11158.77-20454.09) | 285.03(205.13-369.84) | -46.90(-62.18--24.09) | -2.68(-2.95--2.41) |
| Republic of Cabo Verde | 502.57(422.73-592.91) | 226.31(191.19-266.42) | | 1856.43(1451.22-2296.43) | 398.29(311.28-491.88) | 76.00(34.21-128.38) | 1.36(1.02-1.71) |
| Republic of Cameroon | 4570.44(3429.31-5809.65) | 95.07(72.19-119.96) | | 23887.02(16061.75-33363.42) | 169.45(115.01-235.52) | 78.24(25.04-153.07) | 2.36(2.15-2.58) |
| Republic of Chad | 1968.95(1587.05-2398.09) | 67.98(54.73-82.53) | | 9243.89(6676.33-12289.40) | 146.17(107.52-192.43) | 115.03(57.04-200.88) | 3.04(2.82-3.26) |
| Republic of Chile | 17478.36(16576.98-18351.10) | 173.88(164.81-182.72) | | 16434.60(15098.90-17666.71) | 63.99(58.76-68.78) | -63.20(-66.07--59.88) | -3.47(-3.64--3.30) |
| Republic of Colombia | 18455.53(17617.29-19345.79) | 101.21(96.24-106.16) | | 22601.34(18700.80-27249.95) | 40.93(33.94-49.21) | -59.56(-66.35--51.19) | -3.35(-3.51--3.18) |
| Republic of Costa Rica | 1058.70(979.36-1132.50) | 59.94(55.43-64.07) | | 2032.89(1769.38-2291.62) | 36.83(32.09-41.46) | -38.55(-47.00--30.42) | -1.87(-2.02--1.71) |
| Republic of Croatia | 6477.41(5993.17-6971.05) | 101.29(94.13-108.65) | | 5248.63(4582.17-5938.81) | 66.80(58.14-75.87) | -34.05(-44.36--24.18) | -1.40(-1.60--1.20) |
| Republic of Cuba | 9413.83(8929.21-9945.03) | 92.20(87.44-97.36) | | 24758.46(20903.82-28811.66) | 128.61(108.42-149.64) | 39.49(18.47-63.95) | 1.51(1.34-1.68) |
| Republic of Cyprus | 242.53(195.85-297.97) | 31.56(25.72-38.38) | | 598.35(465.45-765.15) | 29.77(23.23-37.84) | -5.68(-30.87-31.07) | 0.50(0.26-0.74) |
| Republic of Cote d' Ivoire | 1559.74(1224.14-1913.91) | 34.79(27.84-42.08) | | 4369.97(3160.22-6061.09) | 34.70(25.72-46.77) | -0.27(-26.63-39.81) | -0.06(-0.23-0.12) |
| Republic of Djibouti | 641.27(440.97-909.42) | 401.33(283.99-552.42) | | 2363.88(1529.78-3492.74) | 320.56(213.23-464.52) | -20.12(-45.26-18.43) | -0.94(-1.06--0.82) |
| Republic of Ecuador | 2710.64(2549.63-2855.85) | 50.08(46.98-52.82) | | 4496.25(3509.50-5632.29) | 27.39(21.40-34.22) | -45.30(-57.26--30.80) | -1.61(-1.89--1.32) |
| Republic of El Salvador | 1457.60(1306.36-1620.76) | 47.60(42.67-52.96) | | 2600.07(2072.19-3293.68) | 42.50(33.81-53.92) | -10.73(-31.69-14.72) | -0.64(-0.85--0.43) |
| Republic of Equatorial Guinea | 804.82(552.76-1062.25) | 371.13(257.16-487.55) | | 1314.32(869.95-1938.13) | 229.69(155.89-333.53) | -38.11(-60.11--6.49) | -1.76(-2.16--1.36) |
| Republic of Estonia | 1876.58(1722.84-2024.22) | 91.85(84.92-98.88) | | 1554.44(1325.26-1813.72) | 65.92(56.24-77.00) | -28.23(-39.69--13.73) | -1.42(-1.65--1.20) |
| Republic of Fiji | 235.07(185.89-298.58) | 59.35(47.16-74.89) | | 532.04(375.64-703.56) | 66.24(46.72-86.77) | 11.62(-22.61-57.19) | 0.59(0.40-0.79) |
| Republic of Finland | 5457.34(5198.03-5711.15) | 79.66(75.91-83.41) | | 7367.68(6792.81-7881.07) | 66.18(61.74-70.66) | -16.92(-23.16--10.60) | -0.53(-0.61--0.45) |
| Republic of Ghana | 4454.37(3369.02-5775.69) | 65.06(49.68-83.15) | | 16371.39(11320.74-21334.37) | 90.52(61.71-117.85) | 39.13(-4.29-91.60) | 1.58(1.39-1.77) |
| Republic of Guatemala | 2283.17(2193.29-2375.44) | 63.30(60.73-65.80) | | 4582.91(3881.08-5387.48) | 40.65(34.54-47.76) | -35.79(-45.80--24.63) | -1.61(-1.88--1.34) |
| Republic of Guinea | 1110.38(871.48-1373.67) | 32.31(25.39-39.97) | | 2161.51(1522.05-2953.90) | 36.04(25.48-49.30) | 11.57(-29.01-67.13) | 0.59(0.43-0.75) |
| Republic of Guinea-Bissau | 571.68(419.12-726.03) | 131.96(97.58-166.91) | | 1677.72(1234.66-2154.40) | 201.13(149.66-253.61) | 52.42(9.96-117.29) | 2.02(1.77-2.28) |
| Republic of Guyana | 230.63(205.09-260.03) | 57.26(50.98-64.15) | | 403.38(302.99-522.70) | 57.17(43.20-73.71) | -0.15(-27.13-32.35) | 0.38(0.20-0.56) |
| Republic of Haiti | 5031.99(3588.57-6622.50) | 143.74(102.63-187.88) | | 8474.83(5893.76-12126.35) | 106.31(74.13-150.33) | -26.04(-48.38-2.39) | -0.77(-0.88--0.66) |
| Republic of Honduras | 565.33(464.38-675.00) | 26.18(21.55-31.23) | | 2268.03(1766.78-2937.98) | 35.29(27.38-45.12) | 34.79(1.34-79.39) | 1.27(1.11-1.44) |
| Republic of Iceland | 294.54(273.66-315.35) | 107.79(100.13-115.44) | | 485.01(434.83-539.31) | 89.55(80.22-99.09) | -16.93(-25.94--6.39) | -0.71(-0.86--0.56) |
| Republic of India | 525762.49(458716.70-653743.51) | 99.41(86.45-124.39) | | 1056497.11(927426.25-1250177.01) | 83.40(73.11-99.11) | -16.10(-27.22--0.38) | -0.82(-0.94--0.70) |
| Republic of Indonesia | 56368.14(41756.23-67913.82) | 50.90(37.89-61.80) | | 108596.77(83024.20-136774.42) | 41.44(31.71-52.06) | -18.60(-37.30-4.40) | -0.67(-0.72--0.61) |
| Republic of Iraq | 2693.76(2087.51-3415.44) | 32.28(25.03-40.91) | | 7509.78(5367.94-9849.36) | 29.09(20.94-37.44) | -9.88(-40.26-31.40) | -0.65(-0.78--0.53) |
| Republic of Italy | 69321.13(66589.17-71519.27) | 82.26(79.19-84.94) | | 47549.58(44307.81-50005.10) | 37.13(35.08-38.91) | -54.86(-56.70--52.89) | -2.58(-2.67--2.49) |
| Republic of Kazakhstan | 65421.35(60928.72-69983.71) | 500.50(465.90-537.45) | | 22414.58(19347.37-25881.50) | 119.34(103.25-137.70) | -76.16(-79.60--72.17) | -4.70(-4.93--4.46) |
| Republic of Kenya | 21445.99(15566.27-30697.00) | 242.78(175.43-348.16) | | 76416.53(55651.23-107056.91) | 306.12(222.37-429.01) | 26.09(-2.66-65.50) | 1.12(0.88-1.37) |
| Republic of Kiribati | 68.87(53.87-86.24) | 168.80(133.19-209.92) | | 123.05(86.51-163.28) | 150.72(108.34-197.39) | -10.71(-37.87-24.74) | -0.49(-0.59--0.38) |
| Republic of Korea | 48742.16(39278.99-59170.55) | 148.53(120.20-180.03) | | 48069.63(38607.55-59204.59) | 50.15(40.36-61.89) | -66.24(-75.04--54.07) | -4.00(-4.22--3.77) |
| Republic of Latvia | 3068.55(2826.80-3305.25) | 86.01(79.21-92.72) | | 2669.46(2277.02-3077.98) | 78.33(66.70-90.89) | -8.93(-23.31-6.57) | -0.42(-0.62--0.22) |
| Republic of Liberia | 980.99(761.27-1220.67) | 82.31(64.48-102.16) | | 3618.30(2463.06-5287.40) | 150.61(103.61-214.96) | 82.96(26.95-162.07) | 2.57(2.22-2.93) |
| Republic of Lithuania | 3995.86(3734.63-4266.81) | 88.39(82.73-94.34) | | 4859.59(4179.28-5576.84) | 97.28(83.82-111.39) | 10.05(-6.46-26.65) | 0.29(0.06-0.52) |
| Republic of Madagascar | 22207.25(16247.06-27217.65) | 401.58(294.99-489.61) | | 38197.85(26089.48-52767.12) | 291.34(199.80-398.82) | -27.45(-48.32--3.16) | -1.15(-1.25--1.06) |
| Republic of Malawi | 24766.88(20298.30-29765.01) | 584.02(479.77-700.61) | | 59610.56(47284.02-76806.17) | 715.28(572.77-904.41) | 22.47(-6.78-59.28) | 0.61(0.20-1.03) |
| Republic of Maldives | 87.65(60.80-113.58) | 84.30(59.82-108.35) | | 105.52(80.59-134.24) | 27.83(21.73-34.75) | -66.99(-75.49--50.80) | -4.07(-4.33--3.82) |
| Republic of Mali | 2821.63(2354.96-3356.08) | 65.48(55.03-77.26) | | 6241.40(4724.97-8157.94) | 65.07(49.53-84.49) | -0.62(-30.53-34.66) | 0.26(0.12-0.40) |
| Republic of Malta | 295.06(272.56-320.43) | 68.93(63.81-74.69) | | 441.47(393.64-489.10) | 52.06(46.47-57.45) | -24.47(-34.30--14.50) | -0.85(-0.98--0.72) |
| Republic of Mauritius | 741.33(703.30-780.02) | 95.65(90.86-100.54) | | 1737.64(1594.27-1838.45) | 92.68(85.24-97.68) | -3.10(-12.21-4.63) | -0.74(-1.17--0.30) |
| Republic of Moldova | 3144.27(2944.31-3327.70) | 68.16(64.00-71.99) | | 2970.43(2651.91-3329.06) | 50.04(44.81-55.89) | -26.59(-35.03--15.90) | -0.87(-1.32--0.41) |
| Republic of Mozambique | 11761.13(9328.61-14541.08) | 192.17(153.19-236.15) | | 27406.21(20218.86-35071.42) | 232.62(175.47-293.13) | 21.05(-15.58-61.82) | 1.15(0.94-1.36) |
| Republic of Namibia | 462.15(371.22-567.71) | 65.41(53.15-80.22) | | 1014.99(744.24-1407.65) | 65.51(49.50-88.59) | 0.15(-27.99-43.35) | -0.23(-0.60-0.14) |
| Republic of Nauru | 5.75(4.17-7.72) | 109.70(80.34-143.94) | | 5.92(4.19-7.81) | 89.22(64.21-115.44) | -18.67(-42.28-12.39) | -0.94(-1.09--0.79) |
| Republic of Nicaragua | 412.95(353.46-467.07) | 25.71(22.02-29.05) | | 1030.76(833.44-1283.01) | 20.45(16.57-25.24) | -20.48(-37.48-2.07) | -0.68(-0.91--0.46) |
| Republic of Niue | 1.28(1.01-1.58) | 60.02(46.91-74.57) | | 1.32(1.00-1.66) | 60.59(45.97-76.26) | 0.96(-25.35-36.60) | -0.21(-0.31--0.11) |
| Republic of Palau | 7.94(6.25-10.27) | 79.08(62.52-100.58) | | 16.60(12.96-21.25) | 71.13(56.17-89.51) | -10.04(-33.95-22.05) | -0.32(-0.37--0.27) |
| Republic of Panama | 670.55(629.98-712.27) | 44.50(41.67-47.50) | | 1254.53(969.50-1519.34) | 28.40(21.95-34.44) | -36.18(-50.45--22.65) | -1.29(-1.45--1.13) |
| Republic of Paraguay | 1878.75(1570.81-2274.62) | 82.18(68.75-99.24) | | 6314.15(4626.10-8426.40) | 104.67(77.00-139.44) | 27.36(-11.24-76.53) | 0.72(0.56-0.87) |
| Republic of Peru | 5189.26(4261.86-6331.52) | 42.46(34.83-51.78) | | 9799.83(7156.84-13139.27) | 29.10(21.30-38.92) | -31.48(-52.08--2.80) | -1.56(-1.87--1.25) |
| Republic of Poland | 42848.84(41396.16-44177.94) | 99.09(95.77-102.18) | | 53221.09(48089.91-58062.58) | 80.97(73.46-88.34) | -18.28(-26.38--10.72) | -0.92(-1.10--0.75) |
| Republic of Rwanda | 18736.08(12719.64-23689.68) | 596.51(407.81-751.87) | | 20353.30(14498.36-27401.63) | 295.32(214.93-395.44) | -50.49(-66.23--26.91) | -3.38(-3.83--2.92) |
| Republic of San Marino | 12.78(9.97-16.01) | 38.56(30.09-48.47) | | 13.32(8.26-19.82) | 20.32(12.09-30.60) | -47.31(-69.39--13.56) | -1.12(-1.47--0.77) |
| Republic of Senegal | 2780.46(2214.43-3397.67) | 81.55(65.56-99.27) | | 10960.42(8177.90-14607.84) | 132.95(99.51-173.99) | 63.02(21.73-121.56) | 2.22(1.86-2.58) |
| Republic of Serbia | 7668.89(5682.09-10486.03) | 64.34(48.13-87.42) | | 8522.05(6121.88-11826.55) | 56.21(40.26-78.21) | -12.64(-41.97-33.40) | -0.57(-0.74--0.40) |
| Republic of Seychelles | 85.15(70.91-100.97) | 153.16(127.60-181.98) | | 157.24(126.64-193.74) | 124.35(100.17-152.45) | -18.81(-37.14-7.51) | -0.56(-0.73--0.40) |
| Republic of Sierra Leone | 1564.38(1188.15-1976.16) | 74.50(57.13-93.79) | | 5246.46(3778.23-7196.18) | 128.69(92.70-174.10) | 72.74(25.44-135.70) | 2.55(2.25-2.86) |
| Republic of Singapore | 2339.70(2205.03-2493.65) | 102.43(96.45-109.17) | | 3305.38(2995.74-3574.10) | 37.68(34.22-40.64) | -63.21(-66.71--59.57) | -3.26(-3.60--2.93) |
| Republic of Slovenia | 2325.97(2170.95-2489.96) | 93.81(87.64-100.27) | | 2179.97(1849.05-2485.60) | 55.03(46.46-62.73) | -41.34(-50.66--32.90) | -2.08(-2.27--1.90) |
| Republic of South Africa | 73765.93(65650.77-86961.66) | 322.87(286.32-380.88) | | 138651.21(125929.52-155949.66) | 279.79(254.98-313.69) | -13.34(-28.58-0.13) | -1.00(-1.50--0.48) |
| Republic of South Sudan | 12962.99(9507.79-17892.41) | 481.42(353.65-661.88) | | 18316.47(13027.96-24573.24) | 411.79(298.26-551.71) | -14.46(-37.63-22.80) | -0.79(-1.00--0.58) |
| Republic of Sudan | 12728.49(7721.13-17328.06) | 128.60(79.43-173.70) | | 23043.26(13703.86-33219.64) | 108.35(66.93-154.98) | -15.75(-42.96-23.86) | -0.60(-0.65--0.55) |
| Republic of Suriname | 126.64(107.33-146.64) | 47.04(40.21-54.30) | | 294.54(218.53-381.25) | 44.12(32.81-56.74) | -6.20(-34.46-25.07) | 0.04(-0.20-0.28) |
| Republic of Tajikistan | 8645.63(7050.77-10352.77) | 304.18(249.15-365.23) | | 9340.97(6786.29-12297.27) | 153.52(113.81-197.77) | -49.53(-64.45--29.01) | -2.30(-2.50--2.09) |
| Republic of the Congo | 5151.94(3778.21-6813.52) | 439.79(325.80-577.56) | | 8758.13(6049.82-12181.19) | 281.50(198.91-380.86) | -35.99(-54.24--11.54) | -1.78(-1.96--1.60) |
| Republic of the Gambia | 151.41(114.17-194.47) | 40.26(30.78-50.99) | | 538.75(406.58-696.92) | 51.54(38.88-65.80) | 28.02(-9.96-74.54) | 0.74(0.60-0.89) |
| Republic of the Marshall Islands | 13.14(8.89-17.47) | 74.80(51.59-97.96) | | 26.84(19.08-36.46) | 66.78(49.64-89.36) | -10.73(-37.87-28.58) | -0.30(-0.38--0.22) |
| Republic of the Niger | 2284.55(1692.89-2911.14) | 75.41(56.69-95.12) | | 9468.88(6440.51-12924.17) | 107.27(73.68-143.73) | 42.26(1.06-103.72) | 1.79(1.52-2.05) |
| Republic of the Philippines | 13013.14(11510.08-15085.51) | 38.02(33.47-44.34) | | 33042.87(26837.32-39534.25) | 36.37(29.66-43.52) | -4.34(-23.74-18.07) | -0.12(-0.23--0.02) |
| Republic of the Union of Myanmar | 28670.69(20982.62-37268.28) | 111.85(83.35-144.67) | | 30961.37(23496.57-42549.73) | 59.51(45.59-81.41) | -46.79(-64.23--19.56) | -2.39(-2.52--2.26) |
| Republic of Trinidad and Tobago | 522.29(493.18-552.51) | 61.50(58.06-65.12) | | 1002.81(761.19-1291.19) | 51.79(39.32-66.65) | -15.80(-36.52-9.31) | -0.57(-0.81--0.33) |
| Republic of Tunisia | 856.17(679.33-1058.92) | 16.52(13.18-20.36) | | 2166.40(1459.47-3059.34) | 15.83(10.75-22.28) | -4.19(-37.93-40.19) | -0.26(-0.31--0.21) |
| Republic of Turkey | 24054.09(19012.78-29224.63) | 64.08(51.68-77.56) | | 37098.07(27841.76-46777.31) | 38.74(29.16-48.69) | -39.54(-56.00--19.05) | -1.80(-2.07--1.53) |
| Republic of Uganda | 26809.90(21023.81-33339.12) | 391.45(309.40-485.10) | | 64889.67(48346.31-85796.24) | 395.91(302.09-521.13) | 1.14(-31.83-44.72) | -0.44(-0.70--0.17) |
| Republic of Uzbekistan | 46168.33(41815.84-50573.77) | 388.45(351.91-426.79) | | 28051.95(22930.55-33988.69) | 98.60(81.21-118.15) | -74.62(-79.31--69.54) | -4.81(-5.42--4.18) |
| Republic of Vanuatu | 46.10(33.06-63.59) | 66.35(48.88-90.05) | | 114.10(87.16-150.28) | 59.09(45.86-76.54) | -10.95(-41.55-26.74) | -0.50(-0.56--0.43) |
| Republic of Yemen | 7210.93(4173.85-10344.26) | 135.25(78.31-193.01) | | 15081.71(7659.00-22075.08) | 98.89(51.16-142.83) | -26.88(-51.93-13.49) | -1.29(-1.39--1.18) |
| Republic of Zambia | 16956.93(12808.37-20880.39) | 535.95(408.88-662.05) | | 34918.92(22964.52-55564.82) | 436.30(290.09-677.10) | -18.59(-44.68-31.71) | -1.16(-1.40--0.92) |
| Republic of Zimbabwe | 14375.86(11465.20-17331.85) | 333.04(265.98-399.56) | | 34110.09(25271.66-43725.38) | 435.15(329.44-549.19) | 30.66(-6.77-75.74) | 1.03(0.53-1.54) |
| Romania | 12329.19(11593.28-13205.02) | 43.51(40.92-46.47) | | 23274.36(20437.11-26424.17) | 72.12(63.27-82.08) | 65.76(43.09-92.23) | 1.18(0.87-1.50) |
| Russian Federation | 252619.83(247639.26-257196.41) | 136.17(133.43-138.67) | | 201900.78(182635.80-219875.16) | 86.83(78.57-94.59) | -36.23(-42.43--30.73) | -1.68(-1.91--1.46) |
| Saint Kitts and Nevis | 40.93(38.28-43.52) | 118.42(110.68-125.95) | | 74.47(60.77-88.66) | 97.62(80.24-115.04) | -17.57(-33.44--1.19) | -0.19(-0.49-0.11) |
| Saint Lucia | 115.79(108.79-123.53) | 134.25(126.24-143.14) | | 267.07(214.85-323.26) | 108.84(87.96-131.40) | -18.92(-34.20--1.26) | -0.68(-0.95--0.41) |
| Saint Vincent and the Grenadines | 42.97(39.33-46.60) | 61.23(56.16-66.44) | | 96.04(84.31-110.87) | 66.24(58.15-76.24) | 8.18(-7.84-28.41) | 0.41(0.13-0.68) |
| Slovak Republic | 7007.20(5332.78-9182.98) | 120.37(92.09-157.57) | | 7863.60(5901.37-10776.87) | 87.88(65.22-121.55) | -26.99(-52.81-10.52) | -1.01(-1.16--0.87) |
| Socialist Republic of Viet Nam | 22971.55(16926.96-30619.03) | 55.34(40.99-73.59) | | 66139.74(47736.42-86249.46) | 60.59(44.43-77.89) | 9.49(-24.78-67.10) | 0.57(0.46-0.67) |
| Solomon Islands | 113.77(71.96-157.02) | 74.67(49.92-101.06) | | 261.27(174.05-371.28) | 65.57(44.23-91.53) | -12.19(-41.73-40.65) | -0.47(-0.53--0.40) |
| State of Eritrea | 8111.01(5216.44-10824.65) | 578.22(380.21-764.92) | | 12510.15(8538.71-18170.90) | 391.03(270.15-566.88) | -32.37(-50.73--8.82) | -1.41(-1.51--1.32) |
| State of Israel | 2179.33(2044.07-2327.63) | 46.08(43.36-49.15) | | 3806.05(3476.17-4093.71) | 32.43(29.73-34.82) | -29.62(-36.04--22.98) | -1.31(-1.50--1.12) |
| State of Kuwait | 302.97(279.06-327.51) | 44.60(40.96-48.49) | | 633.93(510.05-779.93) | 19.70(15.89-24.31) | -55.82(-64.77--44.76) | -3.08(-3.75--2.40) |
| State of Libya | 734.50(528.98-1001.82) | 36.80(26.81-49.75) | | 2570.99(1755.56-3512.31) | 43.79(30.39-59.01) | 19.00(-23.93-88.58) | 0.83(0.68-0.98) |
| State of Qatar | 135.39(105.54-171.35) | 114.93(90.52-143.11) | | 568.62(400.74-795.48) | 52.60(38.83-70.86) | -54.23(-69.02--33.04) | -2.89(-3.50--2.26) |
| Sultanate of Oman | 361.97(258.30-487.71) | 49.98(36.00-66.88) | | 822.62(599.44-1086.20) | 36.96(27.45-47.50) | -26.04(-50.49-13.07) | -0.72(-0.93--0.51) |
| Swiss Confederation | 10511.30(9901.43-11095.56) | 109.73(103.02-116.11) | | 10709.27(9631.38-11580.88) | 64.24(58.66-69.19) | -41.46(-46.83--36.26) | -1.45(-1.62--1.28) |
| Syrian Arab Republic | 1235.33(953.75-1559.38) | 22.00(17.12-27.57) | | 2872.35(2026.87-3834.14) | 20.90(15.14-27.38) | -4.98(-36.00-43.15) | -0.45(-0.65--0.24) |
| Taiwan (Province of China) | 39779.80(37785.96-42192.32) | 233.26(221.89-247.00) | | 87762.32(78833.88-94875.02) | 219.55(198.14-237.21) | -5.88(-16.40-2.89) | -0.16(-0.55-0.22) |
| Togolese Republic | 1087.73(855.89-1358.35) | 81.08(63.99-101.20) | | 6834.70(4850.45-9350.29) | 157.05(111.16-213.04) | 93.71(38.11-171.40) | 2.74(2.49-2.98) |
| Tokelau | 0.75(0.50-1.00) | 55.38(37.63-73.34) | | 0.68(0.50-0.86) | 45.87(34.06-58.52) | -17.18(-38.67-13.63) | -0.78(-0.84--0.72) |
| Turkmenistan | 15948.27(14862.38-16971.31) | 793.70(742.55-842.70) | | 9667.20(7336.51-12530.02) | 223.55(170.85-288.61) | -71.83(-78.63--63.64) | -4.13(-4.67--3.57) |
| Tuvalu | 5.00(3.87-6.19) | 69.71(54.67-85.32) | | 6.07(4.62-7.69) | 55.90(43.15-70.42) | -19.81(-39.28-10.21) | -0.77(-0.83--0.71) |
| Ukraine | 65566.93(61174.23-70556.04) | 91.80(85.83-98.63) | | 47424.47(31907.79-66342.38) | 66.15(44.54-92.64) | -27.94(-52.62-0.70) | -1.36(-1.60--1.12) |
| Union of the Comoros | 995.81(696.24-1330.66) | 458.77(323.17-611.41) | | 1728.69(1265.15-2336.07) | 329.86(242.64-444.83) | -28.10(-51.88-4.73) | -1.41(-1.59--1.22) |
| United Arab Emirates | 323.03(234.82-441.44) | 60.25(44.32-81.00) | | 1971.84(1468.97-2561.99) | 46.26(35.71-58.38) | -23.22(-43.13-4.70) | 0.32(-0.19-0.84) |
| United Kingdom of Great Britain and Northern Ireland | 143873.60(139692.82-146437.12) | 168.03(163.68-170.78) | | 189121.10(178951.45-195972.03) | 154.57(147.55-159.75) | -8.01(-10.67--5.53) | -0.39(-0.59--0.20) |
| United Mexican States | 23541.85(23054.17-23946.59) | 53.72(52.44-54.73) | | 46851.53(40310.86-53725.80) | 36.05(31.08-41.27) | -32.90(-41.93--23.14) | -1.22(-1.34--1.10) |
| United Republic of Tanzania | 51398.37(38241.15-64755.65) | 438.82(327.37-549.77) | | 83555.55(60796.67-110436.35) | 300.14(220.09-394.93) | -31.60(-50.89--7.07) | -1.50(-1.61--1.40) |
| United States of America | 285637.33(275507.90-291098.13) | 95.83(92.74-97.55) | | 479504.47(457854.35-495409.91) | 86.55(82.92-89.36) | -9.68(-12.24--7.19) | -0.44(-0.57--0.30) |
| United States Virgin Islands | 79.04(63.12-96.25) | 86.76(69.88-105.05) | | 99.51(75.88-130.01) | 61.04(46.48-80.44) | -29.65(-48.88--1.66) | -0.87(-1.02--0.73) |

**DALY, disability-adjusted life-years; EAPC, estimated annual percentage change; CI, confidential interval; UI, uncertainty interval.**

**Supplementary Table 7 Age Patterns by Sex for the Total Number and Age-standardized Incidence Rates of Esophageal Cancer in 2021**

| **Age Group** | **Sex** | **Metric** | **Value** | **Upper** | **Lower** |
| --- | --- | --- | --- | --- | --- |
| 20-24 years | Male | Number | 257.08 | 290.60 | 229.99 |
|  |  | Rate | 0.08 | 0.10 | 0.08 |
| 20-24 years | Female | Number | 196.21 | 264.80 | 151.71 |
|  |  | Rate | 0.07 | 0.09 | 0.05 |
| 25-29 years | Male | Number | 472.19 | 523.04 | 425.79 |
|  |  | Rate | 0.16 | 0.18 | 0.14 |
| 25-29 years | Female | Number | 369.13 | 468.54 | 300.21 |
|  |  | Rate | 0.13 | 0.16 | 0.10 |
| 30-34 years | Male | Number | 1477.09 | 1660.33 | 1306.65 |
|  |  | Rate | 0.48 | 0.54 | 0.43 |
| 30-34 years | Female | Number | 615.89 | 769.91 | 514.70 |
|  |  | Rate | 0.21 | 0.26 | 0.17 |
| 35-39 years | Male | Number | 3523.88 | 4017.51 | 3107.72 |
|  |  | Rate | 1.24 | 1.42 | 1.10 |
| 35-39 years | Female | Number | 1252.46 | 1512.63 | 1041.39 |
|  |  | Rate | 0.45 | 0.54 | 0.37 |
| 40-44 years | Male | Number | 8463.16 | 9723.11 | 7350.73 |
|  |  | Rate | 3.36 | 3.86 | 2.92 |
| 40-44 years | Female | Number | 2469.28 | 2946.45 | 2116.52 |
|  |  | Rate | 1.00 | 1.19 | 0.85 |
| 45-49 years | Male | Number | 18593.69 | 21787.57 | 15703.54 |
|  |  | Rate | 7.82 | 9.16 | 6.60 |
| 45-49 years | Female | Number | 5008.08 | 5941.86 | 4220.75 |
|  |  | Rate | 2.13 | 2.52 | 1.79 |
| 50-54 years | Male | Number | 36812.52 | 43534.17 | 30993.50 |
|  |  | Rate | 16.58 | 19.61 | 13.96 |
| 50-54 years | Female | Number | 9045.30 | 10532.71 | 7589.80 |
|  |  | Rate | 4.06 | 4.72 | 3.40 |
| 55-59 years | Male | Number | 52630.67 | 63051.04 | 44161.39 |
|  |  | Rate | 27.03 | 32.38 | 22.68 |
| 55-59 years | Female | Number | 14006.92 | 16371.17 | 11459.41 |
|  |  | Rate | 6.97 | 8.14 | 5.70 |
| 60-64 years | Male | Number | 57534.19 | 67260.03 | 49445.28 |
|  |  | Rate | 36.99 | 43.24 | 31.79 |
| 60-64 years | Female | Number | 16174.63 | 18755.71 | 12763.80 |
|  |  | Rate | 9.83 | 11.40 | 7.76 |
| 65-69 years | Male | Number | 71982.13 | 84728.14 | 60430.83 |
|  |  | Rate | 54.60 | 64.27 | 45.84 |
| 65-69 years | Female | Number | 22585.90 | 26961.04 | 16457.73 |
|  |  | Rate | 15.68 | 18.72 | 11.43 |
| 70-74 years | Male | Number | 69028.77 | 81037.78 | 58579.69 |
|  |  | Rate | 71.61 | 84.07 | 60.77 |
| 70-74 years | Female | Number | 24515.00 | 29119.28 | 17709.96 |
|  |  | Rate | 22.40 | 26.61 | 16.18 |
| 75-79 years | Male | Number | 49356.34 | 57658.15 | 41963.52 |
|  |  | Rate | 82.55 | 96.44 | 70.19 |
| 75-79 years | Female | Number | 20031.09 | 24227.05 | 14184.76 |
|  |  | Rate | 27.78 | 33.60 | 19.67 |
| 80-84 years | Male | Number | 32868.31 | 37630.87 | 28407.01 |
|  |  | Rate | 89.68 | 102.67 | 77.50 |
| 80-84 years | Female | Number | 15933.81 | 19056.32 | 11369.07 |
|  |  | Rate | 31.28 | 37.42 | 22.32 |
| 85-89 years | Male | Number | 19192.46 | 21426.62 | 16671.19 |
|  |  | Rate | 111.24 | 124.19 | 96.63 |
| 85-89 years | Female | Number | 10419.26 | 12542.34 | 7362.18 |
|  |  | Rate | 36.60 | 44.06 | 25.86 |
| 90-94 years | Male | Number | 5261.82 | 5874.50 | 4481.55 |
|  |  | Rate | 90.28 | 100.79 | 76.89 |
| 90-94 years | Female | Number | 4177.44 | 4983.58 | 2970.25 |
|  |  | Rate | 34.64 | 41.32 | 24.63 |
| 95+ years | Male | Number | 932.82 | 1046.41 | 734.67 |
|  |  | Rate | 61.69 | 69.21 | 48.59 |
| 95+ years | Female | Number | 1341.77 | 1598.37 | 941.01 |
|  |  | Rate | 34.07 | 40.59 | 23.89 |

**Supplementary Table 8 Age Patterns by Sex for the Total Number and Age-standardized Deaths Rates of Esophageal Cancer in 2021**

| **Age Group** | **Sex** | **Metric** | **Value** | **Upper** | **Lower** |
| --- | --- | --- | --- | --- | --- |
| 20-24 years | Male | Number | 231.70 | 261.02 | 209.01 |
|  |  | Rate | 0.08 | 0.09 | 0.07 |
| 20-24 years | Female | Number | 179.32 | 246.91 | 139.32 |
|  |  | Rate | 0.06 | 0.08 | 0.05 |
| 25-29 years | Male | Number | 384.47 | 424.98 | 348.42 |
|  |  | Rate | 0.13 | 0.14 | 0.12 |
| 25-29 years | Female | Number | 308.37 | 399.68 | 251.51 |
|  |  | Rate | 0.11 | 0.14 | 0.09 |
| 30-34 years | Male | Number | 1151.00 | 1292.30 | 1024.36 |
|  |  | Rate | 0.38 | 0.42 | 0.34 |
| 30-34 years | Female | Number | 485.38 | 614.68 | 404.70 |
|  |  | Rate | 0.16 | 0.21 | 0.14 |
| 35-39 years | Male | Number | 2600.01 | 2938.23 | 2311.68 |
|  |  | Rate | 0.92 | 1.04 | 0.82 |
| 35-39 years | Female | Number | 930.21 | 1135.61 | 784.89 |
|  |  | Rate | 0.33 | 0.41 | 0.28 |
| 40-44 years | Male | Number | 6505.99 | 7426.36 | 5673.20 |
|  |  | Rate | 2.58 | 2.95 | 2.25 |
| 40-44 years | Female | Number | 1888.94 | 2274.62 | 1638.82 |
|  |  | Rate | 0.76 | 0.92 | 0.66 |
| 45-49 years | Male | Number | 14428.64 | 16833.77 | 12309.28 |
|  |  | Rate | 6.07 | 7.08 | 5.17 |
| 45-49 years | Female | Number | 3827.61 | 4579.30 | 3236.21 |
|  |  | Rate | 1.62 | 1.94 | 1.37 |
| 50-54 years | Male | Number | 30550.40 | 35947.42 | 25888.29 |
|  |  | Rate | 13.76 | 16.19 | 11.66 |
| 50-54 years | Female | Number | 7223.12 | 8374.32 | 6141.81 |
|  |  | Rate | 3.24 | 3.76 | 2.75 |
| 55-59 years | Male | Number | 45379.51 | 53846.69 | 38289.85 |
|  |  | Rate | 23.30 | 27.65 | 19.66 |
| 55-59 years | Female | Number | 11625.05 | 13529.43 | 9696.30 |
|  |  | Rate | 5.78 | 6.73 | 4.82 |
| 60-64 years | Male | Number | 51277.85 | 59687.53 | 44109.91 |
|  |  | Rate | 32.97 | 38.38 | 28.36 |
| 60-64 years | Female | Number | 13909.38 | 15920.14 | 11132.16 |
|  |  | Rate | 8.45 | 9.68 | 6.77 |
| 65-69 years | Male | Number | 65702.85 | 77366.56 | 55203.52 |
|  |  | Rate | 49.84 | 58.69 | 41.87 |
| 65-69 years | Female | Number | 19731.79 | 23507.23 | 14662.22 |
|  |  | Rate | 13.70 | 16.32 | 10.18 |
| 70-74 years | Male | Number | 64970.15 | 76060.46 | 55114.60 |
|  |  | Rate | 67.40 | 78.91 | 57.18 |
| 70-74 years | Female | Number | 22463.32 | 26887.25 | 16657.06 |
|  |  | Rate | 20.52 | 24.57 | 15.22 |
| 75-79 years | Male | Number | 50165.44 | 58742.10 | 42773.72 |
|  |  | Rate | 83.91 | 98.25 | 71.54 |
| 75-79 years | Female | Number | 19999.72 | 24029.65 | 14529.12 |
|  |  | Rate | 27.74 | 33.33 | 20.15 |
| 80-84 years | Male | Number | 35625.86 | 40984.89 | 30707.58 |
|  |  | Rate | 97.20 | 111.82 | 83.78 |
| 80-84 years | Female | Number | 17063.33 | 20330.14 | 12253.74 |
|  |  | Rate | 33.50 | 39.92 | 24.06 |
| 85-89 years | Male | Number | 22268.38 | 24946.69 | 19346.89 |
|  |  | Rate | 129.07 | 144.60 | 112.14 |
| 85-89 years | Female | Number | 11797.76 | 14200.73 | 8430.25 |
|  |  | Rate | 41.44 | 49.88 | 29.61 |
| 90-94 years | Male | Number | 7218.52 | 8044.47 | 6211.70 |
|  |  | Rate | 123.85 | 138.02 | 106.57 |
| 90-94 years | Female | Number | 5563.17 | 6621.94 | 3953.91 |
|  |  | Rate | 46.13 | 54.90 | 32.78 |
| 95+ years | Male | Number | 1335.60 | 1491.20 | 1053.34 |
|  |  | Rate | 88.33 | 98.62 | 69.66 |
| 95+ years | Female | Number | 1809.06 | 2153.64 | 1265.22 |
|  |  | Rate | 45.94 | 54.69 | 32.13 |

**Supplementary Table 9 Age Patterns by Sex for the Total Number and Age-standardized DALYs of Esophageal Cancer in 2021**

| **Age Group** | **Sex** | **Metric** | **Value** | **Upper** | **Lower** |
| --- | --- | --- | --- | --- | --- |
| 20-24 years | Male | Number | 15747.89 | 17738.91 | 14202.48 |
|  |  | Rate | 5.19 | 5.85 | 4.68 |
| 20-24 years | Female | Number | 12202.62 | 16797.54 | 9477.62 |
|  |  | Rate | 4.15 | 5.72 | 3.23 |
| 25-29 years | Male | Number | 24251.19 | 26813.23 | 21959.46 |
|  |  | Rate | 8.16 | 9.02 | 7.38 |
| 25-29 years | Female | Number | 19481.47 | 25234.10 | 15896.13 |
|  |  | Rate | 6.69 | 8.67 | 5.46 |
| 30-34 years | Male | Number | 66979.80 | 75160.73 | 59660.34 |
|  |  | Rate | 21.92 | 24.60 | 19.53 |
| 30-34 years | Female | Number | 28262.76 | 35782.16 | 23575.87 |
|  |  | Rate | 9.45 | 11.97 | 7.89 |
| 35-39 years | Male | Number | 138569.22 | 156499.39 | 123050.99 |
|  |  | Rate | 48.95 | 55.29 | 43.47 |
| 35-39 years | Female | Number | 49579.91 | 60427.13 | 41787.50 |
|  |  | Rate | 17.85 | 21.75 | 15.04 |
| 40-44 years | Male | Number | 314152.16 | 358906.74 | 274147.88 |
|  |  | Rate | 124.58 | 142.33 | 108.72 |
| 40-44 years | Female | Number | 91352.19 | 109828.00 | 79293.57 |
|  |  | Rate | 36.82 | 44.27 | 31.96 |
| 45-49 years | Male | Number | 625173.47 | 729462.59 | 532993.23 |
|  |  | Rate | 262.83 | 306.67 | 224.08 |
| 45-49 years | Female | Number | 166162.42 | 198527.83 | 140478.54 |
|  |  | Rate | 70.51 | 84.25 | 59.62 |
| 50-54 years | Male | Number | 1177552.65 | 1385781.43 | 999066.04 |
|  |  | Rate | 530.48 | 624.28 | 450.07 |
| 50-54 years | Female | Number | 278640.96 | 322831.43 | 236686.77 |
|  |  | Rate | 124.98 | 144.80 | 106.16 |
| 55-59 years | Male | Number | 1537755.90 | 1822260.54 | 1298405.94 |
|  |  | Rate | 789.71 | 935.81 | 666.79 |
| 55-59 years | Female | Number | 394244.15 | 458386.06 | 329201.98 |
|  |  | Rate | 196.14 | 228.05 | 163.78 |
| 60-64 years | Male | Number | 1495364.18 | 1741291.71 | 1286466.14 |
|  |  | Rate | 961.42 | 1119.54 | 827.11 |
| 60-64 years | Female | Number | 406110.38 | 464393.61 | 325416.45 |
|  |  | Rate | 246.86 | 282.29 | 197.81 |
| 65-69 years | Male | Number | 1615093.46 | 1899467.89 | 1359167.20 |
|  |  | Rate | 1225.10 | 1440.81 | 1030.97 |
| 65-69 years | Female | Number | 485283.08 | 577911.98 | 360577.27 |
|  |  | Rate | 336.98 | 401.30 | 250.39 |
| 70-74 years | Male | Number | 1315282.69 | 1539888.75 | 1114384.40 |
|  |  | Rate | 1364.52 | 1597.54 | 1156.10 |
| 70-74 years | Female | Number | 455525.64 | 544525.21 | 338737.13 |
|  |  | Rate | 416.20 | 497.52 | 309.50 |
| 75-79 years | Male | Number | 813385.26 | 951275.89 | 691587.70 |
|  |  | Rate | 1360.48 | 1591.12 | 1156.76 |
| 75-79 years | Female | Number | 323887.57 | 387628.21 | 235398.42 |
|  |  | Rate | 449.23 | 537.64 | 326.50 |
| 80-84 years | Male | Number | 451632.18 | 519572.39 | 388895.37 |
|  |  | Rate | 1232.22 | 1417.59 | 1061.05 |
| 80-84 years | Female | Number | 216235.58 | 257529.86 | 154965.19 |
|  |  | Rate | 424.56 | 505.64 | 304.26 |
| 85-89 years | Male | Number | 224419.35 | 251527.96 | 195251.32 |
|  |  | Rate | 1300.78 | 1457.91 | 1131.72 |
| 85-89 years | Female | Number | 118953.81 | 142936.78 | 85096.55 |
|  |  | Rate | 417.83 | 502.08 | 298.91 |
| 90-94 years | Male | Number | 63361.96 | 70710.36 | 54646.85 |
|  |  | Rate | 1087.11 | 1213.18 | 937.58 |
| 90-94 years | Female | Number | 48788.74 | 57948.79 | 34766.41 |
|  |  | Rate | 404.52 | 480.47 | 288.26 |
| 95+ years | Male | Number | 10979.19 | 12275.62 | 8674.12 |
|  |  | Rate | 726.12 | 811.86 | 573.67 |
| 95+ years | Female | Number | 14853.07 | 17641.11 | 10385.45 |
|  |  | Rate | 377.15 | 447.94 | 263.71 |

**DALYs, disability-adjusted life-years.**

**Supplementary Table 10 Age Patterns by Sex for the Total Number and Age-standardized YLLs of Esophageal Cancer in 2021**

| **Age Group** | **Sex** | **Metric** | **Value** | **Upper** | **Lower** |
| --- | --- | --- | --- | --- | --- |
| 20-24 years | Male | Number | 15664.92 | 17648.58 | 14131.35 |
|  |  | Rate | 5.16 | 5.82 | 4.66 |
| 20-24 years | Female | Number | 12132.67 | 16706.43 | 9426.01 |
|  |  | Rate | 4.13 | 5.69 | 3.21 |
| 25-29 years | Male | Number | 24084.94 | 26623.24 | 21828.99 |
|  |  | Rate | 8.10 | 8.95 | 7.34 |
| 25-29 years | Female | Number | 19337.54 | 25063.08 | 15770.79 |
|  |  | Rate | 6.65 | 8.61 | 5.42 |
| 30-34 years | Male | Number | 66378.53 | 74517.75 | 59080.94 |
|  |  | Rate | 21.72 | 24.39 | 19.34 |
| 30-34 years | Female | Number | 28019.02 | 35484.41 | 23359.76 |
|  |  | Rate | 9.37 | 11.87 | 7.81 |
| 35-39 years | Male | Number | 137307.86 | 155172.41 | 122074.44 |
|  |  | Rate | 48.51 | 54.82 | 43.13 |
| 35-39 years | Female | Number | 49121.42 | 59967.16 | 41444.34 |
|  |  | Rate | 17.68 | 21.59 | 14.92 |
| 40-44 years | Male | Number | 311288.07 | 355293.52 | 271455.66 |
|  |  | Rate | 123.45 | 140.90 | 107.65 |
| 40-44 years | Female | Number | 90393.24 | 108861.64 | 78421.66 |
|  |  | Rate | 36.44 | 43.88 | 31.61 |
| 45-49 years | Male | Number | 619129.71 | 722137.59 | 528363.03 |
|  |  | Rate | 260.29 | 303.60 | 222.13 |
| 45-49 years | Female | Number | 164455.26 | 196746.49 | 139055.49 |
|  |  | Rate | 69.79 | 83.49 | 59.01 |
| 50-54 years | Male | Number | 1166080.71 | 1371913.81 | 988271.97 |
|  |  | Rate | 525.31 | 618.03 | 445.21 |
| 50-54 years | Female | Number | 275715.44 | 319669.17 | 234396.56 |
|  |  | Rate | 123.67 | 143.39 | 105.14 |
| 55-59 years | Male | Number | 1521905.94 | 1806088.50 | 1284080.72 |
|  |  | Rate | 781.57 | 927.51 | 659.43 |
| 55-59 years | Female | Number | 389723.20 | 453556.30 | 325027.24 |
|  |  | Rate | 193.89 | 225.65 | 161.70 |
| 60-64 years | Male | Number | 1478754.75 | 1720920.63 | 1272254.90 |
|  |  | Rate | 950.74 | 1106.44 | 817.98 |
| 60-64 years | Female | Number | 401175.87 | 459086.86 | 321136.63 |
|  |  | Rate | 243.86 | 279.06 | 195.21 |
| 65-69 years | Male | Number | 1595625.63 | 1878514.15 | 1341092.97 |
|  |  | Rate | 1210.34 | 1424.92 | 1017.26 |
| 65-69 years | Female | Number | 478595.72 | 569958.83 | 355782.53 |
|  |  | Rate | 332.34 | 395.78 | 247.06 |
| 70-74 years | Male | Number | 1297348.91 | 1518409.49 | 1100708.84 |
|  |  | Rate | 1345.92 | 1575.25 | 1141.92 |
| 70-74 years | Female | Number | 448634.12 | 536856.73 | 332923.32 |
|  |  | Rate | 409.91 | 490.51 | 304.18 |
| 75-79 years | Male | Number | 801290.63 | 938429.27 | 683081.64 |
|  |  | Rate | 1340.25 | 1569.63 | 1142.53 |
| 75-79 years | Female | Number | 318726.91 | 382919.42 | 231444.30 |
|  |  | Rate | 442.08 | 531.11 | 321.01 |
| 80-84 years | Male | Number | 444025.98 | 510703.87 | 382774.44 |
|  |  | Rate | 1211.47 | 1393.39 | 1044.35 |
| 80-84 years | Female | Number | 212286.92 | 252885.42 | 152504.01 |
|  |  | Rate | 416.81 | 496.52 | 299.43 |
| 85-89 years | Male | Number | 220447.00 | 246961.54 | 191533.45 |
|  |  | Rate | 1277.76 | 1431.44 | 1110.17 |
| 85-89 years | Female | Number | 116605.17 | 140375.60 | 83358.24 |
|  |  | Rate | 409.58 | 493.08 | 292.80 |
| 90-94 years | Male | Number | 62322.64 | 69459.98 | 53627.53 |
|  |  | Rate | 1069.27 | 1191.73 | 920.09 |
| 90-94 years | Female | Number | 47903.71 | 57020.75 | 34046.24 |
|  |  | Rate | 397.18 | 472.77 | 282.29 |
| 95+ years | Male | Number | 10804.07 | 12065.03 | 8533.04 |
|  |  | Rate | 714.54 | 797.93 | 564.34 |
| 95+ years | Female | Number | 14592.27 | 17364.85 | 10228.55 |
|  |  | Rate | 370.53 | 440.93 | 259.72 |

**YLLs, years of life lost.**

**Supplementary Table 11 Age Patterns by Sex for the Total Number and Age-standardized YLDs of Esophageal Cancer in 2021**

| **Age Group** | **Sex** | **Metric** | **Value** | **Upper** | **Lower** |
| --- | --- | --- | --- | --- | --- |
| 20-24 years | Male | Number | 82.97 | 110.00 | 58.79 |
|  |  | Rate | 0.03 | 0.04 | 0.02 |
| 20-24 years | Female | Number | 69.95 | 103.87 | 47.43 |
|  |  | Rate | 0.02 | 0.04 | 0.02 |
| 25-29 years | Male | Number | 166.26 | 220.56 | 118.68 |
|  |  | Rate | 0.06 | 0.07 | 0.04 |
| 25-29 years | Female | Number | 143.93 | 209.41 | 99.46 |
|  |  | Rate | 0.05 | 0.07 | 0.03 |
| 30-34 years | Male | Number | 601.27 | 812.71 | 423.27 |
|  |  | Rate | 0.20 | 0.27 | 0.14 |
| 30-34 years | Female | Number | 243.74 | 353.60 | 171.85 |
|  |  | Rate | 0.08 | 0.12 | 0.06 |
| 35-39 years | Male | Number | 1261.36 | 1684.01 | 877.43 |
|  |  | Rate | 0.45 | 0.59 | 0.31 |
| 35-39 years | Female | Number | 458.50 | 642.16 | 326.05 |
|  |  | Rate | 0.17 | 0.23 | 0.12 |
| 40-44 years | Male | Number | 2864.09 | 3877.03 | 2006.32 |
|  |  | Rate | 1.14 | 1.54 | 0.80 |
| 40-44 years | Female | Number | 958.96 | 1310.71 | 664.69 |
|  |  | Rate | 0.39 | 0.53 | 0.27 |
| 45-49 years | Male | Number | 6043.76 | 8210.33 | 4154.46 |
|  |  | Rate | 2.54 | 3.45 | 1.75 |
| 45-49 years | Female | Number | 1707.16 | 2324.42 | 1212.89 |
|  |  | Rate | 0.72 | 0.99 | 0.51 |
| 50-54 years | Male | Number | 11471.94 | 15722.86 | 7738.98 |
|  |  | Rate | 5.17 | 7.08 | 3.49 |
| 50-54 years | Female | Number | 2925.52 | 3921.70 | 2046.85 |
|  |  | Rate | 1.31 | 1.76 | 0.92 |
| 55-59 years | Male | Number | 15849.96 | 21572.08 | 10921.92 |
|  |  | Rate | 8.14 | 11.08 | 5.61 |
| 55-59 years | Female | Number | 4520.95 | 6097.75 | 3019.83 |
|  |  | Rate | 2.25 | 3.03 | 1.50 |
| 60-64 years | Male | Number | 16609.43 | 22624.75 | 11422.70 |
|  |  | Rate | 10.68 | 14.55 | 7.34 |
| 60-64 years | Female | Number | 4934.51 | 6579.13 | 3392.36 |
|  |  | Rate | 3.00 | 4.00 | 2.06 |
| 65-69 years | Male | Number | 19467.83 | 26251.03 | 13331.66 |
|  |  | Rate | 14.77 | 19.91 | 10.11 |
| 65-69 years | Female | Number | 6687.35 | 9046.31 | 4378.91 |
|  |  | Rate | 4.64 | 6.28 | 3.04 |
| 70-74 years | Male | Number | 17933.78 | 23652.66 | 12552.20 |
|  |  | Rate | 18.61 | 24.54 | 13.02 |
| 70-74 years | Female | Number | 6891.52 | 9324.98 | 4675.84 |
|  |  | Rate | 6.30 | 8.52 | 4.27 |
| 75-79 years | Male | Number | 12094.63 | 15782.19 | 8455.50 |
|  |  | Rate | 20.23 | 26.40 | 14.14 |
| 75-79 years | Female | Number | 5160.67 | 7071.23 | 3427.01 |
|  |  | Rate | 7.16 | 9.81 | 4.75 |
| 80-84 years | Male | Number | 7606.19 | 9987.73 | 5332.11 |
|  |  | Rate | 20.75 | 27.25 | 14.55 |
| 80-84 years | Female | Number | 3948.66 | 5248.84 | 2523.83 |
|  |  | Rate | 7.75 | 10.31 | 4.96 |
| 85-89 years | Male | Number | 3972.36 | 5041.95 | 2840.39 |
|  |  | Rate | 23.02 | 29.22 | 16.46 |
| 85-89 years | Female | Number | 2348.64 | 3213.89 | 1457.81 |
|  |  | Rate | 8.25 | 11.29 | 5.12 |
| 90-94 years | Male | Number | 1039.31 | 1351.74 | 717.35 |
|  |  | Rate | 17.83 | 23.19 | 12.31 |
| 90-94 years | Female | Number | 885.02 | 1203.63 | 542.92 |
|  |  | Rate | 7.34 | 9.98 | 4.50 |
| 95+ years | Male | Number | 175.11 | 236.12 | 116.91 |
|  |  | Rate | 11.58 | 15.62 | 7.73 |
| 95+ years | Female | Number | 260.80 | 368.06 | 159.10 |
|  |  | Rate | 6.62 | 9.35 | 4.04 |

**YLDs, years lived with disability.**

**Supplementary Table 12 The Age-standardised Rates of Esophageal Cancer DALYs Globally and for 21 GBD Regions by SDI from 1990 to 2021**

| **Location_name** | **Year** | **Val** | **Upper** | **Lower** |
| --- | --- | --- | --- | --- |
| **Andean Latin America** | 1990 | 48.90 | 55.55 | 42.88 |
|  | 1991 | 46.36 | 52.58 | 40.57 |
|  | 1992 | 47.07 | 52.67 | 41.34 |
|  | 1993 | 47.65 | 53.56 | 41.87 |
|  | 1994 | 45.79 | 51.65 | 40.56 |
|  | 1995 | 44.01 | 49.77 | 38.76 |
|  | 1996 | 43.12 | 48.36 | 38.04 |
|  | 1997 | 43.65 | 49.18 | 38.60 |
|  | 1998 | 43.29 | 48.73 | 38.24 |
|  | 1999 | 41.25 | 46.68 | 36.34 |
|  | 2000 | 41.42 | 46.70 | 36.61 |
|  | 2001 | 40.19 | 45.83 | 35.70 |
|  | 2002 | 40.65 | 46.35 | 35.64 |
|  | 2003 | 40.26 | 45.49 | 34.83 |
|  | 2004 | 39.11 | 44.09 | 33.76 |
|  | 2005 | 37.58 | 41.98 | 32.87 |
|  | 2006 | 36.54 | 40.85 | 32.30 |
|  | 2007 | 35.66 | 40.35 | 31.40 |
|  | 2008 | 35.73 | 40.91 | 31.09 |
|  | 2009 | 36.17 | 41.69 | 31.20 |
|  | 2010 | 35.64 | 40.74 | 31.22 |
|  | 2011 | 34.62 | 40.13 | 30.16 |
|  | 2012 | 34.13 | 39.74 | 29.83 |
|  | 2013 | 33.09 | 38.64 | 28.90 |
|  | 2014 | 32.17 | 37.89 | 28.02 |
|  | 2015 | 31.57 | 36.92 | 27.40 |
|  | 2016 | 31.40 | 36.19 | 27.80 |
|  | 2017 | 32.31 | 37.13 | 28.32 |
|  | 2018 | 32.75 | 38.28 | 28.48 |
|  | 2019 | 33.94 | 40.13 | 28.93 |
|  | 2020 | 32.82 | 39.69 | 27.33 |
|  | 2021 | 32.27 | 39.76 | 26.17 |
| **Australasia** | 1990 | 98.88 | 104.06 | 93.89 |
|  | 1991 | 99.25 | 103.95 | 94.36 |
|  | 1992 | 99.02 | 103.91 | 94.31 |
|  | 1993 | 100.05 | 104.87 | 95.43 |
|  | 1994 | 100.87 | 106.28 | 96.02 |
|  | 1995 | 99.62 | 104.98 | 94.53 |
|  | 1996 | 98.47 | 103.76 | 93.14 |
|  | 1997 | 97.26 | 102.34 | 91.85 |
|  | 1998 | 95.14 | 99.93 | 89.67 |
|  | 1999 | 94.98 | 99.77 | 89.67 |
|  | 2000 | 95.08 | 99.54 | 90.12 |
|  | 2001 | 94.41 | 98.72 | 89.19 |
|  | 2002 | 93.21 | 97.19 | 88.21 |
|  | 2003 | 95.25 | 99.45 | 90.19 |
|  | 2004 | 94.87 | 98.79 | 89.71 |
|  | 2005 | 93.61 | 97.71 | 88.40 |
|  | 2006 | 93.22 | 97.32 | 87.89 |
|  | 2007 | 92.56 | 96.80 | 87.07 |
|  | 2008 | 92.35 | 96.60 | 86.82 |
|  | 2009 | 89.90 | 94.08 | 84.56 |
|  | 2010 | 88.98 | 93.49 | 83.26 |
|  | 2011 | 88.38 | 92.94 | 82.70 |
|  | 2012 | 85.57 | 89.76 | 79.90 |
|  | 2013 | 85.13 | 89.31 | 79.63 |
|  | 2014 | 83.31 | 87.52 | 78.16 |
|  | 2015 | 86.40 | 90.93 | 81.03 |
|  | 2016 | 85.33 | 90.00 | 79.72 |
|  | 2017 | 83.15 | 87.62 | 77.50 |
|  | 2018 | 80.95 | 85.57 | 75.51 |
|  | 2019 | 83.22 | 88.43 | 77.60 |
|  | 2020 | 78.52 | 83.67 | 72.63 |
|  | 2021 | 80.20 | 85.45 | 74.13 |
| **Caribbean** | 1990 | 100.04 | 106.74 | 93.47 |
|  | 1991 | 96.14 | 102.48 | 89.85 |
|  | 1992 | 95.32 | 101.99 | 89.14 |
|  | 1993 | 94.82 | 101.83 | 88.82 |
|  | 1994 | 93.04 | 99.55 | 87.44 |
|  | 1995 | 94.24 | 100.35 | 88.85 |
|  | 1996 | 92.98 | 99.22 | 87.64 |
|  | 1997 | 92.59 | 98.85 | 87.38 |
|  | 1998 | 93.22 | 99.43 | 88.00 |
|  | 1999 | 91.03 | 96.85 | 85.62 |
|  | 2000 | 88.85 | 94.86 | 83.80 |
|  | 2001 | 89.98 | 95.95 | 84.90 |
|  | 2002 | 88.05 | 94.48 | 82.67 |
|  | 2003 | 89.10 | 95.46 | 83.70 |
|  | 2004 | 87.66 | 94.17 | 82.19 |
|  | 2005 | 87.76 | 94.12 | 82.42 |
|  | 2006 | 88.17 | 95.21 | 82.31 |
|  | 2007 | 91.28 | 98.90 | 84.92 |
|  | 2008 | 90.18 | 97.52 | 83.99 |
|  | 2009 | 94.40 | 101.70 | 88.32 |
|  | 2010 | 91.74 | 98.67 | 85.40 |
|  | 2011 | 92.29 | 99.01 | 86.27 |
|  | 2012 | 95.09 | 102.19 | 88.79 |
|  | 2013 | 97.62 | 104.45 | 91.31 |
|  | 2014 | 99.41 | 106.31 | 93.18 |
|  | 2015 | 97.84 | 104.89 | 91.55 |
|  | 2016 | 96.50 | 103.62 | 89.91 |
|  | 2017 | 97.77 | 105.34 | 90.64 |
|  | 2018 | 95.78 | 103.99 | 88.12 |
|  | 2019 | 96.78 | 105.68 | 88.51 |
|  | 2020 | 96.00 | 105.14 | 87.76 |
|  | 2021 | 94.29 | 107.49 | 82.30 |
| **Central Asia** | 1990 | 344.55 | 359.79 | 328.85 |
|  | 1991 | 331.41 | 346.12 | 316.76 |
|  | 1992 | 313.15 | 326.88 | 299.03 |
|  | 1993 | 307.50 | 320.79 | 293.60 |
|  | 1994 | 296.85 | 310.31 | 282.29 |
|  | 1995 | 287.03 | 299.27 | 273.65 |
|  | 1996 | 270.73 | 282.87 | 258.22 |
|  | 1997 | 251.64 | 264.17 | 239.69 |
|  | 1998 | 241.91 | 254.46 | 229.36 |
|  | 1999 | 229.56 | 240.99 | 217.49 |
|  | 2000 | 217.34 | 229.29 | 206.59 |
|  | 2001 | 210.03 | 221.76 | 200.35 |
|  | 2002 | 206.20 | 217.87 | 195.63 |
|  | 2003 | 204.34 | 216.87 | 194.17 |
|  | 2004 | 198.56 | 209.89 | 188.60 |
|  | 2005 | 186.36 | 197.66 | 176.69 |
|  | 2006 | 170.25 | 180.60 | 161.19 |
|  | 2007 | 164.33 | 175.01 | 155.00 |
|  | 2008 | 157.45 | 167.24 | 148.86 |
|  | 2009 | 153.50 | 162.68 | 145.92 |
|  | 2010 | 152.04 | 161.25 | 144.69 |
|  | 2011 | 147.25 | 155.42 | 140.31 |
|  | 2012 | 144.85 | 152.90 | 138.05 |
|  | 2013 | 132.15 | 139.81 | 125.86 |
|  | 2014 | 129.12 | 136.38 | 122.41 |
|  | 2015 | 131.06 | 138.51 | 124.14 |
|  | 2016 | 129.89 | 138.04 | 123.05 |
|  | 2017 | 124.01 | 132.53 | 116.46 |
|  | 2018 | 120.77 | 129.37 | 113.66 |
|  | 2019 | 119.04 | 128.34 | 110.80 |
|  | 2020 | 117.24 | 127.70 | 108.16 |
|  | 2021 | 115.48 | 128.96 | 102.92 |
| **Central Europe** | 1990 | 81.56 | 85.10 | 78.47 |
|  | 1991 | 83.81 | 87.51 | 80.57 |
|  | 1992 | 84.26 | 88.03 | 80.80 |
|  | 1993 | 85.35 | 89.05 | 81.99 |
|  | 1994 | 88.22 | 91.93 | 84.84 |
|  | 1995 | 90.09 | 93.31 | 86.90 |
|  | 1996 | 90.12 | 93.20 | 87.05 |
|  | 1997 | 91.96 | 95.18 | 88.98 |
|  | 1998 | 89.98 | 92.94 | 87.07 |
|  | 1999 | 88.41 | 91.19 | 85.73 |
|  | 2000 | 86.43 | 89.26 | 83.73 |
|  | 2001 | 86.05 | 88.63 | 83.21 |
|  | 2002 | 88.24 | 90.94 | 85.42 |
|  | 2003 | 87.59 | 90.42 | 84.94 |
|  | 2004 | 86.65 | 89.50 | 84.06 |
|  | 2005 | 86.52 | 89.46 | 83.75 |
|  | 2006 | 85.68 | 88.78 | 82.82 |
|  | 2007 | 85.51 | 88.61 | 82.67 |
|  | 2008 | 82.70 | 85.82 | 79.76 |
|  | 2009 | 81.57 | 84.50 | 78.58 |
|  | 2010 | 80.27 | 83.13 | 77.48 |
|  | 2011 | 79.67 | 82.45 | 76.99 |
|  | 2012 | 79.72 | 82.29 | 77.05 |
|  | 2013 | 76.84 | 79.41 | 74.15 |
|  | 2014 | 76.04 | 78.59 | 73.34 |
|  | 2015 | 75.79 | 78.63 | 73.04 |
|  | 2016 | 74.83 | 77.60 | 72.15 |
|  | 2017 | 73.74 | 76.58 | 70.92 |
|  | 2018 | 75.64 | 78.58 | 72.61 |
|  | 2019 | 74.92 | 77.95 | 71.75 |
|  | 2020 | 73.29 | 77.14 | 69.20 |
|  | 2021 | 73.05 | 79.02 | 67.15 |
| **Central Latin America** | 1990 | 63.52 | 64.93 | 61.67 |
|  | 1991 | 62.00 | 63.46 | 60.04 |
|  | 1992 | 60.65 | 62.15 | 58.74 |
|  | 1993 | 59.74 | 61.17 | 57.96 |
|  | 1994 | 58.56 | 59.98 | 56.73 |
|  | 1995 | 57.61 | 59.17 | 55.74 |
|  | 1996 | 57.26 | 58.65 | 55.50 |
|  | 1997 | 54.02 | 55.36 | 52.30 |
|  | 1998 | 52.38 | 53.74 | 50.74 |
|  | 1999 | 50.75 | 52.02 | 49.02 |
|  | 2000 | 51.16 | 52.45 | 49.47 |
|  | 2001 | 50.41 | 51.65 | 48.87 |
|  | 2002 | 49.08 | 50.26 | 47.47 |
|  | 2003 | 49.87 | 51.06 | 48.28 |
|  | 2004 | 48.08 | 49.22 | 46.57 |
|  | 2005 | 47.03 | 48.16 | 45.61 |
|  | 2006 | 47.13 | 48.25 | 45.66 |
|  | 2007 | 45.01 | 46.11 | 43.65 |
|  | 2008 | 44.77 | 45.84 | 43.44 |
|  | 2009 | 44.52 | 45.57 | 43.22 |
|  | 2010 | 42.97 | 43.97 | 41.63 |
|  | 2011 | 40.72 | 41.69 | 39.38 |
|  | 2012 | 40.67 | 41.68 | 39.26 |
|  | 2013 | 40.47 | 41.45 | 39.04 |
|  | 2014 | 38.97 | 39.92 | 37.56 |
|  | 2015 | 38.55 | 39.46 | 37.19 |
|  | 2016 | 39.15 | 40.14 | 37.76 |
|  | 2017 | 37.69 | 38.71 | 36.31 |
|  | 2018 | 37.17 | 38.55 | 35.70 |
|  | 2019 | 37.01 | 38.61 | 35.47 |
|  | 2020 | 37.56 | 40.87 | 34.67 |
|  | 2021 | 37.71 | 42.31 | 33.67 |
| **Central Sub-Saharan Africa** | 1990 | 291.65 | 368.57 | 214.18 |
|  | 1991 | 290.28 | 367.64 | 215.36 |
|  | 1992 | 287.27 | 364.54 | 213.51 |
|  | 1993 | 284.36 | 359.85 | 210.01 |
|  | 1994 | 284.35 | 359.43 | 208.22 |
|  | 1995 | 285.08 | 357.77 | 206.87 |
|  | 1996 | 286.33 | 356.61 | 207.63 |
|  | 1997 | 278.06 | 349.02 | 200.54 |
|  | 1998 | 276.67 | 346.78 | 197.43 |
|  | 1999 | 273.93 | 344.00 | 194.83 |
|  | 2000 | 271.45 | 339.92 | 193.37 |
|  | 2001 | 266.50 | 333.56 | 189.17 |
|  | 2002 | 255.86 | 321.12 | 182.79 |
|  | 2003 | 252.75 | 317.70 | 179.34 |
|  | 2004 | 246.69 | 309.84 | 177.16 |
|  | 2005 | 240.19 | 300.37 | 174.01 |
|  | 2006 | 237.20 | 294.60 | 174.33 |
|  | 2007 | 233.78 | 288.24 | 169.70 |
|  | 2008 | 232.75 | 288.52 | 167.76 |
|  | 2009 | 231.40 | 288.38 | 167.20 |
|  | 2010 | 230.83 | 287.58 | 166.56 |
|  | 2011 | 230.81 | 289.75 | 166.55 |
|  | 2012 | 230.42 | 288.53 | 166.18 |
|  | 2013 | 229.40 | 290.81 | 166.25 |
|  | 2014 | 228.02 | 289.63 | 167.19 |
|  | 2015 | 226.58 | 289.28 | 166.78 |
|  | 2016 | 225.28 | 293.43 | 167.95 |
|  | 2017 | 224.07 | 293.58 | 165.55 |
|  | 2018 | 223.00 | 292.55 | 162.82 |
|  | 2019 | 222.14 | 291.86 | 163.33 |
|  | 2020 | 222.59 | 292.47 | 161.02 |
|  | 2021 | 221.54 | 289.26 | 161.83 |
| **East Asia** | 1990 | 638.45 | 740.63 | 532.30 |
|  | 1991 | 629.01 | 723.60 | 531.79 |
|  | 1992 | 617.78 | 714.25 | 518.96 |
|  | 1993 | 607.38 | 695.08 | 520.49 |
|  | 1994 | 596.23 | 674.78 | 508.15 |
|  | 1995 | 589.67 | 663.08 | 496.47 |
|  | 1996 | 575.48 | 643.83 | 487.36 |
|  | 1997 | 563.38 | 628.80 | 484.88 |
|  | 1998 | 556.88 | 620.69 | 471.98 |
|  | 1999 | 553.84 | 616.43 | 470.09 |
|  | 2000 | 559.42 | 626.71 | 480.44 |
|  | 2001 | 556.64 | 627.37 | 477.47 |
|  | 2002 | 549.19 | 611.02 | 468.05 |
|  | 2003 | 546.56 | 605.84 | 466.05 |
|  | 2004 | 545.43 | 606.83 | 470.99 |
|  | 2005 | 523.67 | 578.75 | 454.00 |
|  | 2006 | 484.43 | 535.83 | 419.35 |
|  | 2007 | 457.48 | 506.87 | 398.84 |
|  | 2008 | 438.83 | 485.98 | 390.47 |
|  | 2009 | 422.30 | 466.62 | 372.61 |
|  | 2010 | 406.57 | 457.25 | 352.85 |
|  | 2011 | 390.39 | 437.55 | 346.00 |
|  | 2012 | 375.14 | 424.87 | 331.25 |
|  | 2013 | 357.56 | 403.43 | 315.36 |
|  | 2014 | 340.10 | 390.11 | 292.60 |
|  | 2015 | 329.86 | 379.13 | 280.52 |
|  | 2016 | 323.74 | 377.86 | 274.50 |
|  | 2017 | 317.06 | 378.90 | 266.17 |
|  | 2018 | 315.41 | 376.73 | 256.74 |
|  | 2019 | 314.97 | 382.00 | 253.91 |
|  | 2020 | 315.05 | 380.00 | 252.82 |
|  | 2021 | 313.94 | 387.12 | 252.18 |
| **Eastern Europe** | 1990 | 119.37 | 121.72 | 116.94 |
|  | 1991 | 119.80 | 122.55 | 117.06 |
|  | 1992 | 122.29 | 124.91 | 119.84 |
|  | 1993 | 128.88 | 131.32 | 126.34 |
|  | 1994 | 128.31 | 130.83 | 125.73 |
|  | 1995 | 119.54 | 121.81 | 117.07 |
|  | 1996 | 112.53 | 114.69 | 110.27 |
|  | 1997 | 105.45 | 107.49 | 103.38 |
|  | 1998 | 101.80 | 103.76 | 99.62 |
|  | 1999 | 103.38 | 105.28 | 101.31 |
|  | 2000 | 102.33 | 104.14 | 100.41 |
|  | 2001 | 99.72 | 101.41 | 97.85 |
|  | 2002 | 96.01 | 97.53 | 94.29 |
|  | 2003 | 95.04 | 96.64 | 93.32 |
|  | 2004 | 93.24 | 94.86 | 91.51 |
|  | 2005 | 94.00 | 95.67 | 92.22 |
|  | 2006 | 88.30 | 89.82 | 86.66 |
|  | 2007 | 87.36 | 88.91 | 85.69 |
|  | 2008 | 88.69 | 90.17 | 87.03 |
|  | 2009 | 85.52 | 86.95 | 84.05 |
|  | 2010 | 87.43 | 88.95 | 86.00 |
|  | 2011 | 84.05 | 85.58 | 82.53 |
|  | 2012 | 82.34 | 83.91 | 80.80 |
|  | 2013 | 82.59 | 84.06 | 81.02 |
|  | 2014 | 83.27 | 84.67 | 81.73 |
|  | 2015 | 84.19 | 85.76 | 82.60 |
|  | 2016 | 83.81 | 85.38 | 82.22 |
|  | 2017 | 81.87 | 84.37 | 79.18 |
|  | 2018 | 83.50 | 86.68 | 80.31 |
|  | 2019 | 84.27 | 88.15 | 80.73 |
|  | 2020 | 82.54 | 87.30 | 78.23 |
|  | 2021 | 81.25 | 88.53 | 73.21 |
| **Eastern Sub-Saharan Africa** | 1990 | 373.02 | 429.13 | 305.58 |
|  | 1991 | 373.96 | 429.05 | 305.84 |
|  | 1992 | 374.44 | 427.99 | 310.07 |
|  | 1993 | 376.47 | 429.61 | 308.72 |
|  | 1994 | 375.98 | 425.63 | 311.03 |
|  | 1995 | 374.95 | 423.73 | 309.30 |
|  | 1996 | 373.21 | 420.34 | 310.11 |
|  | 1997 | 372.96 | 419.17 | 310.41 |
|  | 1998 | 373.42 | 418.74 | 314.63 |
|  | 1999 | 369.14 | 415.65 | 308.39 |
|  | 2000 | 367.09 | 412.83 | 307.30 |
|  | 2001 | 360.37 | 409.78 | 302.39 |
|  | 2002 | 355.56 | 404.09 | 297.91 |
|  | 2003 | 352.11 | 400.92 | 298.56 |
|  | 2004 | 348.09 | 396.53 | 296.11 |
|  | 2005 | 343.28 | 391.22 | 291.05 |
|  | 2006 | 337.40 | 386.27 | 286.92 |
|  | 2007 | 330.59 | 377.10 | 284.74 |
|  | 2008 | 324.60 | 370.14 | 277.58 |
|  | 2009 | 320.47 | 366.62 | 276.82 |
|  | 2010 | 317.61 | 364.59 | 275.50 |
|  | 2011 | 312.85 | 358.94 | 270.88 |
|  | 2012 | 308.39 | 356.71 | 267.35 |
|  | 2013 | 304.83 | 352.93 | 261.74 |
|  | 2014 | 301.72 | 351.71 | 258.66 |
|  | 2015 | 299.68 | 352.96 | 253.88 |
|  | 2016 | 298.18 | 350.97 | 251.09 |
|  | 2017 | 296.79 | 350.63 | 248.12 |
|  | 2018 | 295.78 | 353.90 | 249.36 |
|  | 2019 | 295.03 | 355.00 | 249.43 |
|  | 2020 | 294.09 | 353.49 | 249.71 |
|  | 2021 | 292.22 | 352.18 | 243.43 |
| **High-income Asia Pacific** | 1990 | 123.39 | 129.46 | 118.12 |
|  | 1991 | 124.30 | 130.43 | 118.83 |
|  | 1992 | 124.29 | 129.96 | 118.78 |
|  | 1993 | 123.40 | 128.98 | 117.88 |
|  | 1994 | 121.63 | 126.64 | 116.21 |
|  | 1995 | 119.70 | 124.03 | 114.32 |
|  | 1996 | 119.78 | 124.15 | 114.64 |
|  | 1997 | 118.73 | 122.87 | 113.53 |
|  | 1998 | 118.02 | 122.20 | 112.99 |
|  | 1999 | 117.57 | 121.64 | 112.42 |
|  | 2000 | 116.22 | 120.30 | 111.21 |
|  | 2001 | 115.38 | 119.38 | 110.48 |
|  | 2002 | 112.01 | 115.86 | 106.95 |
|  | 2003 | 111.09 | 114.94 | 106.09 |
|  | 2004 | 108.29 | 112.15 | 103.35 |
|  | 2005 | 106.10 | 110.11 | 101.21 |
|  | 2006 | 103.90 | 107.95 | 99.44 |
|  | 2007 | 102.83 | 107.02 | 97.98 |
|  | 2008 | 100.24 | 104.18 | 95.49 |
|  | 2009 | 98.00 | 101.92 | 93.35 |
|  | 2010 | 96.59 | 100.48 | 91.93 |
|  | 2011 | 94.98 | 98.92 | 90.48 |
|  | 2012 | 90.88 | 94.74 | 86.49 |
|  | 2013 | 87.91 | 91.77 | 83.34 |
|  | 2014 | 85.68 | 89.43 | 81.15 |
|  | 2015 | 84.04 | 87.75 | 79.49 |
|  | 2016 | 81.92 | 85.46 | 77.16 |
|  | 2017 | 79.30 | 82.85 | 74.47 |
|  | 2018 | 76.92 | 80.69 | 71.99 |
|  | 2019 | 76.08 | 79.91 | 71.18 |
|  | 2020 | 74.26 | 77.93 | 69.06 |
|  | 2021 | 74.52 | 78.49 | 69.25 |
| **High-income North America** | 1990 | 94.58 | 96.39 | 91.53 |
|  | 1991 | 95.39 | 97.19 | 92.30 |
|  | 1992 | 95.75 | 97.58 | 92.56 |
|  | 1993 | 97.25 | 99.23 | 94.19 |
|  | 1994 | 98.07 | 100.10 | 94.97 |
|  | 1995 | 98.16 | 100.26 | 95.13 |
|  | 1996 | 97.79 | 99.82 | 94.58 |
|  | 1997 | 96.73 | 98.80 | 93.52 |
|  | 1998 | 97.40 | 99.47 | 94.21 |
|  | 1999 | 99.44 | 101.61 | 96.17 |
|  | 2000 | 100.50 | 102.69 | 97.20 |
|  | 2001 | 100.80 | 102.93 | 97.53 |
|  | 2002 | 100.93 | 103.00 | 97.43 |
|  | 2003 | 100.64 | 102.77 | 97.12 |
|  | 2004 | 99.21 | 101.37 | 95.68 |
|  | 2005 | 99.67 | 101.77 | 96.20 |
|  | 2006 | 99.73 | 101.92 | 96.10 |
|  | 2007 | 97.80 | 99.99 | 94.19 |
|  | 2008 | 96.44 | 98.66 | 92.95 |
|  | 2009 | 95.60 | 97.80 | 92.17 |
|  | 2010 | 95.17 | 97.52 | 91.68 |
|  | 2011 | 94.53 | 96.88 | 91.17 |
|  | 2012 | 93.38 | 95.72 | 89.96 |
|  | 2013 | 92.01 | 94.28 | 88.55 |
|  | 2014 | 91.22 | 93.40 | 87.93 |
|  | 2015 | 90.37 | 92.59 | 86.86 |
|  | 2016 | 90.56 | 92.78 | 87.11 |
|  | 2017 | 88.86 | 90.97 | 85.56 |
|  | 2018 | 87.12 | 89.28 | 83.70 |
|  | 2019 | 87.07 | 89.17 | 83.87 |
|  | 2020 | 85.69 | 88.00 | 82.35 |
|  | 2021 | 86.13 | 88.83 | 82.45 |
| **North Africa and Middle East** | 1990 | 68.85 | 79.48 | 54.78 |
|  | 1991 | 68.34 | 78.73 | 54.32 |
|  | 1992 | 67.81 | 77.83 | 54.33 |
|  | 1993 | 67.64 | 76.72 | 53.87 |
|  | 1994 | 67.45 | 76.11 | 53.99 |
|  | 1995 | 66.82 | 75.45 | 53.59 |
|  | 1996 | 66.09 | 74.28 | 53.45 |
|  | 1997 | 65.44 | 73.47 | 52.97 |
|  | 1998 | 64.47 | 72.54 | 52.19 |
|  | 1999 | 63.61 | 71.84 | 51.78 |
|  | 2000 | 62.37 | 70.14 | 50.94 |
|  | 2001 | 61.66 | 69.16 | 50.40 |
|  | 2002 | 60.38 | 67.28 | 49.44 |
|  | 2003 | 59.49 | 66.02 | 48.58 |
|  | 2004 | 58.54 | 65.00 | 47.94 |
|  | 2005 | 57.71 | 63.87 | 47.64 |
|  | 2006 | 56.84 | 62.43 | 47.28 |
|  | 2007 | 55.69 | 61.40 | 46.68 |
|  | 2008 | 54.81 | 60.33 | 46.02 |
|  | 2009 | 54.21 | 59.75 | 45.76 |
|  | 2010 | 53.22 | 58.49 | 45.07 |
|  | 2011 | 52.24 | 57.47 | 44.21 |
|  | 2012 | 51.45 | 56.73 | 43.71 |
|  | 2013 | 50.92 | 56.22 | 43.20 |
|  | 2014 | 50.83 | 56.11 | 43.16 |
|  | 2015 | 50.87 | 56.21 | 43.44 |
|  | 2016 | 50.45 | 55.99 | 43.07 |
|  | 2017 | 49.89 | 55.53 | 42.82 |
|  | 2018 | 49.49 | 55.26 | 42.36 |
|  | 2019 | 49.53 | 55.41 | 42.39 |
|  | 2020 | 48.36 | 54.45 | 40.86 |
|  | 2021 | 48.01 | 54.41 | 40.39 |
| **Oceania** | 1990 | 56.05 | 73.41 | 42.94 |
|  | 1991 | 56.16 | 73.51 | 43.50 |
|  | 1992 | 55.72 | 72.12 | 43.26 |
|  | 1993 | 55.38 | 70.95 | 43.47 |
|  | 1994 | 54.87 | 69.60 | 43.46 |
|  | 1995 | 54.33 | 68.58 | 43.23 |
|  | 1996 | 54.25 | 67.69 | 43.81 |
|  | 1997 | 53.59 | 66.79 | 43.49 |
|  | 1998 | 52.83 | 65.71 | 42.92 |
|  | 1999 | 52.78 | 65.24 | 42.62 |
|  | 2000 | 52.87 | 64.82 | 42.96 |
|  | 2001 | 52.26 | 63.53 | 42.34 |
|  | 2002 | 51.61 | 63.01 | 42.33 |
|  | 2003 | 51.37 | 62.80 | 42.35 |
|  | 2004 | 50.77 | 61.65 | 41.73 |
|  | 2005 | 50.92 | 61.72 | 42.29 |
|  | 2006 | 51.21 | 62.81 | 42.69 |
|  | 2007 | 51.11 | 62.63 | 42.36 |
|  | 2008 | 50.61 | 62.44 | 41.95 |
|  | 2009 | 49.70 | 61.19 | 41.02 |
|  | 2010 | 49.79 | 62.47 | 41.05 |
|  | 2011 | 49.97 | 62.80 | 40.93 |
|  | 2012 | 49.30 | 61.90 | 40.24 |
|  | 2013 | 48.60 | 61.54 | 39.53 |
|  | 2014 | 48.43 | 61.03 | 39.66 |
|  | 2015 | 48.55 | 60.69 | 39.55 |
|  | 2016 | 47.87 | 59.02 | 39.00 |
|  | 2017 | 47.88 | 59.38 | 38.50 |
|  | 2018 | 47.82 | 59.77 | 38.02 |
|  | 2019 | 48.12 | 60.13 | 37.91 |
|  | 2020 | 47.68 | 60.27 | 37.40 |
|  | 2021 | 47.10 | 59.95 | 37.29 |
| **South Asia** | 1990 | 110.56 | 134.35 | 97.80 |
|  | 1991 | 110.55 | 133.72 | 97.26 |
|  | 1992 | 111.84 | 133.18 | 100.14 |
|  | 1993 | 111.49 | 133.02 | 99.95 |
|  | 1994 | 112.23 | 134.17 | 101.02 |
|  | 1995 | 113.48 | 136.47 | 101.60 |
|  | 1996 | 113.36 | 134.17 | 102.10 |
|  | 1997 | 113.81 | 132.92 | 102.78 |
|  | 1998 | 113.33 | 134.91 | 101.76 |
|  | 1999 | 110.49 | 129.13 | 99.64 |
|  | 2000 | 109.18 | 129.68 | 98.81 |
|  | 2001 | 108.03 | 127.44 | 98.12 |
|  | 2002 | 106.12 | 125.71 | 96.27 |
|  | 2003 | 104.65 | 123.20 | 94.30 |
|  | 2004 | 101.98 | 121.42 | 91.88 |
|  | 2005 | 101.53 | 121.00 | 92.12 |
|  | 2006 | 99.64 | 119.40 | 89.62 |
|  | 2007 | 99.17 | 120.33 | 90.30 |
|  | 2008 | 98.01 | 118.48 | 89.43 |
|  | 2009 | 95.58 | 113.95 | 87.10 |
|  | 2010 | 95.01 | 114.31 | 86.56 |
|  | 2011 | 93.73 | 114.26 | 85.29 |
|  | 2012 | 91.39 | 112.07 | 83.13 |
|  | 2013 | 91.56 | 113.35 | 83.21 |
|  | 2014 | 91.07 | 111.07 | 82.77 |
|  | 2015 | 91.75 | 112.62 | 83.02 |
|  | 2016 | 91.95 | 112.70 | 83.37 |
|  | 2017 | 92.60 | 112.23 | 83.50 |
|  | 2018 | 93.24 | 114.26 | 84.39 |
|  | 2019 | 92.84 | 111.41 | 83.87 |
|  | 2020 | 92.03 | 107.76 | 82.66 |
|  | 2021 | 91.08 | 108.48 | 80.54 |
| **Southeast Asia** | 1990 | 73.65 | 86.17 | 61.86 |
|  | 1991 | 73.07 | 84.88 | 61.12 |
|  | 1992 | 72.89 | 83.88 | 61.61 |
|  | 1993 | 72.94 | 83.05 | 61.67 |
|  | 1994 | 73.14 | 82.45 | 62.23 |
|  | 1995 | 73.12 | 82.42 | 63.10 |
|  | 1996 | 73.07 | 81.83 | 62.84 |
|  | 1997 | 71.30 | 79.38 | 61.30 |
|  | 1998 | 71.55 | 80.71 | 61.52 |
|  | 1999 | 72.00 | 80.08 | 62.14 |
|  | 2000 | 71.50 | 79.96 | 62.61 |
|  | 2001 | 70.24 | 78.39 | 61.28 |
|  | 2002 | 70.25 | 78.22 | 61.82 |
|  | 2003 | 70.08 | 77.65 | 61.49 |
|  | 2004 | 70.01 | 77.47 | 61.89 |
|  | 2005 | 69.80 | 77.11 | 62.46 |
|  | 2006 | 69.01 | 76.42 | 61.93 |
|  | 2007 | 68.31 | 74.61 | 61.79 |
|  | 2008 | 67.83 | 74.60 | 61.96 |
|  | 2009 | 67.03 | 73.34 | 61.33 |
|  | 2010 | 66.72 | 73.06 | 61.28 |
|  | 2011 | 65.95 | 72.78 | 60.58 |
|  | 2012 | 64.69 | 71.84 | 59.64 |
|  | 2013 | 63.97 | 70.98 | 58.46 |
|  | 2014 | 63.64 | 70.39 | 58.18 |
|  | 2015 | 63.31 | 69.67 | 58.12 |
|  | 2016 | 63.19 | 70.69 | 57.10 |
|  | 2017 | 62.65 | 70.30 | 56.79 |
|  | 2018 | 62.47 | 69.51 | 56.38 |
|  | 2019 | 62.79 | 70.57 | 56.03 |
|  | 2020 | 61.80 | 70.32 | 54.36 |
|  | 2021 | 61.71 | 70.79 | 53.19 |
| **Southern Latin America** | 1990 | 177.31 | 184.56 | 169.83 |
|  | 1991 | 174.64 | 181.99 | 167.17 |
|  | 1992 | 171.69 | 178.84 | 164.33 |
|  | 1993 | 167.93 | 175.17 | 160.85 |
|  | 1994 | 165.92 | 173.24 | 158.77 |
|  | 1995 | 159.92 | 166.69 | 152.92 |
|  | 1996 | 163.23 | 170.09 | 156.12 |
|  | 1997 | 170.50 | 177.85 | 163.15 |
|  | 1998 | 168.84 | 176.60 | 161.79 |
|  | 1999 | 169.88 | 177.75 | 163.02 |
|  | 2000 | 165.55 | 173.15 | 158.50 |
|  | 2001 | 160.54 | 167.67 | 153.46 |
|  | 2002 | 158.44 | 165.16 | 151.53 |
|  | 2003 | 155.46 | 161.63 | 148.75 |
|  | 2004 | 149.63 | 155.95 | 143.22 |
|  | 2005 | 146.54 | 153.05 | 140.03 |
|  | 2006 | 143.02 | 149.22 | 136.51 |
|  | 2007 | 140.57 | 147.01 | 134.22 |
|  | 2008 | 132.66 | 138.69 | 126.50 |
|  | 2009 | 127.60 | 133.53 | 121.79 |
|  | 2010 | 122.00 | 127.70 | 116.03 |
|  | 2011 | 120.10 | 125.41 | 113.95 |
|  | 2012 | 117.39 | 122.77 | 111.48 |
|  | 2013 | 114.16 | 119.25 | 108.44 |
|  | 2014 | 109.71 | 115.01 | 104.29 |
|  | 2015 | 106.73 | 111.65 | 101.76 |
|  | 2016 | 104.76 | 109.64 | 99.93 |
|  | 2017 | 99.81 | 104.86 | 95.04 |
|  | 2018 | 98.48 | 103.55 | 93.53 |
|  | 2019 | 97.25 | 102.49 | 92.11 |
|  | 2020 | 93.93 | 99.62 | 88.65 |
|  | 2021 | 88.92 | 94.99 | 83.56 |
| **Southern Sub-Saharan Africa** | 1990 | 318.41 | 365.34 | 285.54 |
|  | 1991 | 323.17 | 370.84 | 285.09 |
|  | 1992 | 337.15 | 381.09 | 299.78 |
|  | 1993 | 332.58 | 379.50 | 298.04 |
|  | 1994 | 350.03 | 392.51 | 318.19 |
|  | 1995 | 361.73 | 404.55 | 324.11 |
|  | 1996 | 390.00 | 429.90 | 353.11 |
|  | 1997 | 426.91 | 463.02 | 389.92 |
|  | 1998 | 445.51 | 481.52 | 403.36 |
|  | 1999 | 427.64 | 459.82 | 386.50 |
|  | 2000 | 443.36 | 474.54 | 399.40 |
|  | 2001 | 433.29 | 463.44 | 392.63 |
|  | 2002 | 436.15 | 467.06 | 393.63 |
|  | 2003 | 440.75 | 471.39 | 395.95 |
|  | 2004 | 433.27 | 463.37 | 390.33 |
|  | 2005 | 427.50 | 457.80 | 387.06 |
|  | 2006 | 422.83 | 454.01 | 379.84 |
|  | 2007 | 406.83 | 436.67 | 366.87 |
|  | 2008 | 398.35 | 427.28 | 362.16 |
|  | 2009 | 389.91 | 418.81 | 353.47 |
|  | 2010 | 377.25 | 406.71 | 345.37 |
|  | 2011 | 361.14 | 387.27 | 332.65 |
|  | 2012 | 348.58 | 373.76 | 321.00 |
|  | 2013 | 338.09 | 362.72 | 311.39 |
|  | 2014 | 334.38 | 359.32 | 309.57 |
|  | 2015 | 332.40 | 358.29 | 306.68 |
|  | 2016 | 326.55 | 352.02 | 301.40 |
|  | 2017 | 314.31 | 338.47 | 287.76 |
|  | 2018 | 308.88 | 334.08 | 284.49 |
|  | 2019 | 298.32 | 324.04 | 271.77 |
|  | 2020 | 300.32 | 327.00 | 273.01 |
|  | 2021 | 297.67 | 326.47 | 271.83 |
| **Tropical Latin America** | 1990 | 181.09 | 186.12 | 174.39 |
|  | 1991 | 177.45 | 182.45 | 170.87 |
|  | 1992 | 178.98 | 183.79 | 172.49 |
|  | 1993 | 181.65 | 186.21 | 175.62 |
|  | 1994 | 178.24 | 182.74 | 172.28 |
|  | 1995 | 175.00 | 179.48 | 169.01 |
|  | 1996 | 173.87 | 178.16 | 167.69 |
|  | 1997 | 171.71 | 176.05 | 165.77 |
|  | 1998 | 171.09 | 175.34 | 165.12 |
|  | 1999 | 170.94 | 175.10 | 165.20 |
|  | 2000 | 169.87 | 174.15 | 164.38 |
|  | 2001 | 169.15 | 173.49 | 163.21 |
|  | 2002 | 169.32 | 173.60 | 163.64 |
|  | 2003 | 169.25 | 173.39 | 164.02 |
|  | 2004 | 170.56 | 174.87 | 164.89 |
|  | 2005 | 165.32 | 169.55 | 159.97 |
|  | 2006 | 164.20 | 168.47 | 158.79 |
|  | 2007 | 162.43 | 166.56 | 156.74 |
|  | 2008 | 160.46 | 164.95 | 154.99 |
|  | 2009 | 159.34 | 163.46 | 153.74 |
|  | 2010 | 156.68 | 160.82 | 151.00 |
|  | 2011 | 153.53 | 157.75 | 147.93 |
|  | 2012 | 149.73 | 153.96 | 144.20 |
|  | 2013 | 147.30 | 151.40 | 142.16 |
|  | 2014 | 145.43 | 149.29 | 139.60 |
|  | 2015 | 143.68 | 147.59 | 138.00 |
|  | 2016 | 142.81 | 146.54 | 136.94 |
|  | 2017 | 138.17 | 142.37 | 132.72 |
|  | 2018 | 135.14 | 139.18 | 129.58 |
|  | 2019 | 133.61 | 137.62 | 128.65 |
|  | 2020 | 133.94 | 138.37 | 128.51 |
|  | 2021 | 132.10 | 137.34 | 125.59 |
| **Western Europe** | 1990 | 121.21 | 123.91 | 118.00 |
|  | 1991 | 120.15 | 122.76 | 117.00 |
|  | 1992 | 120.02 | 122.61 | 116.86 |
|  | 1993 | 120.77 | 123.34 | 117.41 |
|  | 1994 | 120.36 | 123.04 | 116.98 |
|  | 1995 | 120.07 | 122.72 | 116.64 |
|  | 1996 | 119.08 | 121.87 | 115.71 |
|  | 1997 | 116.87 | 119.62 | 113.53 |
|  | 1998 | 116.00 | 118.52 | 112.63 |
|  | 1999 | 115.22 | 117.77 | 111.69 |
|  | 2000 | 113.99 | 116.63 | 110.25 |
|  | 2001 | 113.42 | 116.07 | 109.64 |
|  | 2002 | 113.14 | 115.79 | 109.30 |
|  | 2003 | 111.85 | 114.57 | 108.18 |
|  | 2004 | 109.05 | 111.69 | 105.50 |
|  | 2005 | 108.13 | 110.72 | 104.59 |
|  | 2006 | 106.06 | 108.72 | 102.56 |
|  | 2007 | 104.76 | 107.35 | 101.13 |
|  | 2008 | 103.66 | 106.30 | 99.92 |
|  | 2009 | 101.87 | 104.49 | 98.07 |
|  | 2010 | 100.38 | 102.96 | 96.68 |
|  | 2011 | 96.76 | 99.29 | 92.97 |
|  | 2012 | 93.43 | 95.85 | 89.72 |
|  | 2013 | 91.20 | 93.49 | 87.28 |
|  | 2014 | 92.07 | 94.33 | 88.15 |
|  | 2015 | 93.31 | 95.71 | 89.41 |
|  | 2016 | 92.59 | 95.03 | 88.70 |
|  | 2017 | 91.84 | 94.44 | 88.15 |
|  | 2018 | 91.91 | 94.57 | 88.07 |
|  | 2019 | 90.56 | 93.47 | 86.45 |
|  | 2020 | 85.98 | 89.00 | 82.00 |
|  | 2021 | 85.44 | 88.58 | 81.46 |
| **Western Sub-Saharan Africa** | 1990 | 71.08 | 86.89 | 57.33 |
|  | 1991 | 70.71 | 83.88 | 58.68 |
|  | 1992 | 70.64 | 83.60 | 57.83 |
|  | 1993 | 70.49 | 83.83 | 57.11 |
|  | 1994 | 70.39 | 83.94 | 56.71 |
|  | 1995 | 70.20 | 82.77 | 57.97 |
|  | 1996 | 69.76 | 82.49 | 55.98 |
|  | 1997 | 69.67 | 82.84 | 56.92 |
|  | 1998 | 69.77 | 82.82 | 56.91 |
|  | 1999 | 70.20 | 84.68 | 57.24 |
|  | 2000 | 71.29 | 85.75 | 57.71 |
|  | 2001 | 73.84 | 88.70 | 58.90 |
|  | 2002 | 77.50 | 92.80 | 61.93 |
|  | 2003 | 80.99 | 95.77 | 63.41 |
|  | 2004 | 84.30 | 101.03 | 66.24 |
|  | 2005 | 87.07 | 104.24 | 67.58 |
|  | 2006 | 89.80 | 105.99 | 69.86 |
|  | 2007 | 92.60 | 110.66 | 70.62 |
|  | 2008 | 94.45 | 114.82 | 71.08 |
|  | 2009 | 97.15 | 114.91 | 73.47 |
|  | 2010 | 99.93 | 117.69 | 77.31 |
|  | 2011 | 102.40 | 122.26 | 78.24 |
|  | 2012 | 104.52 | 124.24 | 80.83 |
|  | 2013 | 106.36 | 125.00 | 82.77 |
|  | 2014 | 107.78 | 127.37 | 82.53 |
|  | 2015 | 107.77 | 126.99 | 82.72 |
|  | 2016 | 109.16 | 129.46 | 84.10 |
|  | 2017 | 109.60 | 129.48 | 83.69 |
|  | 2018 | 109.84 | 132.19 | 83.75 |
|  | 2019 | 110.05 | 131.84 | 81.98 |
|  | 2020 | 109.96 | 133.42 | 82.21 |
|  | 2021 | 110.00 | 132.20 | 82.38 |

**DALYs, disability-adjusted life-years; GBD, Global Burden of Disease Study; SDI, Socio-demographic index.**

**Supplementary Table 13 The Age-standardised Rates of Esophageal Cancer Incidence Globally and for 21 GBD Regions by SDI from 1990 to 2021**

| **Location_name** | **Year** | **Val** | **Upper** | **Lower** |
| --- | --- | --- | --- | --- |
| **Andean Latin America** | 1990 | 1.96 | 2.23 | 1.73 |
|  | 1991 | 1.87 | 2.11 | 1.64 |
|  | 1992 | 1.92 | 2.16 | 1.68 |
|  | 1993 | 1.90 | 2.13 | 1.67 |
|  | 1994 | 1.85 | 2.09 | 1.63 |
|  | 1995 | 1.79 | 2.00 | 1.57 |
|  | 1996 | 1.76 | 1.97 | 1.55 |
|  | 1997 | 1.79 | 2.01 | 1.59 |
|  | 1998 | 1.78 | 2.00 | 1.58 |
|  | 1999 | 1.70 | 1.91 | 1.51 |
|  | 2000 | 1.71 | 1.92 | 1.52 |
|  | 2001 | 1.67 | 1.90 | 1.48 |
|  | 2002 | 1.69 | 1.92 | 1.49 |
|  | 2003 | 1.67 | 1.88 | 1.45 |
|  | 2004 | 1.62 | 1.83 | 1.41 |
|  | 2005 | 1.56 | 1.75 | 1.37 |
|  | 2006 | 1.52 | 1.71 | 1.36 |
|  | 2007 | 1.49 | 1.67 | 1.31 |
|  | 2008 | 1.49 | 1.70 | 1.30 |
|  | 2009 | 1.51 | 1.72 | 1.31 |
|  | 2010 | 1.49 | 1.70 | 1.30 |
|  | 2011 | 1.46 | 1.68 | 1.27 |
|  | 2012 | 1.45 | 1.68 | 1.27 |
|  | 2013 | 1.41 | 1.64 | 1.23 |
|  | 2014 | 1.37 | 1.61 | 1.20 |
|  | 2015 | 1.35 | 1.57 | 1.18 |
|  | 2016 | 1.35 | 1.56 | 1.20 |
|  | 2017 | 1.39 | 1.59 | 1.23 |
|  | 2018 | 1.41 | 1.64 | 1.23 |
|  | 2019 | 1.47 | 1.73 | 1.26 |
|  | 2020 | 1.41 | 1.71 | 1.18 |
|  | 2021 | 1.38 | 1.70 | 1.14 |
| **Australasia** | 1990 | 4.47 | 4.72 | 4.23 |
|  | 1991 | 4.50 | 4.74 | 4.27 |
|  | 1992 | 4.51 | 4.74 | 4.27 |
|  | 1993 | 4.61 | 4.85 | 4.35 |
|  | 1994 | 4.67 | 4.93 | 4.41 |
|  | 1995 | 4.66 | 4.92 | 4.38 |
|  | 1996 | 4.67 | 4.93 | 4.38 |
|  | 1997 | 4.64 | 4.90 | 4.33 |
|  | 1998 | 4.57 | 4.83 | 4.26 |
|  | 1999 | 4.56 | 4.81 | 4.24 |
|  | 2000 | 4.55 | 4.79 | 4.25 |
|  | 2001 | 4.53 | 4.74 | 4.22 |
|  | 2002 | 4.49 | 4.69 | 4.20 |
|  | 2003 | 4.59 | 4.82 | 4.28 |
|  | 2004 | 4.58 | 4.79 | 4.26 |
|  | 2005 | 4.56 | 4.78 | 4.25 |
|  | 2006 | 4.56 | 4.79 | 4.24 |
|  | 2007 | 4.54 | 4.77 | 4.22 |
|  | 2008 | 4.55 | 4.80 | 4.23 |
|  | 2009 | 4.42 | 4.67 | 4.08 |
|  | 2010 | 4.35 | 4.61 | 4.01 |
|  | 2011 | 4.36 | 4.61 | 4.03 |
|  | 2012 | 4.24 | 4.47 | 3.93 |
|  | 2013 | 4.24 | 4.48 | 3.92 |
|  | 2014 | 4.17 | 4.40 | 3.87 |
|  | 2015 | 4.35 | 4.60 | 4.05 |
|  | 2016 | 4.31 | 4.56 | 3.98 |
|  | 2017 | 4.20 | 4.44 | 3.86 |
|  | 2018 | 4.11 | 4.35 | 3.78 |
|  | 2019 | 4.19 | 4.46 | 3.83 |
|  | 2020 | 3.95 | 4.22 | 3.59 |
|  | 2021 | 4.05 | 4.33 | 3.68 |
| **Caribbean** | 1990 | 3.91 | 4.14 | 3.69 |
|  | 1991 | 3.75 | 3.97 | 3.52 |
|  | 1992 | 3.73 | 3.94 | 3.51 |
|  | 1993 | 3.70 | 3.95 | 3.48 |
|  | 1994 | 3.63 | 3.86 | 3.40 |
|  | 1995 | 3.66 | 3.88 | 3.46 |
|  | 1996 | 3.63 | 3.85 | 3.42 |
|  | 1997 | 3.59 | 3.81 | 3.38 |
|  | 1998 | 3.62 | 3.85 | 3.44 |
|  | 1999 | 3.53 | 3.75 | 3.34 |
|  | 2000 | 3.44 | 3.65 | 3.25 |
|  | 2001 | 3.48 | 3.69 | 3.29 |
|  | 2002 | 3.41 | 3.64 | 3.22 |
|  | 2003 | 3.46 | 3.69 | 3.26 |
|  | 2004 | 3.40 | 3.63 | 3.21 |
|  | 2005 | 3.38 | 3.61 | 3.18 |
|  | 2006 | 3.40 | 3.64 | 3.19 |
|  | 2007 | 3.52 | 3.79 | 3.30 |
|  | 2008 | 3.49 | 3.76 | 3.27 |
|  | 2009 | 3.63 | 3.88 | 3.41 |
|  | 2010 | 3.53 | 3.78 | 3.32 |
|  | 2011 | 3.55 | 3.79 | 3.34 |
|  | 2012 | 3.65 | 3.88 | 3.43 |
|  | 2013 | 3.73 | 3.97 | 3.51 |
|  | 2014 | 3.80 | 4.05 | 3.57 |
|  | 2015 | 3.73 | 3.98 | 3.51 |
|  | 2016 | 3.68 | 3.93 | 3.45 |
|  | 2017 | 3.73 | 3.99 | 3.50 |
|  | 2018 | 3.65 | 3.93 | 3.39 |
|  | 2019 | 3.68 | 3.99 | 3.40 |
|  | 2020 | 3.66 | 3.99 | 3.38 |
|  | 2021 | 3.60 | 4.08 | 3.17 |
| **Central Asia** | 1990 | 12.77 | 13.35 | 12.15 |
|  | 1991 | 12.30 | 12.89 | 11.69 |
|  | 1992 | 11.67 | 12.20 | 11.10 |
|  | 1993 | 11.40 | 11.92 | 10.87 |
|  | 1994 | 10.96 | 11.49 | 10.42 |
|  | 1995 | 10.56 | 11.05 | 10.07 |
|  | 1996 | 9.96 | 10.42 | 9.50 |
|  | 1997 | 9.27 | 9.75 | 8.83 |
|  | 1998 | 8.94 | 9.40 | 8.50 |
|  | 1999 | 8.52 | 8.95 | 8.06 |
|  | 2000 | 8.05 | 8.51 | 7.64 |
|  | 2001 | 7.82 | 8.26 | 7.44 |
|  | 2002 | 7.71 | 8.13 | 7.31 |
|  | 2003 | 7.63 | 8.10 | 7.22 |
|  | 2004 | 7.40 | 7.82 | 7.02 |
|  | 2005 | 6.92 | 7.33 | 6.57 |
|  | 2006 | 6.36 | 6.74 | 6.01 |
|  | 2007 | 6.16 | 6.56 | 5.80 |
|  | 2008 | 5.93 | 6.31 | 5.60 |
|  | 2009 | 5.81 | 6.16 | 5.51 |
|  | 2010 | 5.78 | 6.13 | 5.48 |
|  | 2011 | 5.60 | 5.92 | 5.32 |
|  | 2012 | 5.52 | 5.85 | 5.25 |
|  | 2013 | 5.01 | 5.31 | 4.76 |
|  | 2014 | 4.91 | 5.19 | 4.65 |
|  | 2015 | 5.02 | 5.30 | 4.73 |
|  | 2016 | 4.97 | 5.27 | 4.69 |
|  | 2017 | 4.74 | 5.07 | 4.46 |
|  | 2018 | 4.61 | 4.93 | 4.33 |
|  | 2019 | 4.55 | 4.89 | 4.22 |
|  | 2020 | 4.50 | 4.87 | 4.15 |
|  | 2021 | 4.42 | 4.90 | 3.98 |
| **Central Europe** | 1990 | 2.89 | 3.00 | 2.77 |
|  | 1991 | 2.96 | 3.08 | 2.84 |
|  | 1992 | 2.97 | 3.10 | 2.84 |
|  | 1993 | 3.01 | 3.14 | 2.89 |
|  | 1994 | 3.12 | 3.24 | 2.99 |
|  | 1995 | 3.18 | 3.29 | 3.07 |
|  | 1996 | 3.18 | 3.29 | 3.07 |
|  | 1997 | 3.24 | 3.35 | 3.13 |
|  | 1998 | 3.17 | 3.27 | 3.06 |
|  | 1999 | 3.11 | 3.21 | 3.01 |
|  | 2000 | 3.06 | 3.16 | 2.95 |
|  | 2001 | 3.05 | 3.14 | 2.94 |
|  | 2002 | 3.12 | 3.21 | 3.01 |
|  | 2003 | 3.11 | 3.21 | 3.00 |
|  | 2004 | 3.08 | 3.18 | 2.99 |
|  | 2005 | 3.08 | 3.18 | 2.98 |
|  | 2006 | 3.05 | 3.16 | 2.94 |
|  | 2007 | 3.05 | 3.16 | 2.94 |
|  | 2008 | 2.96 | 3.07 | 2.85 |
|  | 2009 | 2.93 | 3.04 | 2.82 |
|  | 2010 | 2.90 | 3.01 | 2.79 |
|  | 2011 | 2.88 | 2.98 | 2.78 |
|  | 2012 | 2.90 | 3.00 | 2.79 |
|  | 2013 | 2.81 | 2.90 | 2.70 |
|  | 2014 | 2.78 | 2.88 | 2.67 |
|  | 2015 | 2.79 | 2.89 | 2.67 |
|  | 2016 | 2.76 | 2.87 | 2.65 |
|  | 2017 | 2.73 | 2.84 | 2.61 |
|  | 2018 | 2.80 | 2.91 | 2.68 |
|  | 2019 | 2.78 | 2.90 | 2.66 |
|  | 2020 | 2.74 | 2.88 | 2.57 |
|  | 2021 | 2.73 | 2.95 | 2.50 |
| **Central Latin America** | 1990 | 2.57 | 2.63 | 2.46 |
|  | 1991 | 2.51 | 2.57 | 2.40 |
|  | 1992 | 2.45 | 2.52 | 2.35 |
|  | 1993 | 2.42 | 2.49 | 2.32 |
|  | 1994 | 2.38 | 2.45 | 2.28 |
|  | 1995 | 2.35 | 2.43 | 2.26 |
|  | 1996 | 2.34 | 2.40 | 2.24 |
|  | 1997 | 2.20 | 2.27 | 2.12 |
|  | 1998 | 2.14 | 2.20 | 2.06 |
|  | 1999 | 2.08 | 2.14 | 1.99 |
|  | 2000 | 2.09 | 2.15 | 1.99 |
|  | 2001 | 2.06 | 2.12 | 1.96 |
|  | 2002 | 2.01 | 2.07 | 1.92 |
|  | 2003 | 2.05 | 2.10 | 1.95 |
|  | 2004 | 1.97 | 2.02 | 1.89 |
|  | 2005 | 1.92 | 1.97 | 1.84 |
|  | 2006 | 1.93 | 1.98 | 1.84 |
|  | 2007 | 1.84 | 1.90 | 1.76 |
|  | 2008 | 1.83 | 1.88 | 1.74 |
|  | 2009 | 1.82 | 1.87 | 1.74 |
|  | 2010 | 1.76 | 1.80 | 1.67 |
|  | 2011 | 1.67 | 1.71 | 1.59 |
|  | 2012 | 1.66 | 1.71 | 1.58 |
|  | 2013 | 1.66 | 1.70 | 1.58 |
|  | 2014 | 1.59 | 1.64 | 1.52 |
|  | 2015 | 1.58 | 1.62 | 1.50 |
|  | 2016 | 1.61 | 1.66 | 1.53 |
|  | 2017 | 1.54 | 1.59 | 1.47 |
|  | 2018 | 1.52 | 1.58 | 1.44 |
|  | 2019 | 1.51 | 1.58 | 1.44 |
|  | 2020 | 1.53 | 1.66 | 1.41 |
|  | 2021 | 1.54 | 1.73 | 1.37 |
| **Central Sub-Saharan Africa** | 1990 | 10.59 | 13.19 | 7.92 |
|  | 1991 | 10.53 | 13.11 | 7.93 |
|  | 1992 | 10.43 | 13.03 | 7.87 |
|  | 1993 | 10.33 | 12.88 | 7.69 |
|  | 1994 | 10.33 | 12.88 | 7.65 |
|  | 1995 | 10.35 | 12.81 | 7.61 |
|  | 1996 | 10.40 | 12.93 | 7.63 |
|  | 1997 | 10.13 | 12.64 | 7.33 |
|  | 1998 | 10.07 | 12.52 | 7.25 |
|  | 1999 | 9.96 | 12.35 | 7.15 |
|  | 2000 | 9.87 | 12.20 | 7.05 |
|  | 2001 | 9.70 | 11.99 | 6.89 |
|  | 2002 | 9.34 | 11.54 | 6.73 |
|  | 2003 | 9.23 | 11.40 | 6.65 |
|  | 2004 | 9.03 | 11.26 | 6.56 |
|  | 2005 | 8.81 | 10.96 | 6.46 |
|  | 2006 | 8.71 | 10.83 | 6.46 |
|  | 2007 | 8.60 | 10.61 | 6.31 |
|  | 2008 | 8.57 | 10.58 | 6.28 |
|  | 2009 | 8.53 | 10.58 | 6.27 |
|  | 2010 | 8.52 | 10.55 | 6.23 |
|  | 2011 | 8.52 | 10.64 | 6.21 |
|  | 2012 | 8.51 | 10.65 | 6.21 |
|  | 2013 | 8.48 | 10.62 | 6.22 |
|  | 2014 | 8.43 | 10.63 | 6.19 |
|  | 2015 | 8.40 | 10.66 | 6.21 |
|  | 2016 | 8.36 | 10.76 | 6.21 |
|  | 2017 | 8.32 | 10.69 | 6.17 |
|  | 2018 | 8.29 | 10.78 | 6.08 |
|  | 2019 | 8.27 | 10.76 | 6.07 |
|  | 2020 | 8.30 | 10.78 | 6.02 |
|  | 2021 | 8.26 | 10.61 | 6.03 |
| **East Asia** | 1990 | 24.23 | 28.01 | 20.24 |
|  | 1991 | 23.93 | 27.47 | 20.19 |
|  | 1992 | 23.58 | 27.10 | 19.90 |
|  | 1993 | 23.29 | 26.43 | 19.72 |
|  | 1994 | 22.98 | 25.81 | 19.51 |
|  | 1995 | 22.83 | 25.49 | 19.03 |
|  | 1996 | 22.38 | 24.98 | 18.86 |
|  | 1997 | 22.03 | 24.56 | 18.79 |
|  | 1998 | 21.89 | 24.35 | 18.28 |
|  | 1999 | 21.89 | 24.39 | 18.29 |
|  | 2000 | 22.29 | 24.99 | 18.95 |
|  | 2001 | 22.37 | 25.13 | 18.95 |
|  | 2002 | 22.21 | 24.71 | 18.70 |
|  | 2003 | 22.37 | 24.74 | 18.64 |
|  | 2004 | 22.56 | 25.06 | 19.08 |
|  | 2005 | 21.92 | 24.29 | 18.64 |
|  | 2006 | 20.40 | 22.61 | 17.38 |
|  | 2007 | 19.46 | 21.62 | 16.63 |
|  | 2008 | 18.88 | 20.90 | 16.54 |
|  | 2009 | 18.39 | 20.34 | 15.99 |
|  | 2010 | 17.91 | 20.10 | 15.29 |
|  | 2011 | 17.34 | 19.36 | 15.24 |
|  | 2012 | 16.75 | 18.96 | 14.68 |
|  | 2013 | 16.12 | 18.04 | 14.21 |
|  | 2014 | 15.45 | 17.67 | 13.44 |
|  | 2015 | 15.05 | 17.26 | 12.96 |
|  | 2016 | 14.84 | 17.24 | 12.54 |
|  | 2017 | 14.64 | 17.40 | 12.27 |
|  | 2018 | 14.68 | 17.45 | 12.02 |
|  | 2019 | 14.76 | 17.73 | 12.16 |
|  | 2020 | 14.83 | 17.76 | 12.07 |
|  | 2021 | 14.83 | 18.09 | 11.94 |
| **Eastern Europe** | 1990 | 4.36 | 4.44 | 4.25 |
|  | 1991 | 4.38 | 4.47 | 4.27 |
|  | 1992 | 4.47 | 4.56 | 4.37 |
|  | 1993 | 4.69 | 4.77 | 4.58 |
|  | 1994 | 4.64 | 4.73 | 4.54 |
|  | 1995 | 4.33 | 4.41 | 4.24 |
|  | 1996 | 4.07 | 4.15 | 3.99 |
|  | 1997 | 3.84 | 3.91 | 3.75 |
|  | 1998 | 3.71 | 3.78 | 3.63 |
|  | 1999 | 3.76 | 3.83 | 3.69 |
|  | 2000 | 3.72 | 3.79 | 3.64 |
|  | 2001 | 3.63 | 3.69 | 3.55 |
|  | 2002 | 3.49 | 3.55 | 3.42 |
|  | 2003 | 3.45 | 3.51 | 3.39 |
|  | 2004 | 3.40 | 3.46 | 3.33 |
|  | 2005 | 3.44 | 3.50 | 3.37 |
|  | 2006 | 3.24 | 3.30 | 3.18 |
|  | 2007 | 3.21 | 3.27 | 3.15 |
|  | 2008 | 3.27 | 3.33 | 3.20 |
|  | 2009 | 3.17 | 3.22 | 3.11 |
|  | 2010 | 3.25 | 3.31 | 3.19 |
|  | 2011 | 3.12 | 3.18 | 3.07 |
|  | 2012 | 3.07 | 3.13 | 3.01 |
|  | 2013 | 3.09 | 3.15 | 3.03 |
|  | 2014 | 3.12 | 3.17 | 3.05 |
|  | 2015 | 3.17 | 3.24 | 3.11 |
|  | 2016 | 3.17 | 3.23 | 3.10 |
|  | 2017 | 3.10 | 3.19 | 3.00 |
|  | 2018 | 3.17 | 3.29 | 3.05 |
|  | 2019 | 3.20 | 3.34 | 3.06 |
|  | 2020 | 3.13 | 3.30 | 2.97 |
|  | 2021 | 3.09 | 3.35 | 2.79 |
| **Eastern Sub-Saharan Africa** | 1990 | 13.56 | 15.62 | 11.19 |
|  | 1991 | 13.58 | 15.59 | 11.18 |
|  | 1992 | 13.60 | 15.57 | 11.26 |
|  | 1993 | 13.66 | 15.54 | 11.26 |
|  | 1994 | 13.63 | 15.46 | 11.32 |
|  | 1995 | 13.60 | 15.37 | 11.23 |
|  | 1996 | 13.54 | 15.27 | 11.26 |
|  | 1997 | 13.53 | 15.16 | 11.21 |
|  | 1998 | 13.54 | 15.15 | 11.42 |
|  | 1999 | 13.40 | 15.06 | 11.25 |
|  | 2000 | 13.35 | 14.97 | 11.19 |
|  | 2001 | 13.12 | 14.87 | 10.99 |
|  | 2002 | 12.97 | 14.71 | 10.97 |
|  | 2003 | 12.86 | 14.61 | 10.97 |
|  | 2004 | 12.73 | 14.50 | 10.91 |
|  | 2005 | 12.58 | 14.38 | 10.69 |
|  | 2006 | 12.39 | 14.17 | 10.45 |
|  | 2007 | 12.14 | 13.84 | 10.33 |
|  | 2008 | 11.95 | 13.64 | 10.19 |
|  | 2009 | 11.81 | 13.50 | 10.14 |
|  | 2010 | 11.74 | 13.58 | 10.18 |
|  | 2011 | 11.57 | 13.35 | 10.05 |
|  | 2012 | 11.41 | 13.23 | 9.88 |
|  | 2013 | 11.30 | 13.08 | 9.72 |
|  | 2014 | 11.19 | 13.08 | 9.56 |
|  | 2015 | 11.14 | 13.10 | 9.46 |
|  | 2016 | 11.09 | 13.01 | 9.28 |
|  | 2017 | 11.05 | 13.08 | 9.23 |
|  | 2018 | 11.03 | 13.19 | 9.25 |
|  | 2019 | 11.02 | 13.15 | 9.29 |
|  | 2020 | 11.00 | 13.17 | 9.38 |
|  | 2021 | 10.93 | 13.09 | 9.14 |
| **High-income Asia Pacific** | 1990 | 6.53 | 6.81 | 6.23 |
|  | 1991 | 6.60 | 6.89 | 6.28 |
|  | 1992 | 6.66 | 6.93 | 6.33 |
|  | 1993 | 6.68 | 6.94 | 6.34 |
|  | 1994 | 6.70 | 6.95 | 6.34 |
|  | 1995 | 6.75 | 7.00 | 6.39 |
|  | 1996 | 6.90 | 7.16 | 6.53 |
|  | 1997 | 6.95 | 7.20 | 6.56 |
|  | 1998 | 6.99 | 7.23 | 6.60 |
|  | 1999 | 7.02 | 7.26 | 6.65 |
|  | 2000 | 6.97 | 7.23 | 6.58 |
|  | 2001 | 6.96 | 7.20 | 6.57 |
|  | 2002 | 6.83 | 7.07 | 6.43 |
|  | 2003 | 6.86 | 7.11 | 6.43 |
|  | 2004 | 6.84 | 7.10 | 6.41 |
|  | 2005 | 6.88 | 7.14 | 6.43 |
|  | 2006 | 6.89 | 7.17 | 6.46 |
|  | 2007 | 6.91 | 7.20 | 6.51 |
|  | 2008 | 6.81 | 7.10 | 6.42 |
|  | 2009 | 6.71 | 7.00 | 6.33 |
|  | 2010 | 6.66 | 6.95 | 6.28 |
|  | 2011 | 6.61 | 6.89 | 6.22 |
|  | 2012 | 6.37 | 6.65 | 5.98 |
|  | 2013 | 6.22 | 6.51 | 5.83 |
|  | 2014 | 6.11 | 6.38 | 5.71 |
|  | 2015 | 6.04 | 6.32 | 5.64 |
|  | 2016 | 5.92 | 6.20 | 5.50 |
|  | 2017 | 5.75 | 6.02 | 5.33 |
|  | 2018 | 5.61 | 5.89 | 5.16 |
|  | 2019 | 5.57 | 5.86 | 5.09 |
|  | 2020 | 5.44 | 5.74 | 4.97 |
|  | 2021 | 5.49 | 5.80 | 5.00 |
| **High-income North America** | 1990 | 4.12 | 4.21 | 3.93 |
|  | 1991 | 4.17 | 4.27 | 3.98 |
|  | 1992 | 4.21 | 4.31 | 4.01 |
|  | 1993 | 4.29 | 4.40 | 4.10 |
|  | 1994 | 4.37 | 4.48 | 4.17 |
|  | 1995 | 4.41 | 4.52 | 4.21 |
|  | 1996 | 4.44 | 4.54 | 4.23 |
|  | 1997 | 4.42 | 4.54 | 4.21 |
|  | 1998 | 4.49 | 4.60 | 4.27 |
|  | 1999 | 4.60 | 4.71 | 4.37 |
|  | 2000 | 4.66 | 4.78 | 4.45 |
|  | 2001 | 4.70 | 4.82 | 4.48 |
|  | 2002 | 4.72 | 4.84 | 4.50 |
|  | 2003 | 4.71 | 4.83 | 4.49 |
|  | 2004 | 4.67 | 4.79 | 4.43 |
|  | 2005 | 4.68 | 4.81 | 4.45 |
|  | 2006 | 4.70 | 4.82 | 4.46 |
|  | 2007 | 4.63 | 4.76 | 4.40 |
|  | 2008 | 4.59 | 4.72 | 4.36 |
|  | 2009 | 4.58 | 4.71 | 4.34 |
|  | 2010 | 4.59 | 4.73 | 4.34 |
|  | 2011 | 4.56 | 4.70 | 4.32 |
|  | 2012 | 4.51 | 4.64 | 4.27 |
|  | 2013 | 4.44 | 4.57 | 4.20 |
|  | 2014 | 4.40 | 4.53 | 4.16 |
|  | 2015 | 4.37 | 4.50 | 4.12 |
|  | 2016 | 4.39 | 4.52 | 4.15 |
|  | 2017 | 4.31 | 4.43 | 4.07 |
|  | 2018 | 4.23 | 4.35 | 3.99 |
|  | 2019 | 4.23 | 4.35 | 3.99 |
|  | 2020 | 4.16 | 4.30 | 3.92 |
|  | 2021 | 4.20 | 4.36 | 3.96 |
| **North Africa and Middle East** | 1990 | 2.59 | 2.97 | 2.10 |
|  | 1991 | 2.57 | 2.92 | 2.08 |
|  | 1992 | 2.56 | 2.90 | 2.07 |
|  | 1993 | 2.56 | 2.87 | 2.07 |
|  | 1994 | 2.55 | 2.86 | 2.07 |
|  | 1995 | 2.54 | 2.84 | 2.06 |
|  | 1996 | 2.52 | 2.81 | 2.06 |
|  | 1997 | 2.50 | 2.79 | 2.05 |
|  | 1998 | 2.47 | 2.77 | 2.02 |
|  | 1999 | 2.44 | 2.74 | 2.00 |
|  | 2000 | 2.41 | 2.69 | 1.97 |
|  | 2001 | 2.39 | 2.65 | 1.96 |
|  | 2002 | 2.35 | 2.59 | 1.94 |
|  | 2003 | 2.33 | 2.56 | 1.92 |
|  | 2004 | 2.30 | 2.53 | 1.91 |
|  | 2005 | 2.27 | 2.50 | 1.90 |
|  | 2006 | 2.25 | 2.45 | 1.88 |
|  | 2007 | 2.21 | 2.42 | 1.86 |
|  | 2008 | 2.19 | 2.38 | 1.85 |
|  | 2009 | 2.17 | 2.37 | 1.85 |
|  | 2010 | 2.14 | 2.34 | 1.83 |
|  | 2011 | 2.11 | 2.30 | 1.81 |
|  | 2012 | 2.08 | 2.28 | 1.79 |
|  | 2013 | 2.07 | 2.26 | 1.78 |
|  | 2014 | 2.08 | 2.26 | 1.79 |
|  | 2015 | 2.09 | 2.29 | 1.80 |
|  | 2016 | 2.08 | 2.29 | 1.81 |
|  | 2017 | 2.06 | 2.28 | 1.80 |
|  | 2018 | 2.05 | 2.27 | 1.78 |
|  | 2019 | 2.05 | 2.28 | 1.78 |
|  | 2020 | 2.01 | 2.24 | 1.72 |
|  | 2021 | 1.99 | 2.22 | 1.71 |
| **Oceania** | 1990 | 2.11 | 2.75 | 1.65 |
|  | 1991 | 2.12 | 2.72 | 1.67 |
|  | 1992 | 2.10 | 2.69 | 1.64 |
|  | 1993 | 2.09 | 2.64 | 1.65 |
|  | 1994 | 2.07 | 2.63 | 1.65 |
|  | 1995 | 2.05 | 2.59 | 1.65 |
|  | 1996 | 2.05 | 2.56 | 1.66 |
|  | 1997 | 2.03 | 2.52 | 1.66 |
|  | 1998 | 2.00 | 2.48 | 1.63 |
|  | 1999 | 2.00 | 2.46 | 1.64 |
|  | 2000 | 2.01 | 2.45 | 1.64 |
|  | 2001 | 1.99 | 2.41 | 1.63 |
|  | 2002 | 1.97 | 2.38 | 1.62 |
|  | 2003 | 1.96 | 2.37 | 1.63 |
|  | 2004 | 1.94 | 2.33 | 1.61 |
|  | 2005 | 1.94 | 2.35 | 1.61 |
|  | 2006 | 1.95 | 2.38 | 1.63 |
|  | 2007 | 1.95 | 2.37 | 1.62 |
|  | 2008 | 1.93 | 2.35 | 1.61 |
|  | 2009 | 1.90 | 2.31 | 1.58 |
|  | 2010 | 1.90 | 2.35 | 1.58 |
|  | 2011 | 1.91 | 2.37 | 1.58 |
|  | 2012 | 1.88 | 2.33 | 1.54 |
|  | 2013 | 1.86 | 2.33 | 1.52 |
|  | 2014 | 1.85 | 2.30 | 1.52 |
|  | 2015 | 1.85 | 2.27 | 1.52 |
|  | 2016 | 1.83 | 2.26 | 1.50 |
|  | 2017 | 1.84 | 2.27 | 1.48 |
|  | 2018 | 1.84 | 2.27 | 1.47 |
|  | 2019 | 1.84 | 2.29 | 1.47 |
|  | 2020 | 1.83 | 2.28 | 1.45 |
|  | 2021 | 1.81 | 2.28 | 1.43 |
| **South Asia** | 1990 | 3.93 | 4.75 | 3.45 |
|  | 1991 | 3.93 | 4.75 | 3.44 |
|  | 1992 | 3.98 | 4.76 | 3.54 |
|  | 1993 | 3.97 | 4.75 | 3.53 |
|  | 1994 | 4.02 | 4.82 | 3.58 |
|  | 1995 | 4.08 | 4.91 | 3.62 |
|  | 1996 | 4.07 | 4.81 | 3.64 |
|  | 1997 | 4.07 | 4.75 | 3.63 |
|  | 1998 | 4.05 | 4.82 | 3.62 |
|  | 1999 | 3.93 | 4.62 | 3.51 |
|  | 2000 | 3.88 | 4.59 | 3.47 |
|  | 2001 | 3.85 | 4.58 | 3.48 |
|  | 2002 | 3.79 | 4.50 | 3.42 |
|  | 2003 | 3.75 | 4.47 | 3.34 |
|  | 2004 | 3.67 | 4.40 | 3.30 |
|  | 2005 | 3.66 | 4.36 | 3.31 |
|  | 2006 | 3.60 | 4.33 | 3.21 |
|  | 2007 | 3.59 | 4.36 | 3.26 |
|  | 2008 | 3.55 | 4.30 | 3.23 |
|  | 2009 | 3.45 | 4.13 | 3.15 |
|  | 2010 | 3.43 | 4.13 | 3.12 |
|  | 2011 | 3.39 | 4.15 | 3.06 |
|  | 2012 | 3.30 | 4.06 | 2.99 |
|  | 2013 | 3.32 | 4.14 | 3.01 |
|  | 2014 | 3.34 | 4.09 | 3.02 |
|  | 2015 | 3.35 | 4.12 | 3.04 |
|  | 2016 | 3.36 | 4.15 | 3.03 |
|  | 2017 | 3.39 | 4.11 | 3.03 |
|  | 2018 | 3.42 | 4.23 | 3.09 |
|  | 2019 | 3.41 | 4.13 | 3.08 |
|  | 2020 | 3.39 | 4.00 | 3.04 |
|  | 2021 | 3.36 | 4.03 | 2.95 |
| **Southeast Asia** | 1990 | 2.69 | 3.14 | 2.26 |
|  | 1991 | 2.67 | 3.10 | 2.25 |
|  | 1992 | 2.67 | 3.06 | 2.28 |
|  | 1993 | 2.67 | 3.04 | 2.28 |
|  | 1994 | 2.68 | 3.03 | 2.29 |
|  | 1995 | 2.68 | 3.01 | 2.32 |
|  | 1996 | 2.69 | 2.99 | 2.32 |
|  | 1997 | 2.63 | 2.91 | 2.28 |
|  | 1998 | 2.64 | 2.96 | 2.29 |
|  | 1999 | 2.66 | 2.96 | 2.31 |
|  | 2000 | 2.64 | 2.94 | 2.32 |
|  | 2001 | 2.60 | 2.90 | 2.28 |
|  | 2002 | 2.61 | 2.90 | 2.29 |
|  | 2003 | 2.61 | 2.88 | 2.29 |
|  | 2004 | 2.61 | 2.88 | 2.30 |
|  | 2005 | 2.62 | 2.89 | 2.34 |
|  | 2006 | 2.60 | 2.87 | 2.33 |
|  | 2007 | 2.58 | 2.82 | 2.34 |
|  | 2008 | 2.57 | 2.83 | 2.35 |
|  | 2009 | 2.55 | 2.80 | 2.33 |
|  | 2010 | 2.54 | 2.79 | 2.33 |
|  | 2011 | 2.52 | 2.77 | 2.31 |
|  | 2012 | 2.47 | 2.75 | 2.28 |
|  | 2013 | 2.45 | 2.71 | 2.25 |
|  | 2014 | 2.44 | 2.69 | 2.23 |
|  | 2015 | 2.43 | 2.67 | 2.23 |
|  | 2016 | 2.43 | 2.70 | 2.20 |
|  | 2017 | 2.42 | 2.70 | 2.19 |
|  | 2018 | 2.42 | 2.69 | 2.19 |
|  | 2019 | 2.44 | 2.74 | 2.19 |
|  | 2020 | 2.41 | 2.75 | 2.12 |
|  | 2021 | 2.42 | 2.76 | 2.11 |
| **Southern Latin America** | 1990 | 7.17 | 7.49 | 6.85 |
|  | 1991 | 7.07 | 7.38 | 6.74 |
|  | 1992 | 6.96 | 7.27 | 6.63 |
|  | 1993 | 6.82 | 7.11 | 6.49 |
|  | 1994 | 6.75 | 7.04 | 6.43 |
|  | 1995 | 6.52 | 6.80 | 6.22 |
|  | 1996 | 6.69 | 6.97 | 6.37 |
|  | 1997 | 7.00 | 7.31 | 6.67 |
|  | 1998 | 6.98 | 7.30 | 6.63 |
|  | 1999 | 7.04 | 7.37 | 6.71 |
|  | 2000 | 6.88 | 7.21 | 6.52 |
|  | 2001 | 6.68 | 6.98 | 6.35 |
|  | 2002 | 6.61 | 6.90 | 6.28 |
|  | 2003 | 6.50 | 6.77 | 6.17 |
|  | 2004 | 6.27 | 6.55 | 5.97 |
|  | 2005 | 6.16 | 6.44 | 5.84 |
|  | 2006 | 6.01 | 6.29 | 5.70 |
|  | 2007 | 5.93 | 6.22 | 5.63 |
|  | 2008 | 5.59 | 5.85 | 5.31 |
|  | 2009 | 5.40 | 5.63 | 5.11 |
|  | 2010 | 5.18 | 5.41 | 4.91 |
|  | 2011 | 5.13 | 5.36 | 4.86 |
|  | 2012 | 5.02 | 5.24 | 4.75 |
|  | 2013 | 4.87 | 5.10 | 4.60 |
|  | 2014 | 4.71 | 4.94 | 4.45 |
|  | 2015 | 4.60 | 4.81 | 4.36 |
|  | 2016 | 4.54 | 4.76 | 4.29 |
|  | 2017 | 4.34 | 4.56 | 4.10 |
|  | 2018 | 4.29 | 4.50 | 4.03 |
|  | 2019 | 4.25 | 4.46 | 3.99 |
|  | 2020 | 4.10 | 4.34 | 3.84 |
|  | 2021 | 3.89 | 4.15 | 3.62 |
| **Southern Sub-Saharan Africa** | 1990 | 11.23 | 13.05 | 9.98 |
|  | 1991 | 11.40 | 13.23 | 10.06 |
|  | 1992 | 11.90 | 13.61 | 10.60 |
|  | 1993 | 11.84 | 13.64 | 10.57 |
|  | 1994 | 12.49 | 14.13 | 11.27 |
|  | 1995 | 12.94 | 14.54 | 11.53 |
|  | 1996 | 14.07 | 15.53 | 12.72 |
|  | 1997 | 15.37 | 16.65 | 14.02 |
|  | 1998 | 16.02 | 17.30 | 14.50 |
|  | 1999 | 15.45 | 16.57 | 13.87 |
|  | 2000 | 15.96 | 17.07 | 14.26 |
|  | 2001 | 15.65 | 16.72 | 14.06 |
|  | 2002 | 15.74 | 16.84 | 14.10 |
|  | 2003 | 15.87 | 16.98 | 14.22 |
|  | 2004 | 15.56 | 16.63 | 13.93 |
|  | 2005 | 15.35 | 16.44 | 13.80 |
|  | 2006 | 15.20 | 16.31 | 13.62 |
|  | 2007 | 14.70 | 15.76 | 13.23 |
|  | 2008 | 14.42 | 15.42 | 13.05 |
|  | 2009 | 14.17 | 15.18 | 12.81 |
|  | 2010 | 13.78 | 14.82 | 12.49 |
|  | 2011 | 13.25 | 14.18 | 12.08 |
|  | 2012 | 12.82 | 13.73 | 11.67 |
|  | 2013 | 12.48 | 13.36 | 11.39 |
|  | 2014 | 12.37 | 13.28 | 11.28 |
|  | 2015 | 12.33 | 13.26 | 11.29 |
|  | 2016 | 12.13 | 13.06 | 11.13 |
|  | 2017 | 11.71 | 12.60 | 10.70 |
|  | 2018 | 11.52 | 12.40 | 10.58 |
|  | 2019 | 11.12 | 12.02 | 10.17 |
|  | 2020 | 11.16 | 12.10 | 10.16 |
|  | 2021 | 11.01 | 11.99 | 10.06 |
| **Tropical Latin America** | 1990 | 6.63 | 6.83 | 6.30 |
|  | 1991 | 6.50 | 6.70 | 6.20 |
|  | 1992 | 6.54 | 6.72 | 6.23 |
|  | 1993 | 6.63 | 6.82 | 6.33 |
|  | 1994 | 6.50 | 6.68 | 6.22 |
|  | 1995 | 6.38 | 6.56 | 6.08 |
|  | 1996 | 6.32 | 6.49 | 6.02 |
|  | 1997 | 6.24 | 6.41 | 5.96 |
|  | 1998 | 6.22 | 6.39 | 5.94 |
|  | 1999 | 6.22 | 6.39 | 5.95 |
|  | 2000 | 6.16 | 6.34 | 5.90 |
|  | 2001 | 6.14 | 6.33 | 5.89 |
|  | 2002 | 6.16 | 6.33 | 5.90 |
|  | 2003 | 6.18 | 6.36 | 5.93 |
|  | 2004 | 6.22 | 6.40 | 5.97 |
|  | 2005 | 6.04 | 6.21 | 5.79 |
|  | 2006 | 6.01 | 6.18 | 5.74 |
|  | 2007 | 5.94 | 6.10 | 5.67 |
|  | 2008 | 5.86 | 6.04 | 5.59 |
|  | 2009 | 5.81 | 5.98 | 5.56 |
|  | 2010 | 5.74 | 5.92 | 5.48 |
|  | 2011 | 5.64 | 5.82 | 5.37 |
|  | 2012 | 5.53 | 5.70 | 5.26 |
|  | 2013 | 5.44 | 5.61 | 5.17 |
|  | 2014 | 5.39 | 5.55 | 5.11 |
|  | 2015 | 5.34 | 5.51 | 5.06 |
|  | 2016 | 5.31 | 5.46 | 5.02 |
|  | 2017 | 5.14 | 5.31 | 4.86 |
|  | 2018 | 5.05 | 5.22 | 4.77 |
|  | 2019 | 5.01 | 5.18 | 4.76 |
|  | 2020 | 5.01 | 5.20 | 4.75 |
|  | 2021 | 4.91 | 5.11 | 4.64 |
| **Western Europe** | 1990 | 4.92 | 5.04 | 4.75 |
|  | 1991 | 4.91 | 5.03 | 4.74 |
|  | 1992 | 4.93 | 5.05 | 4.76 |
|  | 1993 | 4.99 | 5.11 | 4.82 |
|  | 1994 | 5.01 | 5.14 | 4.85 |
|  | 1995 | 5.05 | 5.17 | 4.88 |
|  | 1996 | 5.07 | 5.19 | 4.89 |
|  | 1997 | 5.02 | 5.15 | 4.83 |
|  | 1998 | 5.01 | 5.14 | 4.82 |
|  | 1999 | 5.00 | 5.13 | 4.80 |
|  | 2000 | 4.96 | 5.09 | 4.75 |
|  | 2001 | 4.96 | 5.09 | 4.75 |
|  | 2002 | 4.97 | 5.11 | 4.75 |
|  | 2003 | 4.95 | 5.08 | 4.74 |
|  | 2004 | 4.87 | 5.01 | 4.67 |
|  | 2005 | 4.89 | 5.03 | 4.68 |
|  | 2006 | 4.86 | 5.00 | 4.64 |
|  | 2007 | 4.85 | 4.99 | 4.63 |
|  | 2008 | 4.84 | 4.98 | 4.61 |
|  | 2009 | 4.79 | 4.93 | 4.55 |
|  | 2010 | 4.74 | 4.89 | 4.50 |
|  | 2011 | 4.61 | 4.75 | 4.37 |
|  | 2012 | 4.49 | 4.62 | 4.25 |
|  | 2013 | 4.41 | 4.55 | 4.16 |
|  | 2014 | 4.49 | 4.63 | 4.24 |
|  | 2015 | 4.58 | 4.72 | 4.33 |
|  | 2016 | 4.57 | 4.72 | 4.32 |
|  | 2017 | 4.54 | 4.70 | 4.29 |
|  | 2018 | 4.56 | 4.71 | 4.31 |
|  | 2019 | 4.50 | 4.66 | 4.25 |
|  | 2020 | 4.30 | 4.48 | 4.05 |
|  | 2021 | 4.26 | 4.44 | 4.00 |
| **Western Sub-Saharan Africa** | 1990 | 2.65 | 3.21 | 2.16 |
|  | 1991 | 2.64 | 3.12 | 2.19 |
|  | 1992 | 2.63 | 3.11 | 2.17 |
|  | 1993 | 2.63 | 3.10 | 2.15 |
|  | 1994 | 2.62 | 3.12 | 2.13 |
|  | 1995 | 2.61 | 3.06 | 2.17 |
|  | 1996 | 2.60 | 3.06 | 2.13 |
|  | 1997 | 2.60 | 3.07 | 2.15 |
|  | 1998 | 2.60 | 3.07 | 2.14 |
|  | 1999 | 2.62 | 3.13 | 2.15 |
|  | 2000 | 2.66 | 3.18 | 2.19 |
|  | 2001 | 2.76 | 3.29 | 2.22 |
|  | 2002 | 2.90 | 3.45 | 2.35 |
|  | 2003 | 3.04 | 3.57 | 2.40 |
|  | 2004 | 3.16 | 3.76 | 2.53 |
|  | 2005 | 3.28 | 3.91 | 2.56 |
|  | 2006 | 3.40 | 3.97 | 2.65 |
|  | 2007 | 3.50 | 4.14 | 2.68 |
|  | 2008 | 3.58 | 4.30 | 2.72 |
|  | 2009 | 3.69 | 4.33 | 2.80 |
|  | 2010 | 3.80 | 4.46 | 2.94 |
|  | 2011 | 3.90 | 4.62 | 2.99 |
|  | 2012 | 3.98 | 4.69 | 3.07 |
|  | 2013 | 4.05 | 4.72 | 3.14 |
|  | 2014 | 4.10 | 4.82 | 3.15 |
|  | 2015 | 4.11 | 4.77 | 3.16 |
|  | 2016 | 4.16 | 4.90 | 3.23 |
|  | 2017 | 4.19 | 4.93 | 3.20 |
|  | 2018 | 4.21 | 5.03 | 3.19 |
|  | 2019 | 4.22 | 5.01 | 3.13 |
|  | 2020 | 4.22 | 5.07 | 3.15 |
|  | 2021 | 4.22 | 5.02 | 3.15 |

**GBD, Global Burden of Disease Study; SDI, Socio-demographic index.**

**Supplementary Table 14 The Age-standardised Rates of Esophageal Cancer Deaths Globally and for 21 GBD Regions by SDI from 1990 to 2021**

| **Location_name** | **Year** | **Val** | **Upper** | **Lower** |
| --- | --- | --- | --- | --- |
| **Andean Latin America** | 1990 | 2.18 | 2.47 | 1.91 |
|  | 1991 | 2.07 | 2.33 | 1.81 |
|  | 1992 | 2.13 | 2.39 | 1.87 |
|  | 1993 | 2.11 | 2.36 | 1.85 |
|  | 1994 | 2.05 | 2.32 | 1.81 |
|  | 1995 | 1.98 | 2.23 | 1.75 |
|  | 1996 | 1.95 | 2.19 | 1.72 |
|  | 1997 | 1.99 | 2.22 | 1.77 |
|  | 1998 | 1.98 | 2.22 | 1.76 |
|  | 1999 | 1.89 | 2.12 | 1.69 |
|  | 2000 | 1.90 | 2.13 | 1.68 |
|  | 2001 | 1.86 | 2.11 | 1.64 |
|  | 2002 | 1.88 | 2.14 | 1.66 |
|  | 2003 | 1.86 | 2.09 | 1.61 |
|  | 2004 | 1.80 | 2.03 | 1.57 |
|  | 2005 | 1.73 | 1.93 | 1.52 |
|  | 2006 | 1.69 | 1.88 | 1.51 |
|  | 2007 | 1.65 | 1.85 | 1.45 |
|  | 2008 | 1.66 | 1.88 | 1.44 |
|  | 2009 | 1.66 | 1.89 | 1.45 |
|  | 2010 | 1.65 | 1.87 | 1.44 |
|  | 2011 | 1.61 | 1.85 | 1.40 |
|  | 2012 | 1.60 | 1.85 | 1.40 |
|  | 2013 | 1.55 | 1.80 | 1.36 |
|  | 2014 | 1.52 | 1.77 | 1.33 |
|  | 2015 | 1.49 | 1.73 | 1.30 |
|  | 2016 | 1.49 | 1.71 | 1.32 |
|  | 2017 | 1.53 | 1.75 | 1.35 |
|  | 2018 | 1.55 | 1.80 | 1.35 |
|  | 2019 | 1.61 | 1.88 | 1.38 |
|  | 2020 | 1.54 | 1.86 | 1.30 |
|  | 2021 | 1.51 | 1.85 | 1.24 |
| **Australasia** | 1990 | 4.34 | 4.60 | 4.11 |
|  | 1991 | 4.36 | 4.59 | 4.12 |
|  | 1992 | 4.36 | 4.59 | 4.13 |
|  | 1993 | 4.45 | 4.70 | 4.19 |
|  | 1994 | 4.48 | 4.74 | 4.22 |
|  | 1995 | 4.42 | 4.68 | 4.15 |
|  | 1996 | 4.40 | 4.66 | 4.12 |
|  | 1997 | 4.35 | 4.60 | 4.05 |
|  | 1998 | 4.28 | 4.52 | 3.98 |
|  | 1999 | 4.26 | 4.49 | 3.97 |
|  | 2000 | 4.25 | 4.48 | 3.97 |
|  | 2001 | 4.23 | 4.45 | 3.94 |
|  | 2002 | 4.19 | 4.39 | 3.90 |
|  | 2003 | 4.28 | 4.50 | 3.97 |
|  | 2004 | 4.24 | 4.45 | 3.94 |
|  | 2005 | 4.21 | 4.42 | 3.89 |
|  | 2006 | 4.18 | 4.39 | 3.87 |
|  | 2007 | 4.15 | 4.37 | 3.85 |
|  | 2008 | 4.16 | 4.38 | 3.83 |
|  | 2009 | 4.03 | 4.24 | 3.69 |
|  | 2010 | 3.96 | 4.18 | 3.63 |
|  | 2011 | 3.96 | 4.17 | 3.63 |
|  | 2012 | 3.84 | 4.04 | 3.52 |
|  | 2013 | 3.82 | 4.04 | 3.50 |
|  | 2014 | 3.76 | 3.97 | 3.46 |
|  | 2015 | 3.93 | 4.15 | 3.64 |
|  | 2016 | 3.89 | 4.11 | 3.58 |
|  | 2017 | 3.79 | 4.00 | 3.46 |
|  | 2018 | 3.72 | 3.94 | 3.39 |
|  | 2019 | 3.80 | 4.03 | 3.45 |
|  | 2020 | 3.58 | 3.83 | 3.24 |
|  | 2021 | 3.68 | 3.94 | 3.33 |
| **Caribbean** | 1990 | 4.18 | 4.43 | 3.95 |
|  | 1991 | 4.00 | 4.23 | 3.77 |
|  | 1992 | 3.97 | 4.20 | 3.74 |
|  | 1993 | 3.94 | 4.20 | 3.70 |
|  | 1994 | 3.86 | 4.10 | 3.63 |
|  | 1995 | 3.89 | 4.12 | 3.67 |
|  | 1996 | 3.84 | 4.07 | 3.63 |
|  | 1997 | 3.79 | 4.02 | 3.58 |
|  | 1998 | 3.82 | 4.05 | 3.62 |
|  | 1999 | 3.72 | 3.95 | 3.52 |
|  | 2000 | 3.61 | 3.84 | 3.41 |
|  | 2001 | 3.64 | 3.88 | 3.45 |
|  | 2002 | 3.58 | 3.82 | 3.38 |
|  | 2003 | 3.63 | 3.86 | 3.41 |
|  | 2004 | 3.56 | 3.80 | 3.35 |
|  | 2005 | 3.53 | 3.77 | 3.32 |
|  | 2006 | 3.53 | 3.78 | 3.31 |
|  | 2007 | 3.65 | 3.93 | 3.43 |
|  | 2008 | 3.62 | 3.91 | 3.40 |
|  | 2009 | 3.76 | 4.02 | 3.54 |
|  | 2010 | 3.65 | 3.92 | 3.44 |
|  | 2011 | 3.67 | 3.92 | 3.46 |
|  | 2012 | 3.76 | 4.01 | 3.54 |
|  | 2013 | 3.84 | 4.09 | 3.61 |
|  | 2014 | 3.91 | 4.17 | 3.68 |
|  | 2015 | 3.84 | 4.09 | 3.60 |
|  | 2016 | 3.78 | 4.03 | 3.55 |
|  | 2017 | 3.83 | 4.10 | 3.58 |
|  | 2018 | 3.73 | 4.02 | 3.47 |
|  | 2019 | 3.76 | 4.08 | 3.47 |
|  | 2020 | 3.75 | 4.08 | 3.45 |
|  | 2021 | 3.68 | 4.16 | 3.24 |
| **Central Asia** | 1990 | 13.68 | 14.33 | 13.00 |
|  | 1991 | 13.19 | 13.82 | 12.52 |
|  | 1992 | 12.53 | 13.12 | 11.90 |
|  | 1993 | 12.23 | 12.77 | 11.64 |
|  | 1994 | 11.75 | 12.32 | 11.15 |
|  | 1995 | 11.31 | 11.83 | 10.77 |
|  | 1996 | 10.67 | 11.17 | 10.16 |
|  | 1997 | 9.93 | 10.46 | 9.45 |
|  | 1998 | 9.59 | 10.07 | 9.12 |
|  | 1999 | 9.14 | 9.61 | 8.66 |
|  | 2000 | 8.63 | 9.12 | 8.19 |
|  | 2001 | 8.40 | 8.87 | 7.97 |
|  | 2002 | 8.28 | 8.74 | 7.85 |
|  | 2003 | 8.20 | 8.70 | 7.74 |
|  | 2004 | 7.93 | 8.37 | 7.53 |
|  | 2005 | 7.40 | 7.84 | 7.03 |
|  | 2006 | 6.82 | 7.23 | 6.47 |
|  | 2007 | 6.60 | 7.04 | 6.22 |
|  | 2008 | 6.37 | 6.78 | 6.01 |
|  | 2009 | 6.25 | 6.62 | 5.92 |
|  | 2010 | 6.22 | 6.60 | 5.90 |
|  | 2011 | 6.03 | 6.39 | 5.72 |
|  | 2012 | 5.94 | 6.29 | 5.64 |
|  | 2013 | 5.38 | 5.70 | 5.11 |
|  | 2014 | 5.27 | 5.58 | 4.98 |
|  | 2015 | 5.39 | 5.71 | 5.08 |
|  | 2016 | 5.33 | 5.66 | 5.03 |
|  | 2017 | 5.09 | 5.45 | 4.79 |
|  | 2018 | 4.94 | 5.28 | 4.63 |
|  | 2019 | 4.87 | 5.25 | 4.51 |
|  | 2020 | 4.83 | 5.23 | 4.45 |
|  | 2021 | 4.74 | 5.25 | 4.28 |
| **Central Europe** | 1990 | 3.02 | 3.13 | 2.90 |
|  | 1991 | 3.09 | 3.22 | 2.97 |
|  | 1992 | 3.09 | 3.22 | 2.96 |
|  | 1993 | 3.14 | 3.27 | 3.01 |
|  | 1994 | 3.24 | 3.36 | 3.11 |
|  | 1995 | 3.30 | 3.42 | 3.19 |
|  | 1996 | 3.29 | 3.40 | 3.18 |
|  | 1997 | 3.34 | 3.45 | 3.23 |
|  | 1998 | 3.26 | 3.36 | 3.15 |
|  | 1999 | 3.20 | 3.30 | 3.09 |
|  | 2000 | 3.14 | 3.25 | 3.03 |
|  | 2001 | 3.13 | 3.23 | 3.02 |
|  | 2002 | 3.19 | 3.29 | 3.09 |
|  | 2003 | 3.18 | 3.28 | 3.07 |
|  | 2004 | 3.15 | 3.26 | 3.05 |
|  | 2005 | 3.14 | 3.24 | 3.03 |
|  | 2006 | 3.11 | 3.22 | 2.99 |
|  | 2007 | 3.10 | 3.21 | 2.99 |
|  | 2008 | 3.01 | 3.12 | 2.90 |
|  | 2009 | 2.98 | 3.09 | 2.87 |
|  | 2010 | 2.94 | 3.05 | 2.83 |
|  | 2011 | 2.93 | 3.03 | 2.81 |
|  | 2012 | 2.94 | 3.04 | 2.83 |
|  | 2013 | 2.85 | 2.95 | 2.74 |
|  | 2014 | 2.82 | 2.92 | 2.70 |
|  | 2015 | 2.82 | 2.93 | 2.71 |
|  | 2016 | 2.80 | 2.90 | 2.69 |
|  | 2017 | 2.76 | 2.87 | 2.65 |
|  | 2018 | 2.83 | 2.95 | 2.71 |
|  | 2019 | 2.82 | 2.93 | 2.69 |
|  | 2020 | 2.78 | 2.93 | 2.61 |
|  | 2021 | 2.76 | 2.98 | 2.54 |
| **Central Latin America** | 1990 | 2.84 | 2.91 | 2.72 |
|  | 1991 | 2.76 | 2.84 | 2.64 |
|  | 1992 | 2.71 | 2.78 | 2.59 |
|  | 1993 | 2.67 | 2.75 | 2.55 |
|  | 1994 | 2.63 | 2.70 | 2.50 |
|  | 1995 | 2.59 | 2.67 | 2.48 |
|  | 1996 | 2.57 | 2.64 | 2.46 |
|  | 1997 | 2.42 | 2.48 | 2.31 |
|  | 1998 | 2.35 | 2.41 | 2.25 |
|  | 1999 | 2.28 | 2.34 | 2.16 |
|  | 2000 | 2.28 | 2.35 | 2.17 |
|  | 2001 | 2.25 | 2.31 | 2.14 |
|  | 2002 | 2.20 | 2.26 | 2.09 |
|  | 2003 | 2.23 | 2.30 | 2.12 |
|  | 2004 | 2.15 | 2.21 | 2.05 |
|  | 2005 | 2.09 | 2.15 | 2.00 |
|  | 2006 | 2.10 | 2.16 | 2.00 |
|  | 2007 | 2.01 | 2.07 | 1.91 |
|  | 2008 | 1.98 | 2.04 | 1.89 |
|  | 2009 | 1.97 | 2.03 | 1.88 |
|  | 2010 | 1.90 | 1.96 | 1.81 |
|  | 2011 | 1.80 | 1.86 | 1.71 |
|  | 2012 | 1.80 | 1.85 | 1.70 |
|  | 2013 | 1.79 | 1.85 | 1.70 |
|  | 2014 | 1.72 | 1.77 | 1.63 |
|  | 2015 | 1.70 | 1.75 | 1.61 |
|  | 2016 | 1.73 | 1.79 | 1.64 |
|  | 2017 | 1.66 | 1.71 | 1.58 |
|  | 2018 | 1.63 | 1.70 | 1.55 |
|  | 2019 | 1.63 | 1.70 | 1.54 |
|  | 2020 | 1.64 | 1.78 | 1.51 |
|  | 2021 | 1.65 | 1.85 | 1.47 |
| **Central Sub-Saharan Africa** | 1990 | 11.36 | 14.21 | 8.50 |
|  | 1991 | 11.30 | 14.18 | 8.50 |
|  | 1992 | 11.19 | 14.04 | 8.42 |
|  | 1993 | 11.09 | 13.88 | 8.28 |
|  | 1994 | 11.09 | 13.88 | 8.21 |
|  | 1995 | 11.11 | 13.80 | 8.13 |
|  | 1996 | 11.16 | 13.96 | 8.16 |
|  | 1997 | 10.87 | 13.58 | 7.88 |
|  | 1998 | 10.81 | 13.55 | 7.77 |
|  | 1999 | 10.69 | 13.29 | 7.64 |
|  | 2000 | 10.60 | 13.13 | 7.53 |
|  | 2001 | 10.43 | 12.85 | 7.40 |
|  | 2002 | 10.05 | 12.45 | 7.23 |
|  | 2003 | 9.93 | 12.29 | 7.10 |
|  | 2004 | 9.72 | 12.06 | 6.99 |
|  | 2005 | 9.49 | 11.73 | 6.90 |
|  | 2006 | 9.39 | 11.68 | 6.93 |
|  | 2007 | 9.27 | 11.49 | 6.77 |
|  | 2008 | 9.24 | 11.51 | 6.75 |
|  | 2009 | 9.19 | 11.42 | 6.73 |
|  | 2010 | 9.18 | 11.49 | 6.66 |
|  | 2011 | 9.18 | 11.58 | 6.65 |
|  | 2012 | 9.16 | 11.60 | 6.63 |
|  | 2013 | 9.13 | 11.56 | 6.66 |
|  | 2014 | 9.08 | 11.63 | 6.65 |
|  | 2015 | 9.04 | 11.67 | 6.67 |
|  | 2016 | 9.00 | 11.72 | 6.63 |
|  | 2017 | 8.96 | 11.77 | 6.60 |
|  | 2018 | 8.92 | 11.69 | 6.52 |
|  | 2019 | 8.91 | 11.67 | 6.58 |
|  | 2020 | 8.94 | 11.67 | 6.48 |
|  | 2021 | 8.89 | 11.50 | 6.44 |
| **East Asia** | 1990 | 25.43 | 29.31 | 21.28 |
|  | 1991 | 25.09 | 28.71 | 21.16 |
|  | 1992 | 24.70 | 28.33 | 20.91 |
|  | 1993 | 24.36 | 27.64 | 20.61 |
|  | 1994 | 24.01 | 26.97 | 20.42 |
|  | 1995 | 23.83 | 26.51 | 19.90 |
|  | 1996 | 23.33 | 26.01 | 19.65 |
|  | 1997 | 22.93 | 25.53 | 19.58 |
|  | 1998 | 22.77 | 25.27 | 19.01 |
|  | 1999 | 22.74 | 25.28 | 19.11 |
|  | 2000 | 23.14 | 25.91 | 19.66 |
|  | 2001 | 23.22 | 26.05 | 19.68 |
|  | 2002 | 23.00 | 25.54 | 19.37 |
|  | 2003 | 23.14 | 25.52 | 19.46 |
|  | 2004 | 23.23 | 25.77 | 19.67 |
|  | 2005 | 22.42 | 24.85 | 19.04 |
|  | 2006 | 20.66 | 22.94 | 17.60 |
|  | 2007 | 19.57 | 21.78 | 16.67 |
|  | 2008 | 18.90 | 20.93 | 16.58 |
|  | 2009 | 18.37 | 20.32 | 15.96 |
|  | 2010 | 17.81 | 19.94 | 15.31 |
|  | 2011 | 17.13 | 19.08 | 15.05 |
|  | 2012 | 16.43 | 18.58 | 14.41 |
|  | 2013 | 15.72 | 17.52 | 13.84 |
|  | 2014 | 14.98 | 17.10 | 13.00 |
|  | 2015 | 14.56 | 16.61 | 12.60 |
|  | 2016 | 14.34 | 16.60 | 12.18 |
|  | 2017 | 14.05 | 16.73 | 11.83 |
|  | 2018 | 13.97 | 16.62 | 11.45 |
|  | 2019 | 13.96 | 16.68 | 11.55 |
|  | 2020 | 13.97 | 16.65 | 11.36 |
|  | 2021 | 13.91 | 16.84 | 11.23 |
| **Eastern Europe** | 1990 | 4.41 | 4.49 | 4.30 |
|  | 1991 | 4.41 | 4.50 | 4.31 |
|  | 1992 | 4.48 | 4.57 | 4.38 |
|  | 1993 | 4.70 | 4.78 | 4.59 |
|  | 1994 | 4.66 | 4.75 | 4.55 |
|  | 1995 | 4.34 | 4.43 | 4.25 |
|  | 1996 | 4.09 | 4.17 | 4.00 |
|  | 1997 | 3.87 | 3.94 | 3.79 |
|  | 1998 | 3.75 | 3.82 | 3.66 |
|  | 1999 | 3.80 | 3.87 | 3.72 |
|  | 2000 | 3.76 | 3.82 | 3.68 |
|  | 2001 | 3.67 | 3.73 | 3.59 |
|  | 2002 | 3.52 | 3.58 | 3.45 |
|  | 2003 | 3.48 | 3.53 | 3.41 |
|  | 2004 | 3.41 | 3.47 | 3.34 |
|  | 2005 | 3.43 | 3.49 | 3.35 |
|  | 2006 | 3.22 | 3.27 | 3.15 |
|  | 2007 | 3.17 | 3.23 | 3.11 |
|  | 2008 | 3.22 | 3.27 | 3.16 |
|  | 2009 | 3.12 | 3.17 | 3.06 |
|  | 2010 | 3.19 | 3.24 | 3.13 |
|  | 2011 | 3.06 | 3.11 | 3.00 |
|  | 2012 | 2.99 | 3.05 | 2.93 |
|  | 2013 | 3.00 | 3.06 | 2.94 |
|  | 2014 | 3.02 | 3.07 | 2.95 |
|  | 2015 | 3.07 | 3.13 | 3.00 |
|  | 2016 | 3.06 | 3.12 | 2.99 |
|  | 2017 | 2.98 | 3.06 | 2.88 |
|  | 2018 | 3.04 | 3.15 | 2.93 |
|  | 2019 | 3.05 | 3.18 | 2.93 |
|  | 2020 | 2.99 | 3.15 | 2.85 |
|  | 2021 | 2.94 | 3.19 | 2.68 |
| **Eastern Sub-Saharan Africa** | 1990 | 14.52 | 16.70 | 11.97 |
|  | 1991 | 14.54 | 16.66 | 11.98 |
|  | 1992 | 14.55 | 16.64 | 12.06 |
|  | 1993 | 14.61 | 16.61 | 12.08 |
|  | 1994 | 14.59 | 16.54 | 12.13 |
|  | 1995 | 14.55 | 16.42 | 12.06 |
|  | 1996 | 14.49 | 16.30 | 12.06 |
|  | 1997 | 14.48 | 16.25 | 12.02 |
|  | 1998 | 14.50 | 16.21 | 12.21 |
|  | 1999 | 14.35 | 16.11 | 12.05 |
|  | 2000 | 14.29 | 16.03 | 12.00 |
|  | 2001 | 14.05 | 15.90 | 11.77 |
|  | 2002 | 13.90 | 15.73 | 11.78 |
|  | 2003 | 13.78 | 15.67 | 11.79 |
|  | 2004 | 13.65 | 15.56 | 11.72 |
|  | 2005 | 13.49 | 15.41 | 11.48 |
|  | 2006 | 13.30 | 15.25 | 11.25 |
|  | 2007 | 13.04 | 14.89 | 11.15 |
|  | 2008 | 12.83 | 14.64 | 10.97 |
|  | 2009 | 12.70 | 14.53 | 10.91 |
|  | 2010 | 12.61 | 14.57 | 10.95 |
|  | 2011 | 12.43 | 14.37 | 10.83 |
|  | 2012 | 12.27 | 14.28 | 10.63 |
|  | 2013 | 12.15 | 14.08 | 10.45 |
|  | 2014 | 12.04 | 14.11 | 10.27 |
|  | 2015 | 11.98 | 14.11 | 10.17 |
|  | 2016 | 11.92 | 14.05 | 9.97 |
|  | 2017 | 11.88 | 14.03 | 9.93 |
|  | 2018 | 11.86 | 14.24 | 9.94 |
|  | 2019 | 11.85 | 14.17 | 9.99 |
|  | 2020 | 11.83 | 14.15 | 10.09 |
|  | 2021 | 11.74 | 14.12 | 9.82 |
| **High-income Asia Pacific** | 1990 | 5.13 | 5.37 | 4.87 |
|  | 1991 | 5.14 | 5.38 | 4.87 |
|  | 1992 | 5.13 | 5.35 | 4.87 |
|  | 1993 | 5.08 | 5.30 | 4.82 |
|  | 1994 | 5.02 | 5.23 | 4.74 |
|  | 1995 | 4.94 | 5.13 | 4.67 |
|  | 1996 | 4.94 | 5.11 | 4.65 |
|  | 1997 | 4.89 | 5.07 | 4.59 |
|  | 1998 | 4.88 | 5.06 | 4.60 |
|  | 1999 | 4.87 | 5.04 | 4.59 |
|  | 2000 | 4.81 | 4.99 | 4.52 |
|  | 2001 | 4.77 | 4.94 | 4.49 |
|  | 2002 | 4.65 | 4.83 | 4.36 |
|  | 2003 | 4.62 | 4.80 | 4.32 |
|  | 2004 | 4.54 | 4.72 | 4.23 |
|  | 2005 | 4.47 | 4.65 | 4.18 |
|  | 2006 | 4.39 | 4.57 | 4.11 |
|  | 2007 | 4.34 | 4.53 | 4.08 |
|  | 2008 | 4.25 | 4.44 | 3.99 |
|  | 2009 | 4.19 | 4.37 | 3.93 |
|  | 2010 | 4.16 | 4.34 | 3.90 |
|  | 2011 | 4.12 | 4.30 | 3.87 |
|  | 2012 | 3.96 | 4.14 | 3.72 |
|  | 2013 | 3.86 | 4.04 | 3.61 |
|  | 2014 | 3.79 | 3.96 | 3.53 |
|  | 2015 | 3.74 | 3.92 | 3.47 |
|  | 2016 | 3.67 | 3.85 | 3.39 |
|  | 2017 | 3.57 | 3.74 | 3.28 |
|  | 2018 | 3.49 | 3.67 | 3.19 |
|  | 2019 | 3.47 | 3.65 | 3.16 |
|  | 2020 | 3.39 | 3.58 | 3.07 |
|  | 2021 | 3.42 | 3.62 | 3.10 |
| **High-income North America** | 1990 | 3.73 | 3.82 | 3.55 |
|  | 1991 | 3.77 | 3.87 | 3.59 |
|  | 1992 | 3.80 | 3.89 | 3.61 |
|  | 1993 | 3.86 | 3.96 | 3.67 |
|  | 1994 | 3.91 | 4.01 | 3.72 |
|  | 1995 | 3.92 | 4.02 | 3.73 |
|  | 1996 | 3.92 | 4.02 | 3.72 |
|  | 1997 | 3.89 | 4.00 | 3.69 |
|  | 1998 | 3.94 | 4.05 | 3.74 |
|  | 1999 | 4.03 | 4.14 | 3.82 |
|  | 2000 | 4.08 | 4.19 | 3.88 |
|  | 2001 | 4.11 | 4.22 | 3.89 |
|  | 2002 | 4.12 | 4.22 | 3.91 |
|  | 2003 | 4.11 | 4.22 | 3.89 |
|  | 2004 | 4.06 | 4.17 | 3.84 |
|  | 2005 | 4.07 | 4.18 | 3.85 |
|  | 2006 | 4.08 | 4.20 | 3.86 |
|  | 2007 | 4.01 | 4.13 | 3.80 |
|  | 2008 | 3.97 | 4.09 | 3.76 |
|  | 2009 | 3.94 | 4.06 | 3.72 |
|  | 2010 | 3.94 | 4.06 | 3.71 |
|  | 2011 | 3.91 | 4.04 | 3.69 |
|  | 2012 | 3.87 | 3.99 | 3.65 |
|  | 2013 | 3.82 | 3.93 | 3.59 |
|  | 2014 | 3.79 | 3.91 | 3.57 |
|  | 2015 | 3.76 | 3.88 | 3.54 |
|  | 2016 | 3.77 | 3.89 | 3.55 |
|  | 2017 | 3.71 | 3.82 | 3.48 |
|  | 2018 | 3.64 | 3.75 | 3.42 |
|  | 2019 | 3.64 | 3.75 | 3.41 |
|  | 2020 | 3.58 | 3.70 | 3.36 |
|  | 2021 | 3.62 | 3.76 | 3.40 |
| **North Africa and Middle East** | 1990 | 2.79 | 3.19 | 2.26 |
|  | 1991 | 2.76 | 3.13 | 2.23 |
|  | 1992 | 2.74 | 3.10 | 2.22 |
|  | 1993 | 2.74 | 3.08 | 2.22 |
|  | 1994 | 2.73 | 3.06 | 2.22 |
|  | 1995 | 2.71 | 3.04 | 2.21 |
|  | 1996 | 2.69 | 3.01 | 2.21 |
|  | 1997 | 2.67 | 2.99 | 2.19 |
|  | 1998 | 2.64 | 2.96 | 2.16 |
|  | 1999 | 2.61 | 2.93 | 2.13 |
|  | 2000 | 2.57 | 2.88 | 2.11 |
|  | 2001 | 2.56 | 2.84 | 2.10 |
|  | 2002 | 2.52 | 2.78 | 2.08 |
|  | 2003 | 2.49 | 2.74 | 2.06 |
|  | 2004 | 2.46 | 2.71 | 2.04 |
|  | 2005 | 2.43 | 2.68 | 2.03 |
|  | 2006 | 2.41 | 2.63 | 2.02 |
|  | 2007 | 2.37 | 2.59 | 2.00 |
|  | 2008 | 2.34 | 2.56 | 1.98 |
|  | 2009 | 2.33 | 2.54 | 1.98 |
|  | 2010 | 2.29 | 2.50 | 1.96 |
|  | 2011 | 2.26 | 2.46 | 1.93 |
|  | 2012 | 2.23 | 2.43 | 1.91 |
|  | 2013 | 2.21 | 2.42 | 1.91 |
|  | 2014 | 2.22 | 2.42 | 1.91 |
|  | 2015 | 2.23 | 2.44 | 1.92 |
|  | 2016 | 2.22 | 2.44 | 1.93 |
|  | 2017 | 2.20 | 2.43 | 1.91 |
|  | 2018 | 2.18 | 2.41 | 1.89 |
|  | 2019 | 2.18 | 2.41 | 1.88 |
|  | 2020 | 2.13 | 2.38 | 1.83 |
|  | 2021 | 2.11 | 2.36 | 1.81 |
| **Oceania** | 1990 | 2.29 | 2.95 | 1.80 |
|  | 1991 | 2.29 | 2.92 | 1.83 |
|  | 1992 | 2.27 | 2.88 | 1.81 |
|  | 1993 | 2.26 | 2.85 | 1.80 |
|  | 1994 | 2.24 | 2.82 | 1.81 |
|  | 1995 | 2.22 | 2.78 | 1.81 |
|  | 1996 | 2.22 | 2.76 | 1.80 |
|  | 1997 | 2.20 | 2.71 | 1.79 |
|  | 1998 | 2.17 | 2.66 | 1.77 |
|  | 1999 | 2.16 | 2.64 | 1.78 |
|  | 2000 | 2.17 | 2.64 | 1.79 |
|  | 2001 | 2.14 | 2.60 | 1.75 |
|  | 2002 | 2.12 | 2.56 | 1.75 |
|  | 2003 | 2.12 | 2.55 | 1.76 |
|  | 2004 | 2.09 | 2.52 | 1.74 |
|  | 2005 | 2.09 | 2.53 | 1.74 |
|  | 2006 | 2.11 | 2.57 | 1.77 |
|  | 2007 | 2.10 | 2.58 | 1.75 |
|  | 2008 | 2.08 | 2.52 | 1.74 |
|  | 2009 | 2.05 | 2.49 | 1.71 |
|  | 2010 | 2.04 | 2.53 | 1.70 |
|  | 2011 | 2.05 | 2.54 | 1.70 |
|  | 2012 | 2.02 | 2.50 | 1.66 |
|  | 2013 | 2.00 | 2.49 | 1.64 |
|  | 2014 | 1.99 | 2.47 | 1.63 |
|  | 2015 | 2.00 | 2.45 | 1.64 |
|  | 2016 | 1.98 | 2.42 | 1.62 |
|  | 2017 | 1.98 | 2.43 | 1.60 |
|  | 2018 | 1.98 | 2.46 | 1.58 |
|  | 2019 | 1.99 | 2.46 | 1.58 |
|  | 2020 | 1.96 | 2.47 | 1.56 |
|  | 2021 | 1.95 | 2.45 | 1.54 |
| **South Asia** | 1990 | 4.17 | 5.06 | 3.66 |
|  | 1991 | 4.17 | 5.05 | 3.64 |
|  | 1992 | 4.23 | 5.06 | 3.76 |
|  | 1993 | 4.22 | 5.07 | 3.75 |
|  | 1994 | 4.27 | 5.12 | 3.80 |
|  | 1995 | 4.35 | 5.24 | 3.86 |
|  | 1996 | 4.34 | 5.14 | 3.87 |
|  | 1997 | 4.33 | 5.06 | 3.86 |
|  | 1998 | 4.31 | 5.13 | 3.84 |
|  | 1999 | 4.17 | 4.91 | 3.71 |
|  | 2000 | 4.12 | 4.88 | 3.67 |
|  | 2001 | 4.09 | 4.87 | 3.68 |
|  | 2002 | 4.02 | 4.79 | 3.63 |
|  | 2003 | 3.99 | 4.74 | 3.53 |
|  | 2004 | 3.91 | 4.69 | 3.50 |
|  | 2005 | 3.89 | 4.64 | 3.52 |
|  | 2006 | 3.83 | 4.61 | 3.40 |
|  | 2007 | 3.83 | 4.66 | 3.47 |
|  | 2008 | 3.78 | 4.59 | 3.44 |
|  | 2009 | 3.67 | 4.40 | 3.34 |
|  | 2010 | 3.64 | 4.41 | 3.30 |
|  | 2011 | 3.59 | 4.39 | 3.24 |
|  | 2012 | 3.49 | 4.32 | 3.16 |
|  | 2013 | 3.53 | 4.41 | 3.18 |
|  | 2014 | 3.55 | 4.37 | 3.22 |
|  | 2015 | 3.56 | 4.38 | 3.23 |
|  | 2016 | 3.55 | 4.41 | 3.21 |
|  | 2017 | 3.58 | 4.37 | 3.21 |
|  | 2018 | 3.61 | 4.48 | 3.26 |
|  | 2019 | 3.61 | 4.38 | 3.25 |
|  | 2020 | 3.57 | 4.23 | 3.21 |
|  | 2021 | 3.54 | 4.26 | 3.12 |
| **Southeast Asia** | 1990 | 2.83 | 3.30 | 2.39 |
|  | 1991 | 2.81 | 3.26 | 2.38 |
|  | 1992 | 2.80 | 3.20 | 2.40 |
|  | 1993 | 2.80 | 3.18 | 2.40 |
|  | 1994 | 2.81 | 3.17 | 2.40 |
|  | 1995 | 2.81 | 3.14 | 2.44 |
|  | 1996 | 2.81 | 3.13 | 2.43 |
|  | 1997 | 2.75 | 3.04 | 2.39 |
|  | 1998 | 2.76 | 3.10 | 2.39 |
|  | 1999 | 2.77 | 3.08 | 2.41 |
|  | 2000 | 2.75 | 3.06 | 2.42 |
|  | 2001 | 2.71 | 3.00 | 2.38 |
|  | 2002 | 2.71 | 3.00 | 2.38 |
|  | 2003 | 2.70 | 2.98 | 2.38 |
|  | 2004 | 2.70 | 2.98 | 2.39 |
|  | 2005 | 2.70 | 2.99 | 2.41 |
|  | 2006 | 2.68 | 2.96 | 2.41 |
|  | 2007 | 2.66 | 2.89 | 2.40 |
|  | 2008 | 2.64 | 2.89 | 2.42 |
|  | 2009 | 2.61 | 2.86 | 2.39 |
|  | 2010 | 2.60 | 2.85 | 2.39 |
|  | 2011 | 2.57 | 2.84 | 2.37 |
|  | 2012 | 2.52 | 2.81 | 2.33 |
|  | 2013 | 2.49 | 2.76 | 2.29 |
|  | 2014 | 2.48 | 2.74 | 2.26 |
|  | 2015 | 2.47 | 2.71 | 2.26 |
|  | 2016 | 2.46 | 2.73 | 2.24 |
|  | 2017 | 2.44 | 2.73 | 2.22 |
|  | 2018 | 2.45 | 2.71 | 2.22 |
|  | 2019 | 2.46 | 2.76 | 2.21 |
|  | 2020 | 2.43 | 2.76 | 2.14 |
|  | 2021 | 2.44 | 2.78 | 2.13 |
| **Southern Latin America** | 1990 | 7.75 | 8.11 | 7.38 |
|  | 1991 | 7.63 | 7.98 | 7.26 |
|  | 1992 | 7.51 | 7.85 | 7.14 |
|  | 1993 | 7.34 | 7.67 | 6.98 |
|  | 1994 | 7.26 | 7.57 | 6.90 |
|  | 1995 | 7.00 | 7.28 | 6.66 |
|  | 1996 | 7.18 | 7.47 | 6.82 |
|  | 1997 | 7.49 | 7.82 | 7.12 |
|  | 1998 | 7.48 | 7.81 | 7.09 |
|  | 1999 | 7.54 | 7.89 | 7.16 |
|  | 2000 | 7.35 | 7.71 | 6.96 |
|  | 2001 | 7.14 | 7.48 | 6.77 |
|  | 2002 | 7.06 | 7.38 | 6.70 |
|  | 2003 | 6.94 | 7.25 | 6.58 |
|  | 2004 | 6.70 | 6.99 | 6.35 |
|  | 2005 | 6.56 | 6.86 | 6.21 |
|  | 2006 | 6.39 | 6.70 | 6.05 |
|  | 2007 | 6.31 | 6.62 | 5.97 |
|  | 2008 | 5.94 | 6.22 | 5.63 |
|  | 2009 | 5.72 | 5.98 | 5.41 |
|  | 2010 | 5.49 | 5.74 | 5.19 |
|  | 2011 | 5.44 | 5.69 | 5.14 |
|  | 2012 | 5.32 | 5.55 | 5.02 |
|  | 2013 | 5.15 | 5.40 | 4.86 |
|  | 2014 | 4.99 | 5.23 | 4.70 |
|  | 2015 | 4.86 | 5.08 | 4.59 |
|  | 2016 | 4.79 | 5.02 | 4.52 |
|  | 2017 | 4.58 | 4.81 | 4.31 |
|  | 2018 | 4.52 | 4.75 | 4.26 |
|  | 2019 | 4.47 | 4.70 | 4.20 |
|  | 2020 | 4.30 | 4.56 | 4.03 |
|  | 2021 | 4.07 | 4.35 | 3.77 |
| **Southern Sub-Saharan Africa** | 1990 | 11.89 | 13.90 | 10.59 |
|  | 1991 | 12.07 | 14.09 | 10.65 |
|  | 1992 | 12.59 | 14.43 | 11.21 |
|  | 1993 | 12.56 | 14.47 | 11.20 |
|  | 1994 | 13.27 | 15.08 | 11.99 |
|  | 1995 | 13.77 | 15.57 | 12.27 |
|  | 1996 | 15.00 | 16.55 | 13.56 |
|  | 1997 | 16.37 | 17.73 | 14.91 |
|  | 1998 | 17.06 | 18.43 | 15.43 |
|  | 1999 | 16.48 | 17.64 | 14.84 |
|  | 2000 | 17.01 | 18.17 | 15.21 |
|  | 2001 | 16.70 | 17.88 | 15.03 |
|  | 2002 | 16.80 | 17.99 | 15.05 |
|  | 2003 | 16.92 | 18.12 | 15.14 |
|  | 2004 | 16.58 | 17.73 | 14.83 |
|  | 2005 | 16.35 | 17.51 | 14.66 |
|  | 2006 | 16.18 | 17.38 | 14.42 |
|  | 2007 | 15.67 | 16.78 | 14.11 |
|  | 2008 | 15.37 | 16.46 | 13.88 |
|  | 2009 | 15.10 | 16.17 | 13.57 |
|  | 2010 | 14.71 | 15.83 | 13.30 |
|  | 2011 | 14.16 | 15.17 | 12.84 |
|  | 2012 | 13.70 | 14.69 | 12.43 |
|  | 2013 | 13.33 | 14.26 | 12.11 |
|  | 2014 | 13.21 | 14.20 | 12.03 |
|  | 2015 | 13.16 | 14.14 | 12.01 |
|  | 2016 | 12.95 | 13.94 | 11.83 |
|  | 2017 | 12.50 | 13.44 | 11.42 |
|  | 2018 | 12.29 | 13.22 | 11.27 |
|  | 2019 | 11.86 | 12.80 | 10.85 |
|  | 2020 | 11.87 | 12.88 | 10.81 |
|  | 2021 | 11.69 | 12.72 | 10.68 |
| **Tropical Latin America** | 1990 | 7.04 | 7.27 | 6.68 |
|  | 1991 | 6.91 | 7.14 | 6.56 |
|  | 1992 | 6.94 | 7.14 | 6.59 |
|  | 1993 | 7.02 | 7.23 | 6.68 |
|  | 1994 | 6.88 | 7.08 | 6.55 |
|  | 1995 | 6.74 | 6.93 | 6.40 |
|  | 1996 | 6.65 | 6.85 | 6.31 |
|  | 1997 | 6.56 | 6.75 | 6.25 |
|  | 1998 | 6.54 | 6.72 | 6.22 |
|  | 1999 | 6.53 | 6.72 | 6.22 |
|  | 2000 | 6.46 | 6.66 | 6.16 |
|  | 2001 | 6.44 | 6.64 | 6.15 |
|  | 2002 | 6.45 | 6.63 | 6.16 |
|  | 2003 | 6.48 | 6.67 | 6.20 |
|  | 2004 | 6.51 | 6.71 | 6.23 |
|  | 2005 | 6.33 | 6.52 | 6.04 |
|  | 2006 | 6.30 | 6.49 | 5.99 |
|  | 2007 | 6.21 | 6.40 | 5.91 |
|  | 2008 | 6.13 | 6.32 | 5.82 |
|  | 2009 | 6.07 | 6.26 | 5.78 |
|  | 2010 | 5.99 | 6.19 | 5.71 |
|  | 2011 | 5.89 | 6.08 | 5.59 |
|  | 2012 | 5.77 | 5.96 | 5.46 |
|  | 2013 | 5.67 | 5.85 | 5.37 |
|  | 2014 | 5.62 | 5.80 | 5.30 |
|  | 2015 | 5.56 | 5.74 | 5.25 |
|  | 2016 | 5.52 | 5.70 | 5.20 |
|  | 2017 | 5.35 | 5.53 | 5.04 |
|  | 2018 | 5.25 | 5.43 | 4.94 |
|  | 2019 | 5.21 | 5.39 | 4.93 |
|  | 2020 | 5.20 | 5.41 | 4.91 |
|  | 2021 | 5.07 | 5.29 | 4.78 |
| **Western Europe** | 1990 | 4.80 | 4.92 | 4.62 |
|  | 1991 | 4.77 | 4.89 | 4.58 |
|  | 1992 | 4.77 | 4.88 | 4.59 |
|  | 1993 | 4.80 | 4.93 | 4.62 |
|  | 1994 | 4.79 | 4.91 | 4.61 |
|  | 1995 | 4.78 | 4.90 | 4.60 |
|  | 1996 | 4.75 | 4.87 | 4.57 |
|  | 1997 | 4.67 | 4.79 | 4.48 |
|  | 1998 | 4.65 | 4.77 | 4.44 |
|  | 1999 | 4.62 | 4.74 | 4.42 |
|  | 2000 | 4.57 | 4.70 | 4.37 |
|  | 2001 | 4.56 | 4.69 | 4.35 |
|  | 2002 | 4.55 | 4.68 | 4.34 |
|  | 2003 | 4.51 | 4.63 | 4.31 |
|  | 2004 | 4.40 | 4.54 | 4.21 |
|  | 2005 | 4.37 | 4.50 | 4.16 |
|  | 2006 | 4.29 | 4.42 | 4.08 |
|  | 2007 | 4.25 | 4.37 | 4.03 |
|  | 2008 | 4.21 | 4.34 | 3.99 |
|  | 2009 | 4.16 | 4.29 | 3.93 |
|  | 2010 | 4.11 | 4.24 | 3.88 |
|  | 2011 | 3.99 | 4.12 | 3.75 |
|  | 2012 | 3.88 | 4.01 | 3.65 |
|  | 2013 | 3.81 | 3.93 | 3.57 |
|  | 2014 | 3.85 | 3.98 | 3.62 |
|  | 2015 | 3.92 | 4.05 | 3.67 |
|  | 2016 | 3.91 | 4.05 | 3.67 |
|  | 2017 | 3.88 | 4.02 | 3.65 |
|  | 2018 | 3.89 | 4.03 | 3.65 |
|  | 2019 | 3.84 | 3.98 | 3.60 |
|  | 2020 | 3.68 | 3.83 | 3.44 |
|  | 2021 | 3.65 | 3.81 | 3.41 |
| **Western Sub-Saharan Africa** | 1990 | 2.86 | 3.44 | 2.34 |
|  | 1991 | 2.84 | 3.36 | 2.38 |
|  | 1992 | 2.84 | 3.33 | 2.34 |
|  | 1993 | 2.83 | 3.33 | 2.33 |
|  | 1994 | 2.83 | 3.35 | 2.31 |
|  | 1995 | 2.82 | 3.30 | 2.35 |
|  | 1996 | 2.80 | 3.29 | 2.31 |
|  | 1997 | 2.80 | 3.29 | 2.34 |
|  | 1998 | 2.80 | 3.29 | 2.32 |
|  | 1999 | 2.82 | 3.35 | 2.34 |
|  | 2000 | 2.87 | 3.41 | 2.36 |
|  | 2001 | 2.97 | 3.53 | 2.40 |
|  | 2002 | 3.13 | 3.71 | 2.55 |
|  | 2003 | 3.28 | 3.83 | 2.60 |
|  | 2004 | 3.42 | 4.07 | 2.74 |
|  | 2005 | 3.54 | 4.19 | 2.78 |
|  | 2006 | 3.68 | 4.28 | 2.87 |
|  | 2007 | 3.79 | 4.48 | 2.90 |
|  | 2008 | 3.89 | 4.67 | 2.96 |
|  | 2009 | 4.00 | 4.69 | 3.04 |
|  | 2010 | 4.12 | 4.83 | 3.18 |
|  | 2011 | 4.23 | 4.99 | 3.24 |
|  | 2012 | 4.31 | 5.07 | 3.33 |
|  | 2013 | 4.39 | 5.10 | 3.39 |
|  | 2014 | 4.44 | 5.20 | 3.41 |
|  | 2015 | 4.46 | 5.18 | 3.42 |
|  | 2016 | 4.51 | 5.30 | 3.51 |
|  | 2017 | 4.54 | 5.30 | 3.47 |
|  | 2018 | 4.56 | 5.45 | 3.48 |
|  | 2019 | 4.58 | 5.42 | 3.39 |
|  | 2020 | 4.57 | 5.49 | 3.42 |
|  | 2021 | 4.58 | 5.43 | 3.43 |

**GBD, Global Burden of Disease Study; SDI, Socio-demographic index.**

# Supplementary Figures


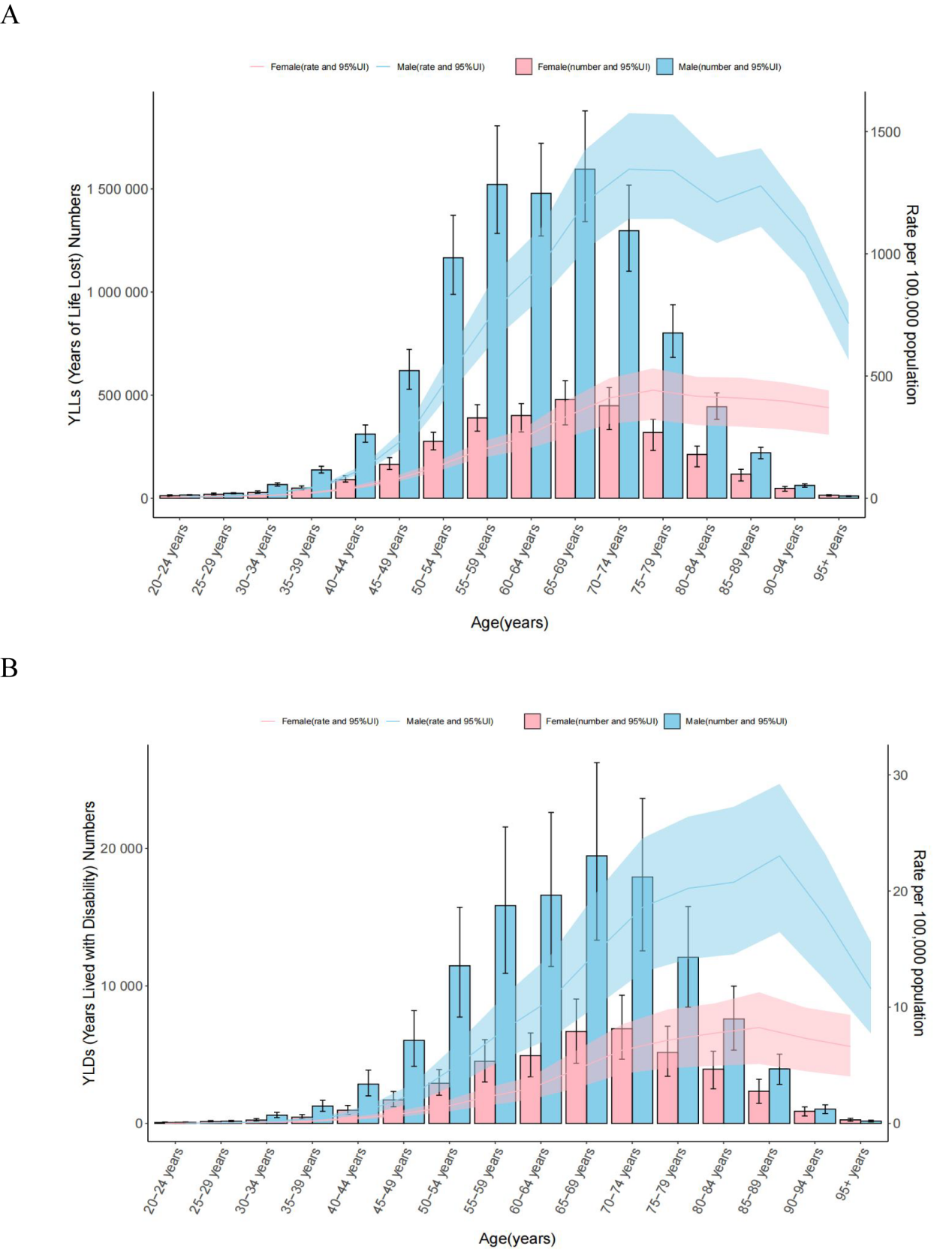


**Supplementary Figure 1**

The age-standardised rates of esophageal cancer in 2021 per 100,000 population for the 21 Global Burden of Disease regions, by sex. (A) YLLs (years of life lost); (B) YLDs (years lived with disability).


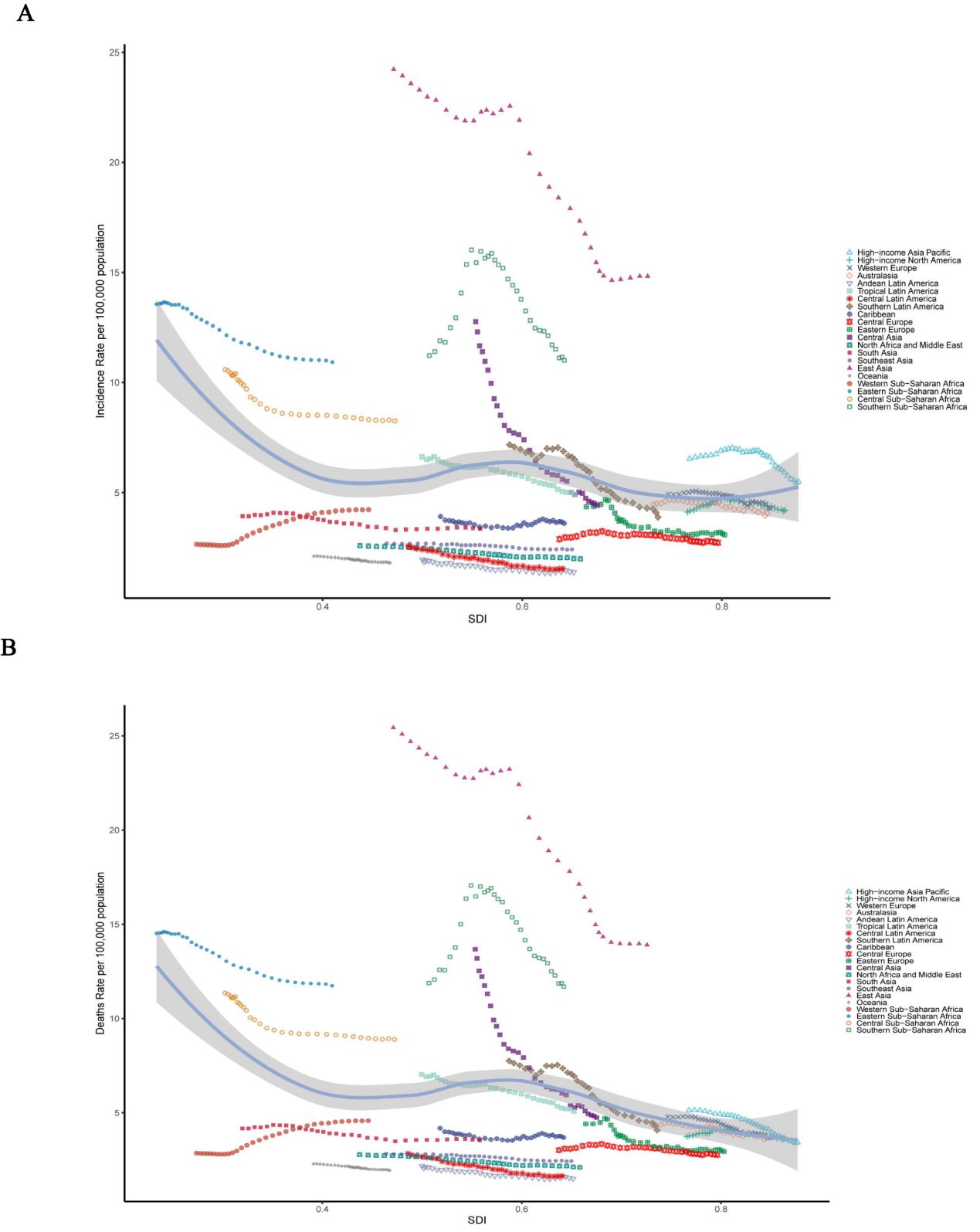


**Supplementary Figure 2**

Age-standardised rates of esophageal cancer (EC) from 1990 to 2021, globally and across 21 Global Burden of Disease (GBD) regions, by Socio-demographic Index (SDI). The black line represents the expected rates in 2021 based solely on SDI, with points depicting yearly estimates for each region from 1990 to 2021. (A) Incidence rates. (B) Death rates. GBD, Global Burden of Disease; EC, esophageal cancer; SDI, Socio-demographic Index.
